# Supplementary material for: Molecular networking and computational NMR analyses uncover six polyketide-terpene hybrids from termite-associated Xylaria isolates
Source: Commun Chem. 2024 Jun 7;7:129. doi: 10.1038/s42004-024-01210-6 (PMC11161606; doi:10.1038/s42004-024-01210-6)
Supplement: Supplementary file 2 — Supplementary Information [file 42004_2024_1210_MOESM2_ESM.pdf]

Supplementary Items

Figure S1. GNPS molecular network analysis of co-cultures. Overview comparisons of DCM extract of inhibition zone (pink color), X802 control (blue color) and T153 control (white color). Details have been deposited at Zenodo under the doi:10.5281/zenodo.10978114. 4
Figure S2. GNPS mediated molecular networking showing inhibition zone (pink color) and X802 control (blue color). A) pseudoxylallemycin cluster; B) cytochalasin cluster. 5
Figure S3. HR-ESIMS data of compound **1**. 8
Figure S4. ^{1}H NMR spectrum of compound **1**. 9
Figure S5. ^{1}H-^{1}H COSY spectrum of compound **1**. 10
Figure S6. ^{1}H-^{13}C HSQC spectrum of compound **1**. 11
Figure S7. ^{1}H-^{13}C HMBC spectrum of compound **1**. 12
Figure S8. ^{1}H-^{1}H ROESY spectrum of compound **1**. 13
Figure S9. DP4+ probability analysis for the determination of the absolute configuration of **1**. 17
Figure S10. HR-ESIMS data of compound **2**. 18
Figure S11. ^{1}H NMR spectrum of compound **2**. 19
Figure S12. ^{1}H-^{1}H COSY spectrum of compound **2**. 20
Figure S13. ^{1}H-^{13}C HSQC spectrum of compound **2**. 21
Figure S14. ^{1}H-^{13}C HMBC spectrum of compound **2**. 22
Figure S15. ^{1}H-^{1}H ROESY spectrum of compound **2**. 23
Figure S16. HR-ESIMS data of compound **3**. 24
Figure S17. ^{1}H NMR spectrum of compound **3**. 25
Figure S18. ^{1}H-^{1}H COSY spectrum of compound **3**. 26
Figure S19. ^{1}H-^{13}C HSQC spectrum of compound **3**. 27
Figure S20. ^{1}H-^{13}C HMBC spectrum of compound **3**. 28
Figure S21. ^{1}H-^{1}H ROESY spectrum of compound **3**. 29
Figure S22. DP4+ probability analysis for the determination of the absolute configuration of **2**. 36
Figure S23. DP4+ probability analysis for the determination of the absolute configuration of **3**. 39
Figure S24. HR-ESIMS data of compound **4**. 40
Figure S25. ^{1}H NMR spectrum of compound **4**. 41
Figure S26. ^{1}H-^{1}H COSY spectrum of compound **4**. 42
Figure S27. ^{1}H-^{13}C HSQC spectrum of compound **4**. 43
Figure S28. ^{1}H-^{13}C HMBC spectrum of compound **4**. 44
Figure S29. ^{1}H-^{1}H ROESY spectrum of compound **4**. 45
Figure S30. DP4+ probability analysis for the determination of the absolute configuration of **4**. 48
Figure S31. HR-ESIMS data of compound **5**. 49
Figure S32. ^{1}H NMR spectrum of compound **5**. 51
Figure S33. ^{1}H-^{1}H COSY spectrum of compound **5**. 52
Figure S34. ^{1}H-^{13}C HSQC spectrum of compound **5**. 53

Figure S35. ^{1}H-^{13}C HMBC spectrum of compound **5**. 54
Figure S36. ^{1}H-^{1}H ROESY spectrum of compound **5**. 55
Figure S37. DP4+ probability analysis for the determination of the absolute configuration of **5**. 58
Figure S38. HR-ESIMS data of compound **6**. 59
Figure S39. ^{1}H NMR spectrum of compound **6**. 60
Figure S40. ^{1}H-^{1}H COSY spectrum of compound **6**. 61
Figure S41. ^{1}H-^{13}C HSQC spectrum of compound **6**. 62
Figure S42. ^{1}H-^{13}C HMBC spectrum of compound **6**. 63
Figure S43. ^{1}H-^{1}H ROESY spectrum of compound **6**. 64
Figure S44. DP4+ probability analysis for the determination of the absolute configuration of **6**. 67
Figure S45. ^{1}H NMR spectrum of compound **7**. 68
Figure S46. ^{1}H-^{13}C HSQC spectrum of compound **7**. 69
Figure S47. ^{1}H-^{13}C HMBC spectrum of compound **7**. 70
Figure S48. HR-ESIMS data of compound **8**. 72
Figure S49. ^{1}H NMR spectrum of compound **8**. 73
Figure S50. ^{13}C NMR spectrum of compound **8**. 74
Figure S51. ^{1}H-^{1}H COSY spectrum of compound **8**. 75
Figure S52. ^{1}H-^{13}C HSQC spectrum of compound **8**. 76
Figure S53. ^{1}H-^{13}C HMBC spectrum of compound **8**. 77
Figure S54. ^{1}H-^{1}H ROESY spectrum of compound **8**. 78
Figure S55. DP4+ probability analysis for the determination of the absolute configuration of **8**. 81
Figure S56. HR-ESIMS data of compound **9**. 83
Figure S57. ^{1}H NMR spectrum of compound **9**. 84
Figure S58. ^{1}H-^{1}H COSY spectrum of compound **9**. 85
Figure S59. ^{1}H-^{13}C HSQC spectrum of compound **9**. 86
Figure S60. ^{1}H-^{13}C HMBC spectrum of compound **9**. 87
Figure S61. Refinement: All non H-atoms were located in the electron density maps and refined anisotropically. C-bound H atoms were placed in positions of optimized geometry and treated as riding atoms. Their isotropic displacement parameters were coupled to the corresponding carrier atoms by a factor of 1.2 (CH, CH2) or 1.5 (CH3). The O3 and N2 bonded H-atoms H3O and H2N were located in the electron density maps. Their positional parameters were refined using isotropic displacement parameters which were set at 1.2 (N2) and 1.5 (O3) times the Ueq value of the parent atoms. Restraints of 0.88(0.01) Å and 0.84(0.01) Å were used for the N-H and O-H bond lengths, respectively. 89
Figure S62. Antifungal activity assay of **2-7** against *Aspergillus nidulans* RMS011 (n = 3). 91

Table S1. ^{1}H NMR (800 MHz) data of compounds **1**–**6** in MeOH-*d*$_4$.[a] 6
Table S2. ^{13}C NMR (200 MHz) of compounds **1**–**6** in MeOH-*d*$_4$.[a] 7
Table S3. Comparison of NMR data of compound **1** and cytosporin E[1] in MeOH-*d*$_4$. 14
Table S4. Comparison of ^{1}H NMR data of compound **1** and calculated ^{1}H NMR data of six possible isomers of **1** in MeOH-*d*$_4$. 15
Table S5. Comparison of ^{13}C NMR data of compound **1** and calculated ^{13}C NMR data of 6 possible isomers of **1** in MeOH-*d*$_4$. 16
Table S6. Comparison of NMR data of compound **2** and cytosporin D[1] in MeOH-*d*$_4$. 30
Table S7. Comparison of NMR data of compound **2** and cytosporin M[2] in MeOH-*d*$_4$. 31
Table S8. Comparison of NMR data of compound **3** and cytosporin D[1] in MeOH-*d*$_4$. 32
Table S9. Comparison of NMR data of compound **3** and cytosporin M[2] in MeOH-*d*$_4$. 33
Table S10. Comparison of ^{1}H NMR data of compound **2** and calculated ^{1}H NMR data of 6 possible isomers of **2** in MeOH-*d*$_4$. 34
Table S11. Comparison of ^{13}C NMR data of compound **2** and calculated ^{13}C NMR data of 6 possible isomers of **1** in MeOH-*d*$_4$. 35
Table S12. Comparison of ^{1}H NMR data of compound **3** and calculated ^{1}H NMR data of 6 possible isomers of **3** in MeOH-*d*$_4$. 37
Table S13. Comparison of ^{13}C NMR data of compound **3** and calculated ^{13}C NMR data of 6 possible isomers of **3** in MeOH-*d*$_4$. 38
Table S14. Comparison of ^{1}H NMR data of compound **4** and calculated ^{1}H NMR data of 7 possible isomers of **4** in MeOH-*d*$_4$. 46
Table S15. Comparison of ^{13}C NMR data of compound **4** and calculated ^{13}C NMR data of 7 possible isomers of **4** in MeOH-*d*$_4$. 47
Table S16. Comparison of ^{1}H NMR data of compound **5** and calculated ^{1}H NMR data of 6 possible isomers of **5** in MeOH-*d*$_4$. 56
Table S17. Comparison of ^{13}C NMR data of compound **5** and calculated ^{13}C NMR data of 6 possible isomers of **5** in MeOH-*d*$_4$. 57
Table S18. Comparison of ^{1}H NMR data of compound **6** and calculated ^{1}H NMR data of 6 possible isomers of **6** in MeOH-*d*$_4$. 65
Table S19. Comparison of ^{13}C NMR data of compound **6** and calculated ^{13}C NMR data of 6 possible isomers of **6** in MeOH-*d*$_4$. 66
Table S20. ^{1}H and ^{13}C NMR (700 MHz) data of xylasporin I (**8**) in $CDCl_3$.[a] 71
Table S21. Comparison of ^{1}H NMR data of compound **8** and calculated ^{1}H NMR data of 10 possible isomers of **8** in $CDCl_3$. 79
Table S22. Comparison of ^{13}C NMR data of compound **8** and calculated ^{1}H NMR data of 10 possible isomers of **8** in $CDCl_3$. 80
Table S23. ^{1}H and ^{13}C NMR (700 MHz) data of xylacremolide B (**9**) in $CDCl_3$.[a] 82
Table S24. Crystallography data. Crystal data and structure refinement for compound 9. 88
Table S25. *In silico* analysis of the *px* gene cluster within the genome of *Xylaria* sp. X802.Homologues biosynthetic gene clusters were identified from the genome of *Xylaria* sp. X802 (*Xylaria* sp. X802, NCBI Accession Nr: JAJFDH000000000) using the protein-protein BLAST (blastp) function of the NCBI. RNA-Seq data was mapped against the identified *px* gene cluster and expression levels were calculated using Geneious Prime 2021.0.3 (Biomatters, Ltd.). 90

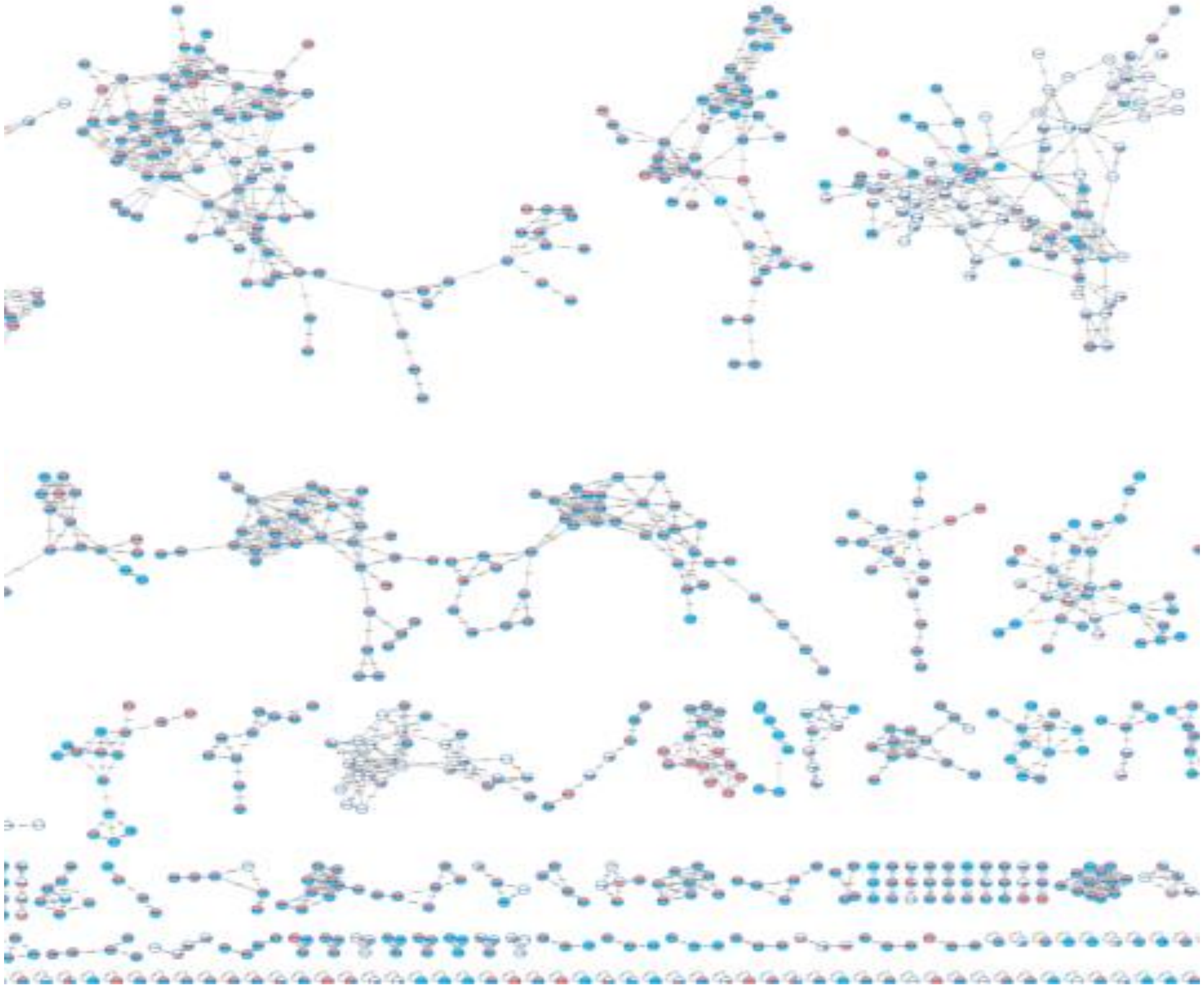

Figure S1. GNPS molecular network analysis of co-cultures. Overview comparisons of DCM extract of inhibition zone (pink color), X802 control (blue color) and T153 control (white color). Details have been deposited at Zenodo under the doi:10.5281/zenodo.10978114.

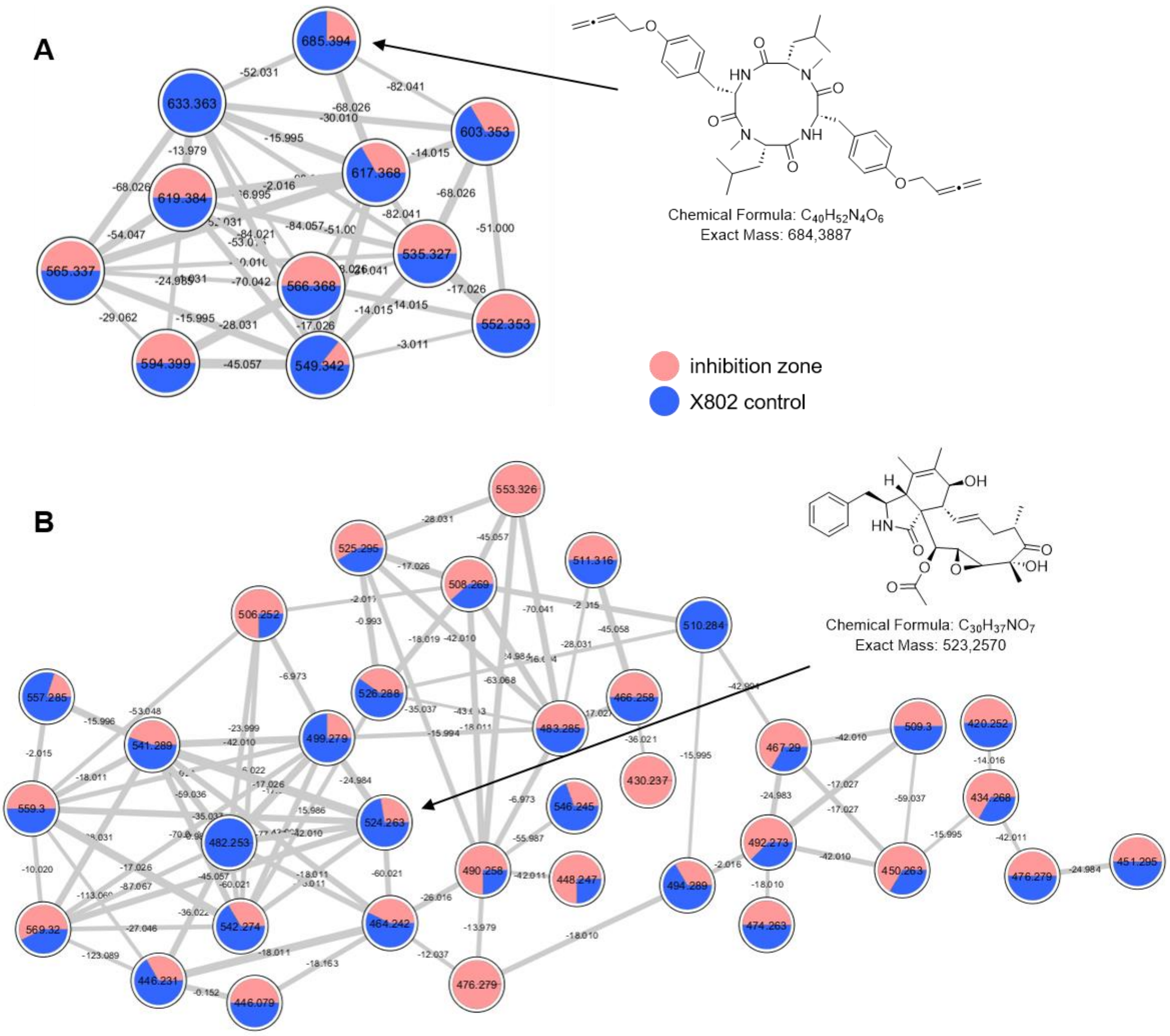

Figure S2. GNPS mediated molecular networking showing inhibition zone (pink color) and X802 control (blue color). A) pseudoxylallemycin cluster; B) cytochalasin cluster.

Table S1. ^{1}H NMR (800 MHz) data of compounds **1**–**6** in MeOH-d_4.[a]

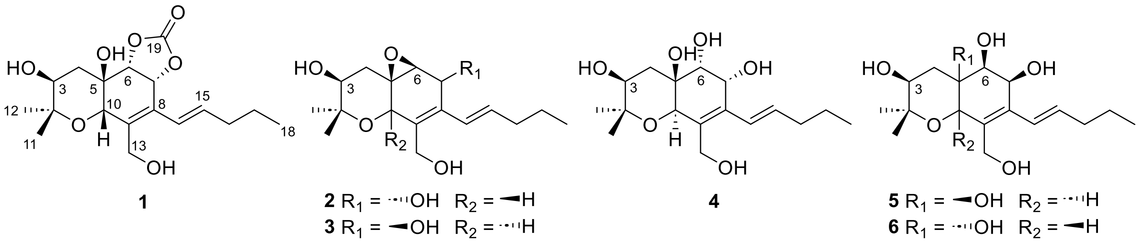

N°	1	2	3	4	5	6
	δ_H	δ_H	δ_H	δ_H	δ_H	δ_H
3	4.01 dd (12.0, 5.5)	3.63 dd (12.0, 5.0)	3.59 dd (10.5, 1.5)	3.86 dd (6.0, 2.5)	3.73 t (7.5)	3.97 dd (10.0, 4.5)
4*α*	1.89 dd (13.0, 12.0)	1.63 dd (12.5, 5.0)	2.36 dd (15.0, 1.5)	2.07 dd (14.0, 6.0)	1.81 dd (14.0, 7.5)	1.88 dd (13.5, 10.0)
4*β*	2.29 dd (13.0, 5.5)	2.23 t (12.5)	1.70 dd (15.0, 10.5)	1.83 dd (14.0, 2.5)	2.87 dd (14.0, 8.0)	2.28 dd (13.5, 4.5)
6	4.75 d (8.0)	3.22 brs	3.29 brs	3.97 d (6.5)	3.70 d (4.5)	3.62 d (4.5)
7	5.57 d (8.0)	4.61 brs	4.60 brs	4.05 d (6.5)	4.54 d (4.5)	4.48 d (4.5)
10	4.22 s	4.49 s	4.49 s	4.11 s	4.50 s	4.15 s
11	1.27 s	1.31 s	1.15 s	1.28 s	1.23 s	1.27 s
12	1.22 s	1.27 s	1.12 s	1.25 s	1.13 s	1.21 s
13	4.13 d (12.5), 4.45 d (12.5)	4.03 d (12.5), 4.38 d (12.5)	4.14 d (12.5), 4.38 d (12.5)	4.12 d (12.0), 4.40 d (12.0)	4.23 d (12.0), 4.38 d (12.0)	4.19 d (12.0), 4.36 d (12.0)
14	6.52 d (16.0)	6.44 d (16.0)	6.40 d (16.0)	6.23 d (16.0)	6.44 d (16.0)	6.27 d (16.0)
15	6.04 dt (16.0, 7.0)	6.11 dt (16.0, 7.0)	6.05 dt (16.0, 7.0)	6.01 dt (16.0, 7.0)	6.09 dt (16.0, 7.0)	6.03 dt (16.0, 7.0)
16	2.20 q (7.0)	2.16 m	2.13 m	2.15 m	2.16 m	2.17 m
17	1.50 m	1.47 m	1.44 m	1.47 m	1.48 m	1.48 m
18	0.96 t (7.5)	0.94 t (7.5)	0.91 t (7.5)	0.95 t (7.5)	0.96 t (7.5)	0.96 t (7.5)

[a] Coupling constants (in parentheses) are in Hz.

Table S2. ^{13}C NMR (200 MHz) of compounds **1**–**6** in MeOH-d_4.[a]

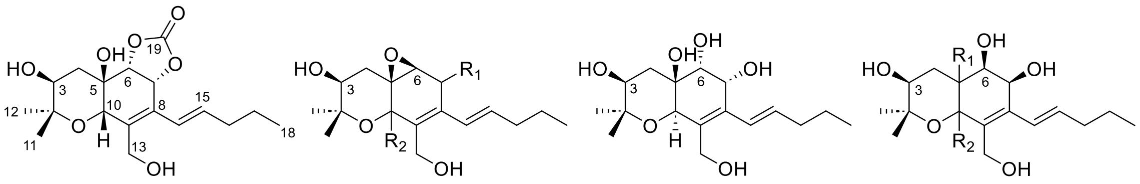

1 **2** R_1 = ···OH R_2 = H; **3** R_1 = OH R_2 = ···H **4** **5** R_1 = OH R_2 = ···H; **6** R_1 = ···OH R_2 = H

N°	1	2	3	4	5	6
	δ_C	δ_C	δ_C	δ_C	δ_C	δ_C
2	77.5, s	77.6, s	74.8, s	85.4 s	85.3 s	77.1 s
3	71.2, d	73.7, d	73.1, d	78.0 d	72.0 d	72.0 d
4	42.0, t	36.2, t	34.1, t	38.5 t	37.2 t	40.9 t
5	67.4, s	57.0, s	59.2, s	85.5 s	81.9 s	82.0 s
6	80.8, d	61.5, d	59.5, d	74.0 d	72.9 d	76.5 d
7	75.1, d	64.9, d	64.8, d	74.6 d	68.5 d	69.1 d
8	130.0, s	130.7, s	131.5, s	134.5 s	135.1 s	137.2 s
9	135.5, s	133.3, s	133.8, s	137.6 s	134.9 s	132.2 s
10	70.0, d	67.3, d	67.4, d	74.8 d	86.2 d	72.9 d
11	16.5, q	16.1, q	24.5, q	22.7 q	25.6 q	18.1 q
12	28.0, q	27.7, q	25.5, q	28.1 q	26.5 q	27.3 q
13	59.7, t	59.3, t	59.6, t	59.6 t	58.1 t	59.8 t
14	125.5, d	126.7, d	126.7, d	125.8 d	126.7 d	126.4 d
15	136.4, d	135.2, d	134.8, d	136.7 d	135.3 d	136.7 d
16	36.5, t	36.4, t	36.4, t	36.4 t	36.4 t	36.6 t
17	23.2, t	23.3, t	23.3, t	23.1 t	23.3 t	23.4 t
18	13.8, q	13.7, q	13.8, q	13.7 q	13.9 q	13.9 q
19	155.9, s					

[a] ^{13}C NMR data were assigned based on HSQC and HMBC experiments.

Single Mass Analysis
Tolerance = 5.0 mDa / DBE: min = -1.5, max = 50.0
Element prediction: Off
Number of isotope peaks used for i-FIT = 3
Monoisotopic Mass, Even Electron Ions
231 formula(e) evaluated with 2 results within limits (up to 50 closest results for each mass)
Elements Used:

Mass	Calc. Mass	mDa	PPM	DBE	Formula	i-FIT	i-FIT Norm	Fit Conf %	C	H	O
709.3430	709.3435	-0.5	-0.7	10.5	C36 H53 O14	293.2	0.000	100.00	36	53	14
	709.3470	-4.0	-5.6	32.5	C54 H45 O	305.8	12.536	0.00	54	45	1

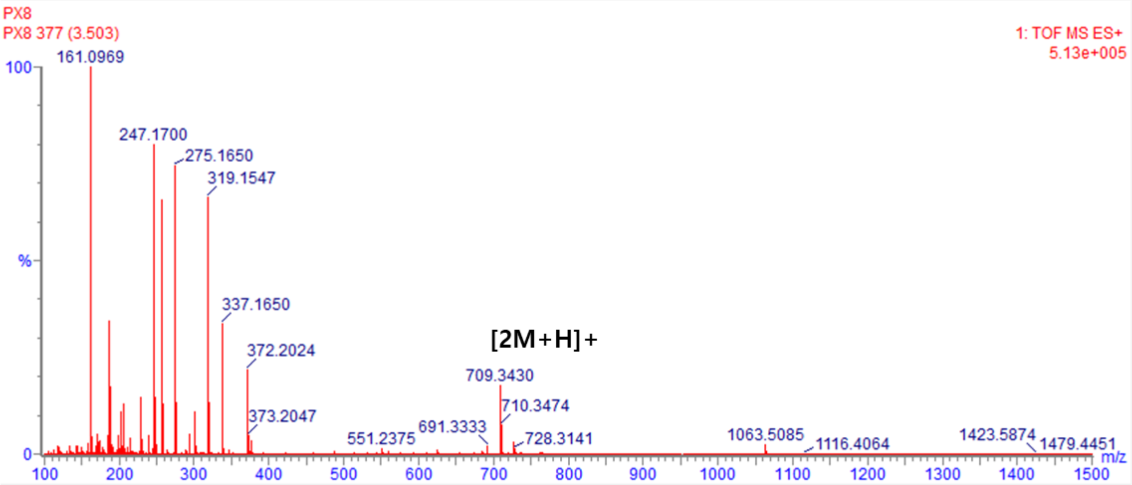

Figure S3. HR-ESIMS data of compound **1**.

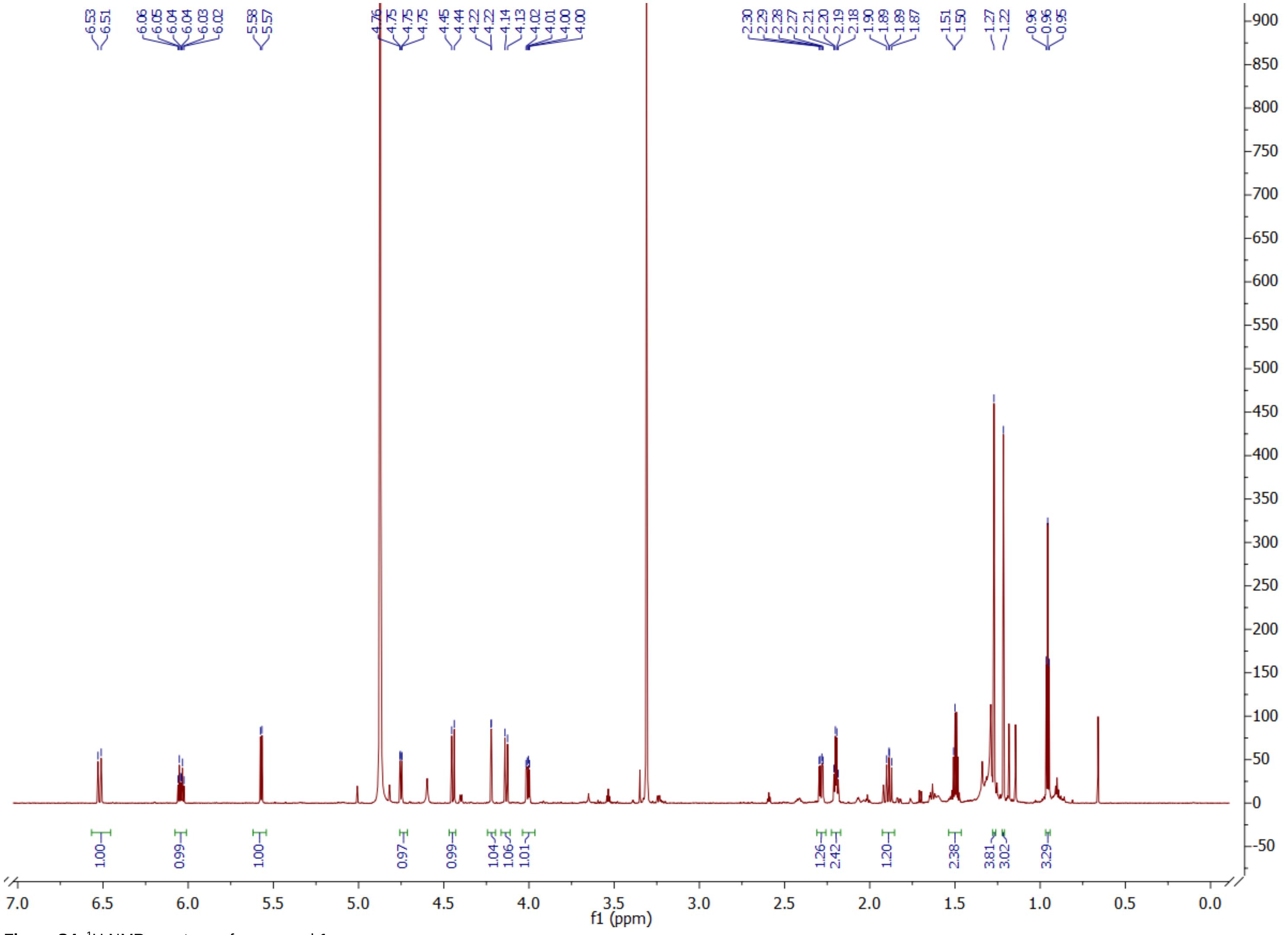

Figure S4. ^{1}H NMR spectrum of compound **1**.

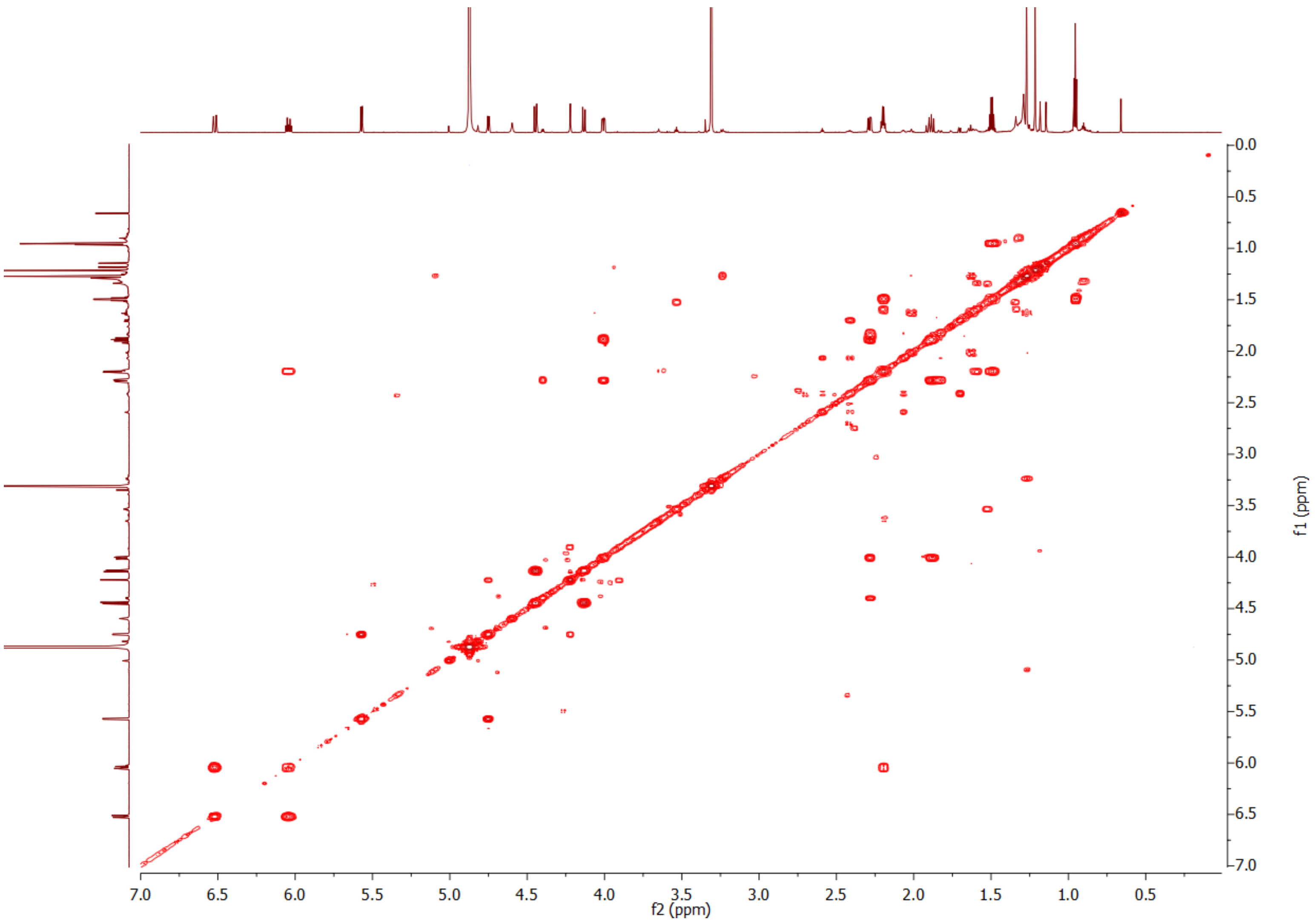

Figure S5. ¹H-¹H COSY spectrum of compound **1**.

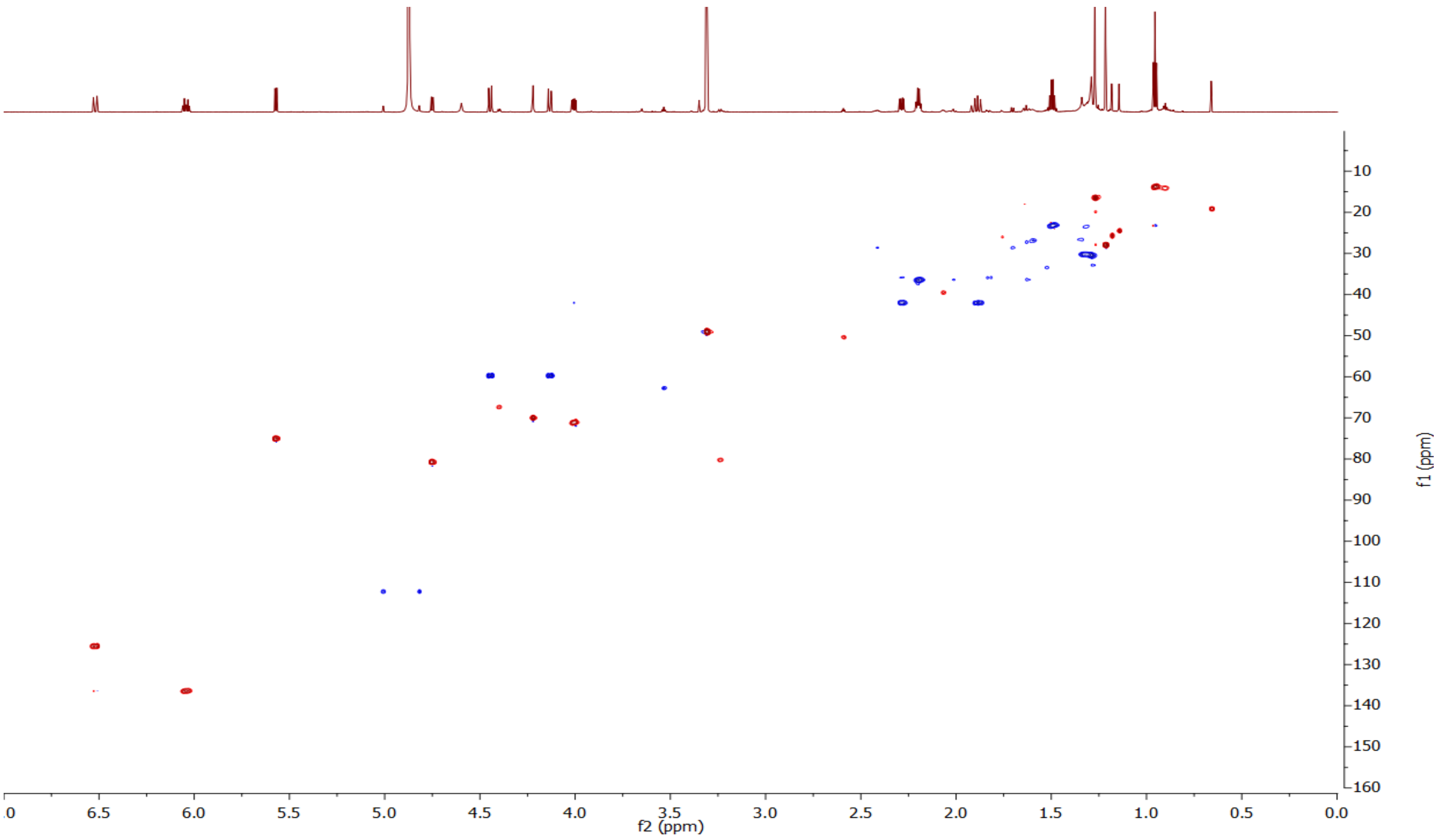

Figure S6. ^{1}H-^{13}C HSQC spectrum of compound **1**.

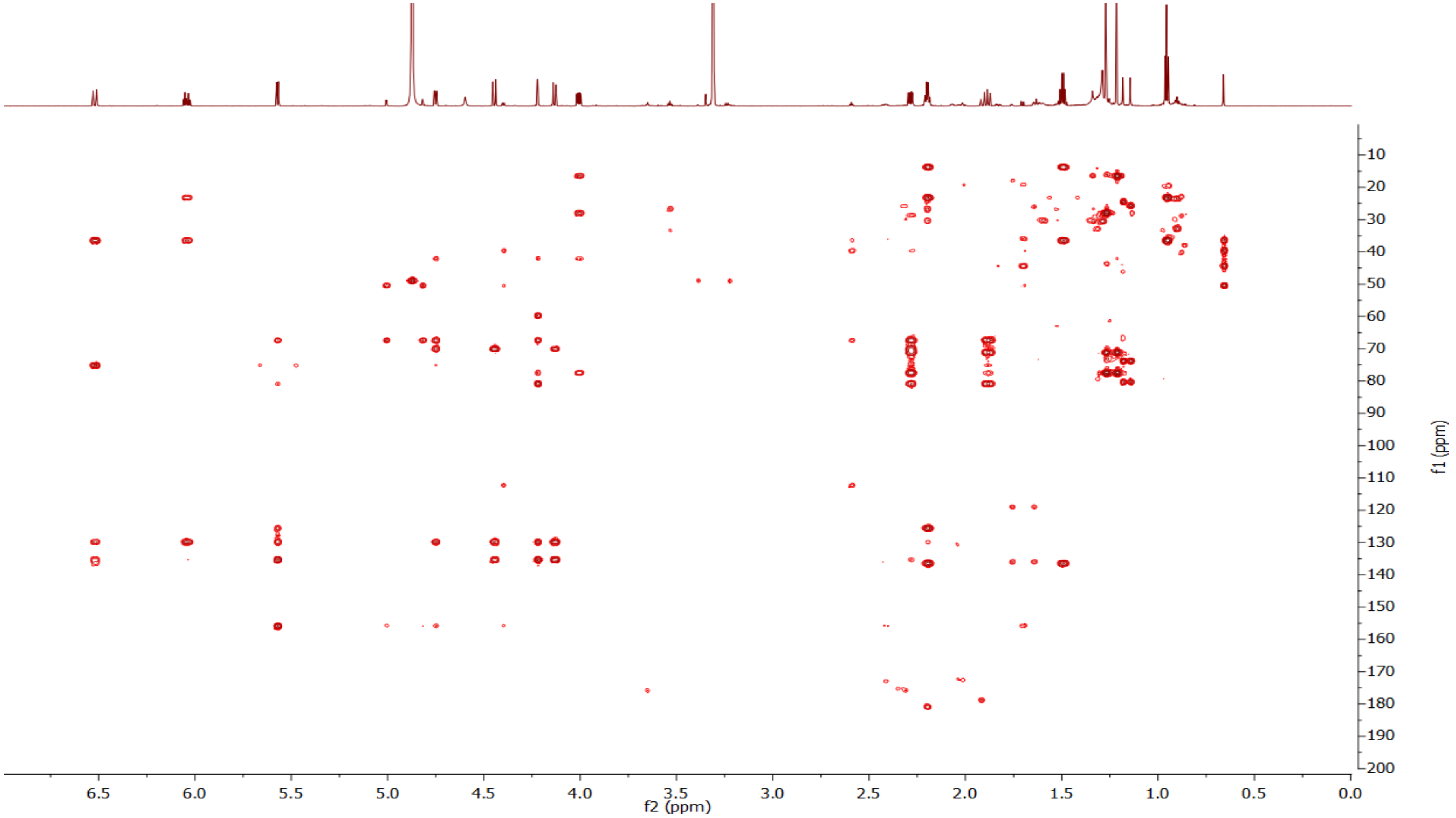

Figure S7. ^{1}H-^{13}C HMBC spectrum of compound **1**.

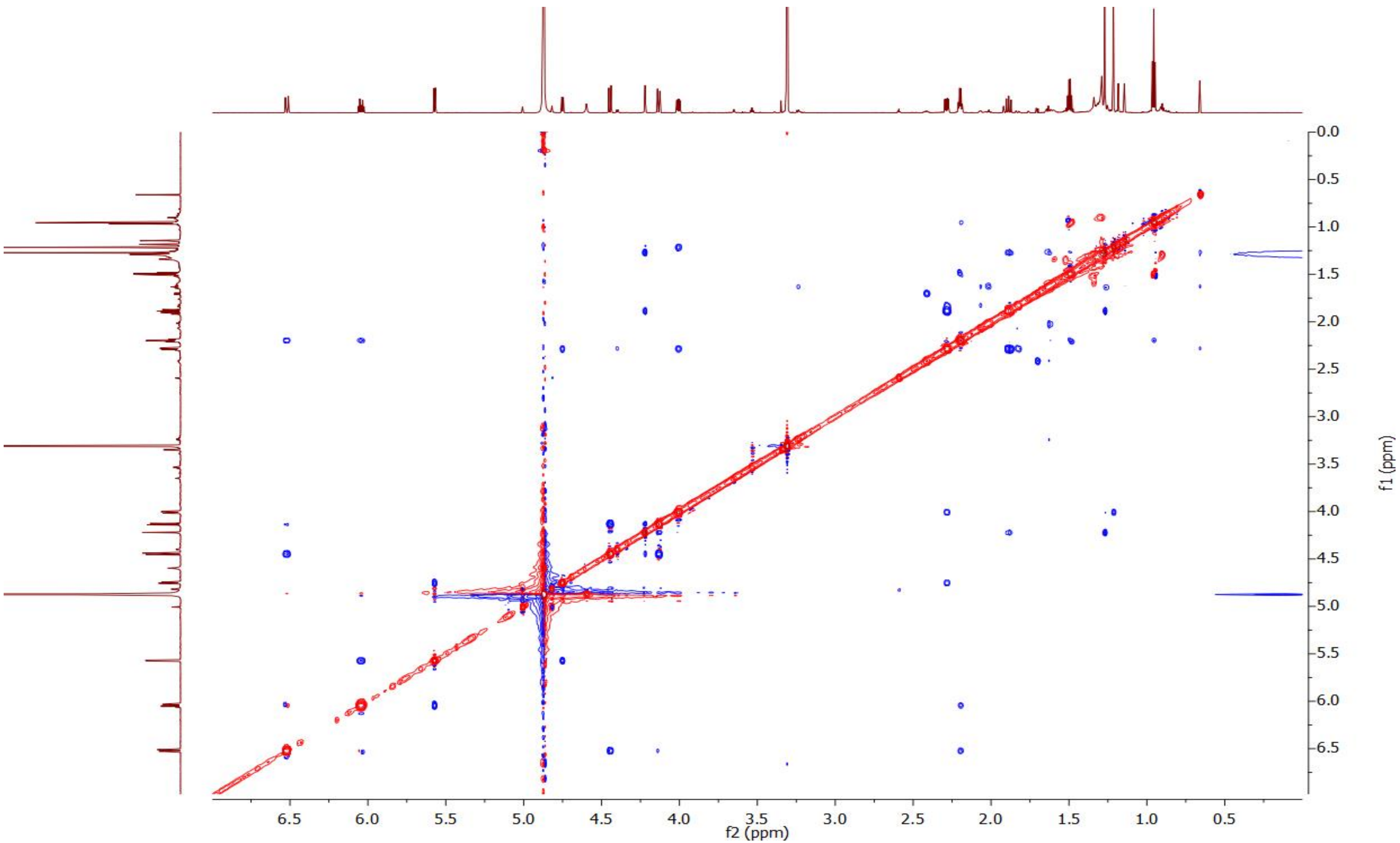

Figure S8. ^1H-^1H ROESY spectrum of compound **1**.

Table S3. Comparison of NMR data of compound **1** and cytosporin E[1] in MeOH-d_4.

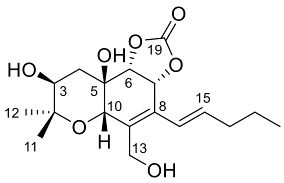

1

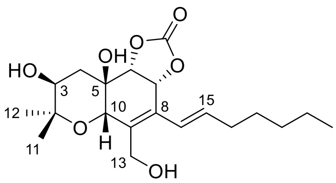

cystoporin E

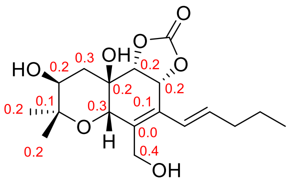

N°	compound **1**		cystoporin E[1]		$\|\Delta\delta_{(\text{compound } \mathbf{1}\text{-cytosporin E})}\|$	
	δ_C	δ_H	δ_C	δ_H	δ_C	δ_H
2	77.5		77.6		0.1	
3	71.2	4.01	71.4	4.05	0.2	0.04
4	42.0	1.89<br>2.29	42.3	1.90<br>2.30	0.3	0.01<br>0.01
5	67.4		67.6		0.2	
6	80.8	4.75	81.0	4.78	0.2	0.03
7	75.1	5.57	75.3	5.57	0.2	0.00
8	130.0		130.1		0.1	
9	135.5		135.5		0.0	
10	70.0	4.22	70.3	4.25	0.3	0.03
11	16.5	1.27	16.8	1.30	0.2	0.03
12	28.0	1.22	28.2	1.25	0.2	0.03
13	59.7	4.13<br>4.45	60.1	4.18<br>4.45	0.4	0.05<br>0.00
				**Average**	**0.17**	**0.02**
				s	**0.10**	**0.02**
				**R^2**	**0.999997**	**0.999941**

Table S4. Comparison of ^{1}H NMR data of compound **1** and calculated ^{1}H NMR data of six possible isomers of **1** in MeOH-d_4.

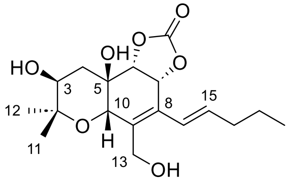

1_1

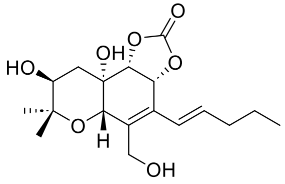

1_2

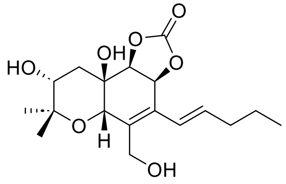

1_3

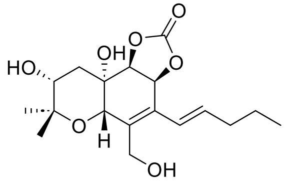

1_4

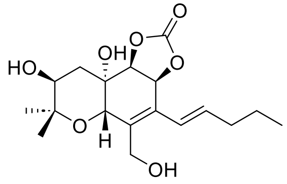

1_5

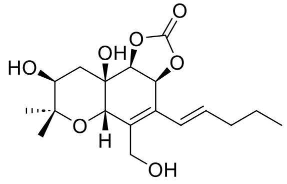

1_6

N°	Exp	1_1				1_2				1_3				1_4				1_5				1_6			
		unscaled	Δ	scaled	Δ	unscaled	Δ	scaled	Δ	unscaled	Δ	scaled	Δ	unscaled	Δ	scaled	Δ	unscaled	Δ	scaled	Δ	unscaled	Δ	scaled	Δ
3	**4,01**	4,31	0,30	4,13	0,12	4,02	0,01	3,94	0,07	3,91	0,10	3,71	0,30	3,56	0,45	3,55	0,46	4,12	0,11	4,15	0,14	3,82	0,19	3,74	0,27
4	**1,89**	1,98	0,09	1,89	0,00	1,76	0,13	1,79	0,10	1,99	0,10	1,93	0,04	1,99	0,10	1,92	0,03	1,63	0,26	1,58	0,31	1,48	0,41	1,53	0,36
	**2,29**	2,16	0,13	2,06	0,23	2,03	0,26	2,04	0,25	2,14	0,15	2,07	0,22	2,39	0,10	2,33	0,04	2,20	0,09	2,17	0,12	2,32	0,03	2,33	0,04
6	**4,75**	4,86	0,11	4,67	0,08	4,60	0,15	4,49	0,26	6,07	1,32	5,71	0,96	4,69	0,06	4,74	0,01	4,54	0,21	4,59	0,16	5,00	0,25	4,84	0,09
7	**5,57**	5,51	0,06	5,29	0,28	5,48	0,09	5,34	0,23	5,51	0,06	5,19	0,38	5,13	0,44	5,20	0,37	5,18	0,39	5,25	0,32	5,55	0,02	5,37	0,20
10	**4,22**	4,48	0,26	4,29	0,07	4,41	0,19	4,31	0,09	4,35	0,13	4,11	0,11	4,94	0,72	5,00	0,78	4,76	0,54	4,82	0,60	4,18	0,04	4,08	0,14
11	**1,27**	1,30	0,03	1,23	0,04	1,21	0,06	1,26	0,01	1,32	0,05	1,31	0,04	1,28	0,01	1,18	0,09	1,26	0,01	1,20	0,07	1,25	0,02	1,31	0,04
12	**1,22**	1,50	0,28	1,42	0,20	1,35	0,13	1,39	0,17	1,23	0,01	1,22	0,00	1,35	0,13	1,25	0,03	1,39	0,17	1,34	0,12	1,22	0,00	1,28	0,06
13	**4,13**	4,30	0,17	4,12	0,01	4,51	0,38	4,41	0,28	4,45	0,32	4,21	0,08	4,29	0,16	4,32	0,19	4,27	0,14	4,31	0,18	4,34	0,21	4,22	0,09
	**4,45**	4,72	0,27	4,53	0,08	4,58	0,13	4,48	0,03	4,46	0,01	4,22	0,23	4,57	0,12	4,61	0,16	4,56	0,11	4,61	0,16	4,60	0,15	4,46	0,01
14	**6,52**	6,76	0,24	6,49	0,03	6,78	0,26	6,58	0,06	6,76	0,24	6,35	0,17	6,09	0,43	6,21	0,31	6,11	0,41	6,21	0,31	6,85	0,33	6,58	0,06
15	**6,04**	6,51	0,47	6,25	0,21	6,41	0,37	6,23	0,19	6,66	0,62	6,26	0,22	6,17	0,13	6,28	0,24	6,08	0,04	6,18	0,14	6,52	0,48	6,28	0,24
16	**2,2**	2,37	0,17	2,26	0,06	2,26	0,06	2,26	0,06	2,32	0,12	2,24	0,04	2,20	0,00	2,14	0,06	2,24	0,04	2,22	0,02	2,35	0,15	2,35	0,15
17	**1,5**	1,58	0,08	1,50	0,00	1,51	0,01	1,54	0,04	1,56	0,06	1,53	0,03	1,49	0,01	1,40	0,10	1,53	0,03	1,48	0,02	1,60	0,10	1,64	0,14
18	**0,96**	0,93	0,03	0,87	0,09	0,91	0,05	0,97	0,01	0,95	0,01	0,97	0,01	1,01	0,05	0,89	0,07	0,98	0,02	0,91	0,05	0,92	0,04	1,00	0,04
**R^2**		**0,99734**		**0,99734**		**0,99642**		**0,99642**		**0,98665**		**0,98666**		**0,98808**		**0,98808**		**0,99198**		**0,99198**		**0,99604**		**0,99604**	
**MAE**			**0,18**		**0,10**		**0,15**		**0,12**		**0,22**		**0,19**		**0,19**		**0,20**		**0,17**		**0,18**		**0,16**		**0,13**

Table S5. Comparison of ^{13}C NMR data of compound **1** and calculated ^{13}C NMR data of 6 possible isomers of **1** in MeOH-d_4.

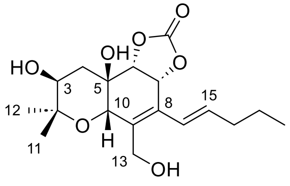

1_1

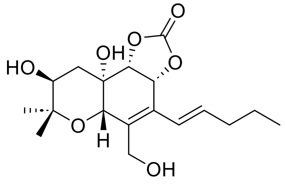

1_2

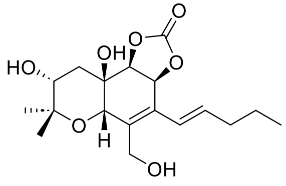

1_3

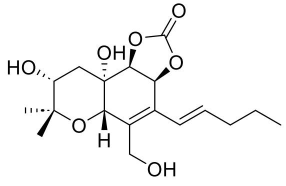

1_4

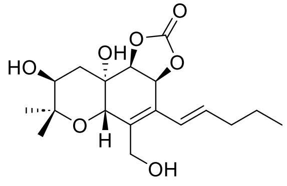

1_5

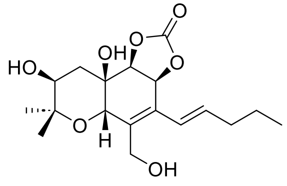

1_6

N°	Exp	1_1				1_2				1_3				1_4				1_5				1_6			
		unscaled	Δ	scaled	Δ	unscaled	Δ	scaled	Δ	unscaled	Δ	scaled	Δ	unscaled	Δ	scaled	Δ	unscaled	Δ	scaled	Δ	unscaled	Δ	scaled	Δ
2	**77,5**	81,0	3,5	79,5	2,0	81,3	3,8	80,1	2,6	80,8	3,3	78,9	1,4	82,1	4,6	79,9	2,4	81,0	3,5	78,8	1,3	80,5	3,0	79,0	1,5
3	**71,2**	74,1	2,9	72,4	1,2	73,7	2,5	72,3	1,1	75,0	3,8	73,0	1,8	74,9	3,7	72,6	1,4	73,4	2,2	71,2	0,0	74,9	3,7	73,3	2,1
4	**42**	43,7	1,7	41,5	0,5	40,8	1,2	38,8	3,2	38,4	3,6	35,6	6,4	36,1	5,9	33,4	8,6	42,1	0,1	39,6	2,4	41,9	0,1	39,7	2,3
5	**67,4**	70,7	3,3	69,0	1,6	75,6	8,2	74,2	6,8	70,9	3,5	68,8	1,4	74,8	7,4	72,5	5,1	76,0	8,6	73,8	6,4	73,1	5,7	71,5	4,1
6	**80,8**	83,3	2,5	81,8	1,0	81,7	0,9	80,4	0,4	80,9	0,1	79,0	1,8	80,8	0,0	78,5	2,3	81,8	1,0	79,6	1,2	78,6	2,2	77,1	3,7
7	**75,1**	76,7	1,6	75,1	0,0	77,1	2,0	75,8	0,7	76,9	1,8	74,9	0,2	79,5	4,4	77,3	2,2	81,4	6,3	79,2	4,1	76,9	1,8	75,3	0,2
8	**130**	133,8	3,8	133,2	3,2	129,2	0,8	129,0	1,0	135,1	5,1	134,3	4,3	135,6	5,6	134,0	4,0	134,1	4,1	132,3	2,3	132,8	2,8	132,4	2,4
9	**135,5**	134,4	1,1	133,9	1,6	139,3	3,8	139,2	3,7	133,9	1,6	133,0	2,5	140,6	5,1	139,0	3,5	140,8	5,3	139,0	3,5	134,1	1,4	133,6	1,9
10	**70**	72,1	2,1	70,4	0,4	71,8	1,8	70,4	0,4	76,3	6,3	74,3	4,3	70,7	0,7	68,4	1,6	70,6	0,6	68,4	1,6	75,7	5,7	74,1	4,1
11	**16,5**	16,5	0,0	13,8	2,7	15,9	0,6	13,3	3,2	23,2	6,7	20,2	3,7	22,0	5,5	19,2	2,7	16,0	0,5	13,4	3,1	16,5	0,0	13,8	2,7
12	**28**	28,7	0,7	26,3	1,7	28,7	0,7	26,4	1,6	26,2	1,8	23,2	4,8	26,9	1,1	24,1	3,9	29,1	1,1	26,5	1,5	28,8	0,8	26,3	1,7
13	**59,7**	61,6	1,9	59,8	0,1	58,6	1,1	56,9	2,8	62,8	3,1	60,5	0,8	60,8	1,1	58,4	1,3	61,4	1,7	59,1	0,6	61,5	1,8	59,7	0,0
14	**125,5**	122,7	2,8	121,9	3,6	121,6	3,9	121,2	4,3	122,6	2,9	121,5	4,0	123,1	2,4	121,3	4,2	123,7	1,8	121,7	3,8	122,7	2,8	122,0	3,5
15	**136,4**	140,5	4,1	140,1	3,7	137,4	1,0	137,3	0,9	140,5	4,1	139,8	3,4	139,7	3,3	138,1	1,7	138,5	2,1	136,7	0,3	139,2	2,8	138,8	2,4
16	**36,5**	40,1	3,6	37,8	1,3	40,8	4,3	38,8	2,3	40,8	4,3	38,1	1,6	40,9	4,4	38,2	1,7	39,7	3,2	37,3	0,8	40,4	3,9	38,2	1,7
17	**23,2**	27,3	4,1	24,8	1,6	27,5	4,3	25,2	2,0	27,5	4,3	24,5	1,3	28,7	5,5	26,0	2,8	27,1	3,9	24,5	1,3	26,8	3,6	24,3	1,1
18	**13,8**	14,8	1,0	12,1	1,7	15,4	1,6	12,8	1,0	15,5	1,7	12,3	1,5	16,2	2,4	13,4	0,4	15,7	1,9	13,1	0,7	15,0	1,2	12,3	1,5
19	**155,9**	152,0	3,9	151,7	4,2	152,9	3,0	153,1	2,8	153,7	2,2	153,2	2,7	152,2	3,7	150,7	5,2	152,6	3,3	150,8	5,1	153,7	2,2	153,6	2,3
**R^2**		0,99879		0,99879		0,99801		0,99801		0,99753		0,99753		0,99668		0,99668		0,99795		0,99795		0,99843		0,99843	
**MAE**			2,47		1,78		2,52		2,26		3,35		2,64		3,72		3,06		2,85		2,22		2,53		2,18

Functional	Solvent?	Basis Set	Type of Data
B3LYP	PCM	6-31+G(d,p)	Unscaled Shifts

	Isomer 1	Isomer 2	Isomer 3	Isomer 4	Isomer 5	Isomer 6
sDP4+ (H data)	95,23%	3,39%	0,00%	0,00%	0,00%	1,38%
sDP4+ (C data)	98,41%	0,21%	0,00%	0,00%	0,23%	1,15%
sDP4+ (all data)	99,98%	0,01%	0,00%	0,00%	0,00%	0,02%
uDP4+ (H data)	1,42%	77,70%	0,80%	0,01%	0,01%	20,08%
uDP4+ (C data)	87,78%	0,20%	0,83%	0,00%	0,02%	11,17%
uDP4+ (all data)	34,12%	4,17%	0,18%	0,00%	0,00%	61,53%
DP4+ (H data)	31,66%	61,84%	0,00%	0,00%	0,00%	6,50%
DP4+ (C data)	99,85%	0,00%	0,00%	0,00%	0,00%	0,15%
DP4+ (all data)	99,97%	0,00%	0,00%	0,00%	0,00%	0,03%

Figure S9. DP4+ probability analysis for the determination of the absolute configuration of **1**.

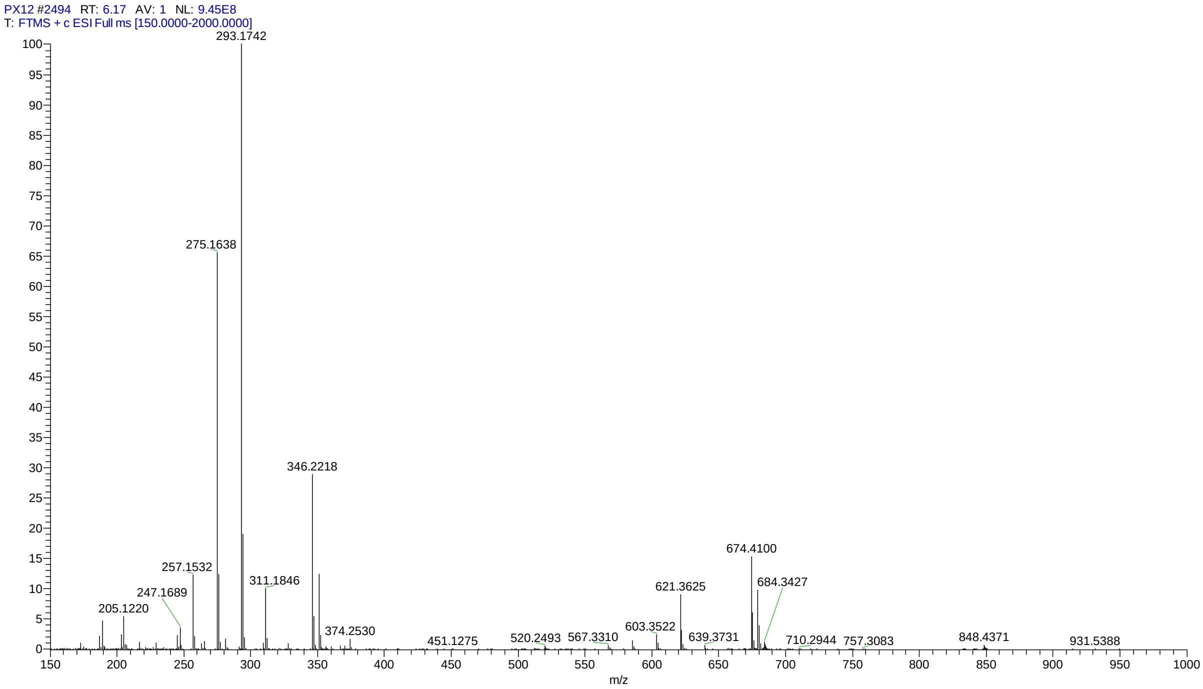

Figure S10. HR-ESIMS data of compound **2**.

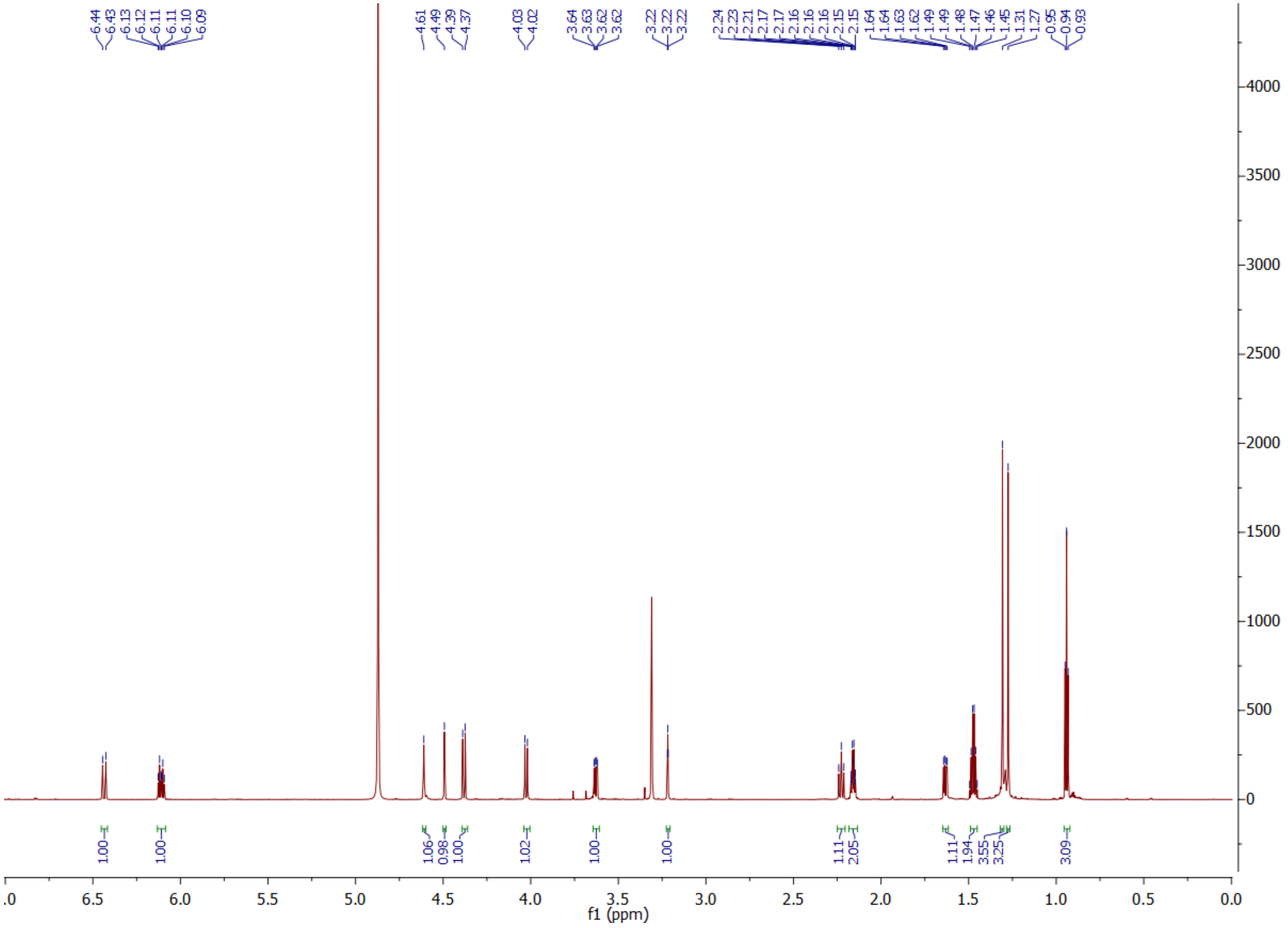

Figure S11. ^{1}H NMR spectrum of compound **2**.

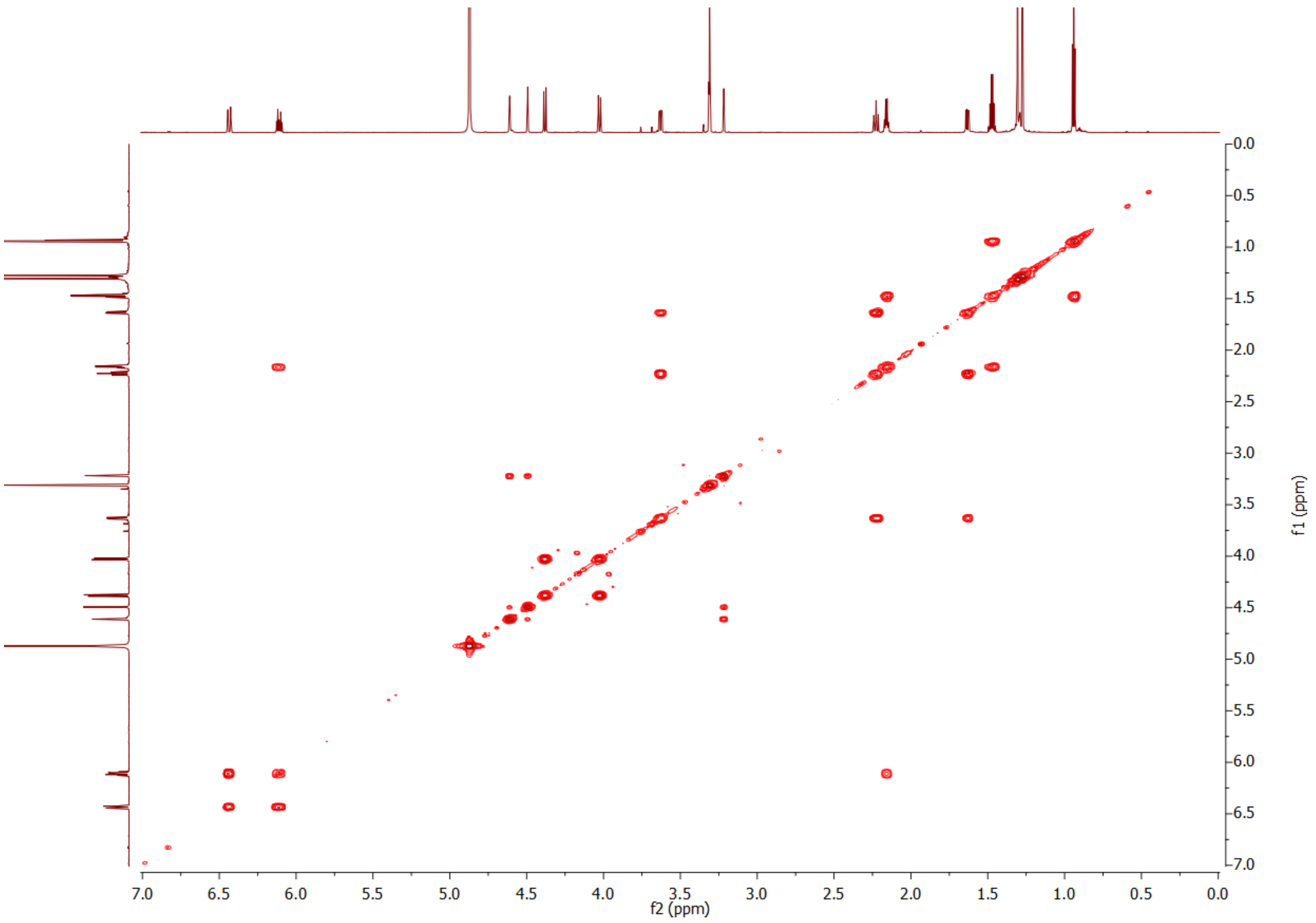

Figure S12. ^{1}H-^{1}H COSY spectrum of compound **2**.

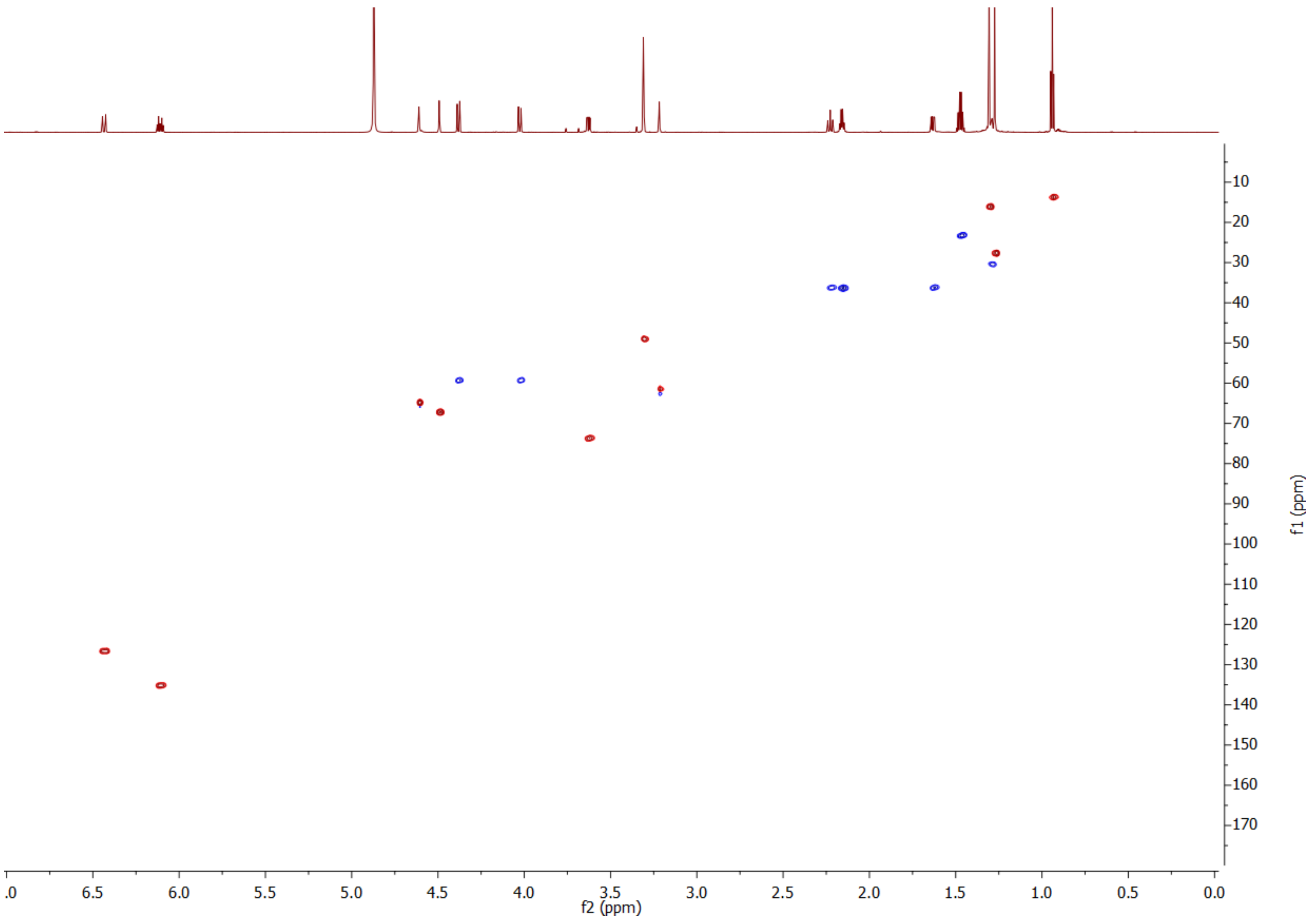

Figure S13. ^{1}H-^{13}C HSQC spectrum of compound **2**.

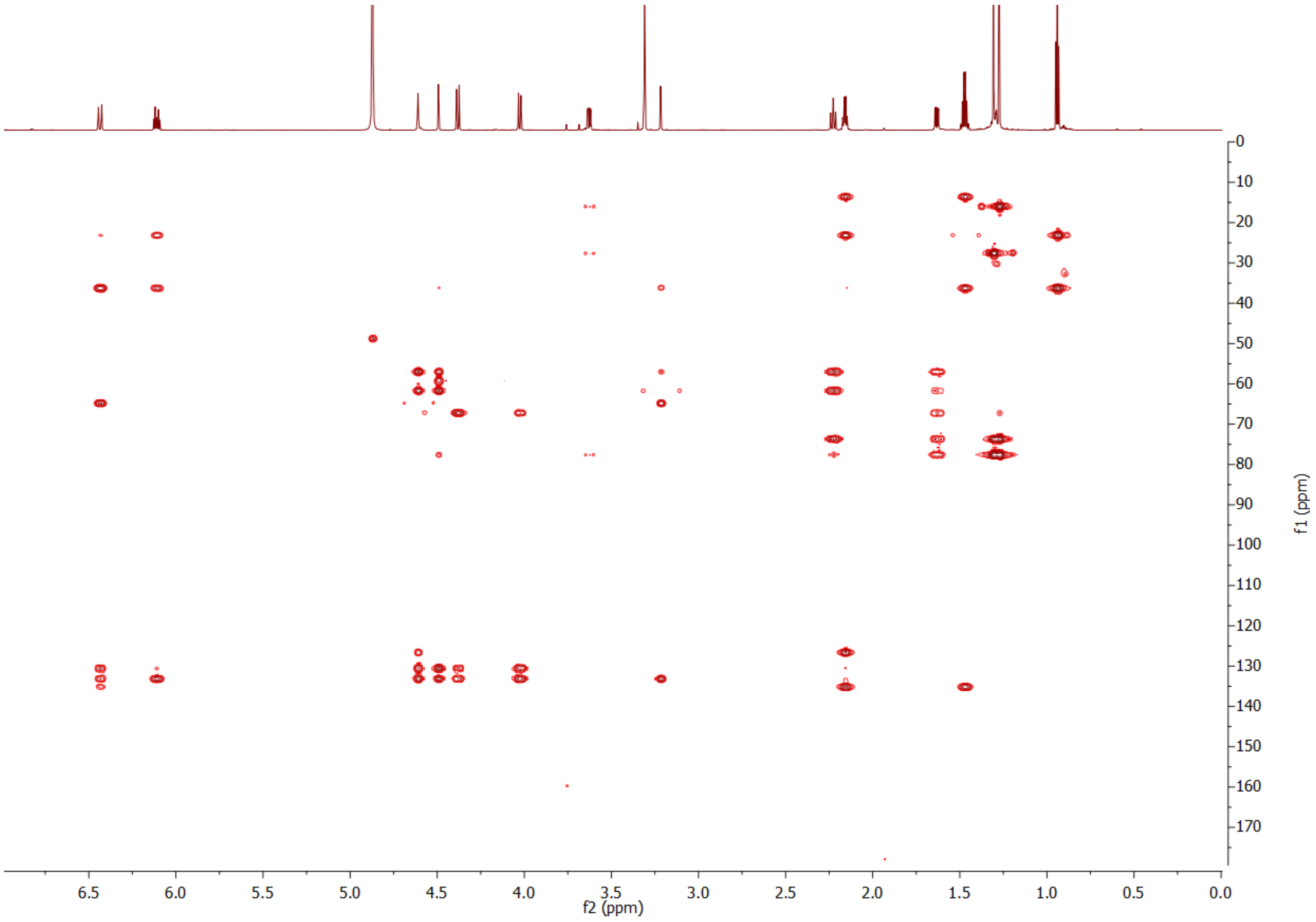

Figure S14. ^{1}H-^{13}C HMBC spectrum of compound **2**.

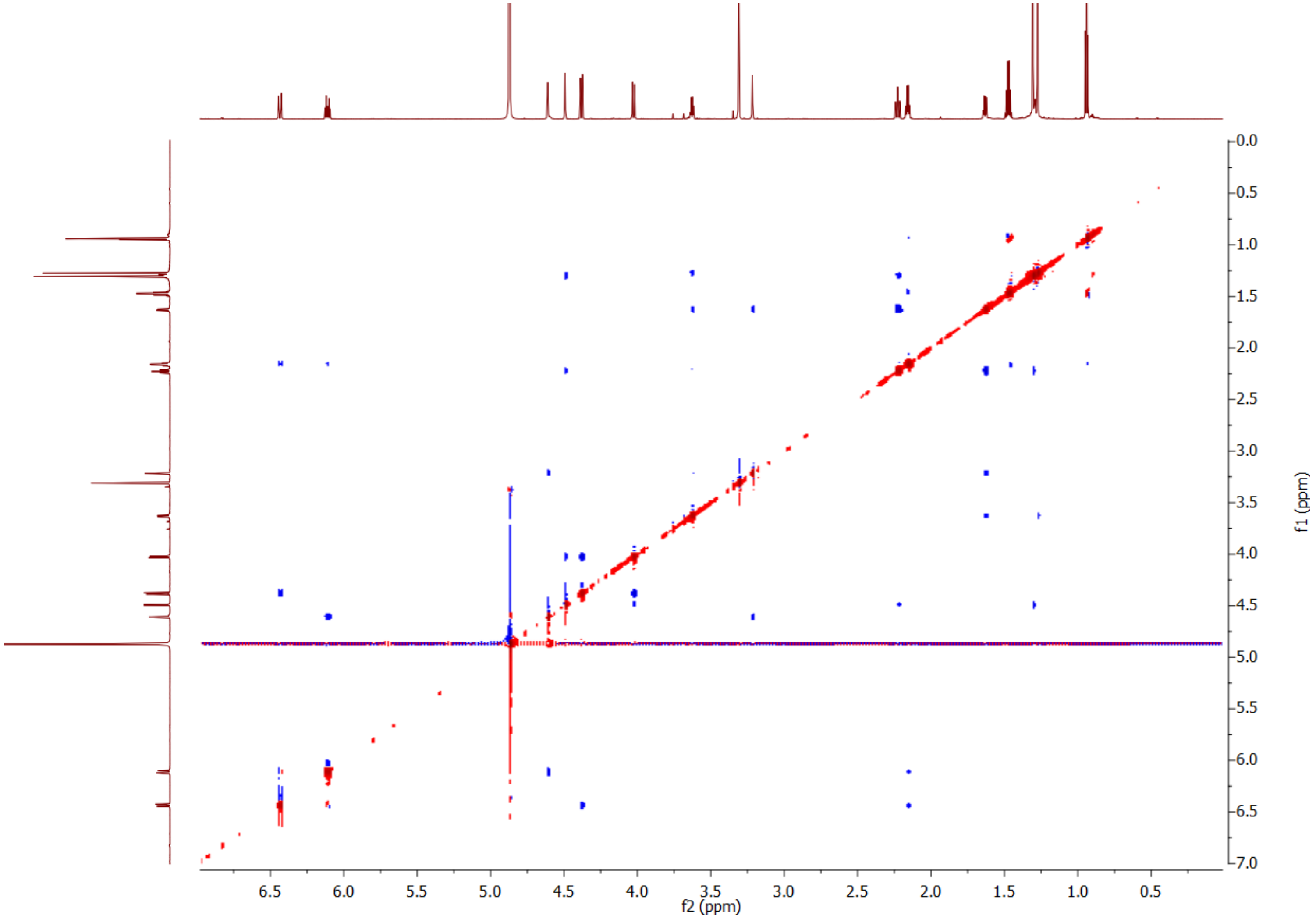

Figure S15. ^1H-^1H ROESY spectrum of compound **2**.

Single Mass Analysis
Tolerance = 5.0 mDa / DBE: min = -1.5, max = 50.0
Element prediction: Off
Number of isotope peaks used for i-FIT = 3
Monoisotopic Mass, Even Electron Ions
183 formula(e) evaluated with 2 results within limits (up to 50 closest results for each mass)
Elements Used:

Mass	Calc. Mass	mDa	PPM	DBE	Formula	i-FIT	i-FIT Norm	Fit Conf %	C	H	O
621.3627	621.3639	-1.2	-1.9	8.5	C34 H53 O10	201.5	0.147	86.30	34	53	10
	621.3580	4.7	7.6	17.5	C41 H49 O5	203.4	1.988	13.70	41	49	5

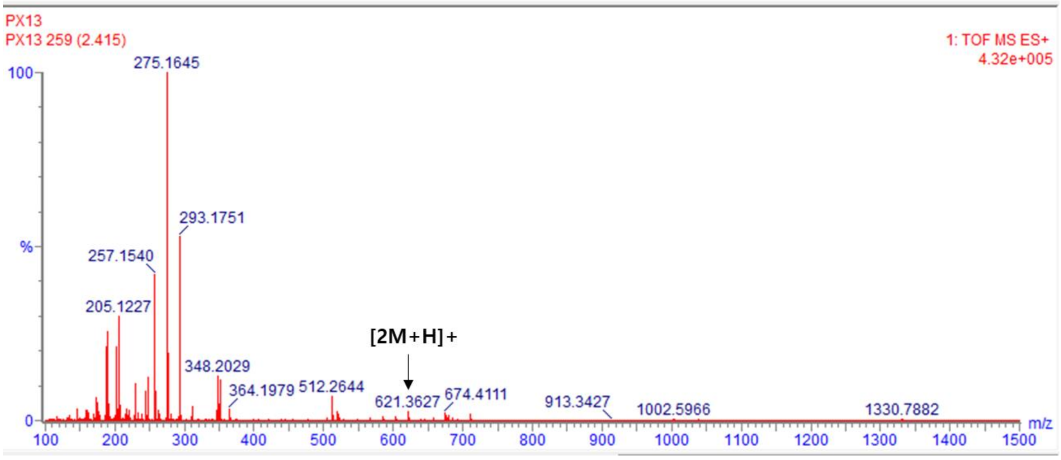

Figure S16. HR-ESIMS data of compound **3**.

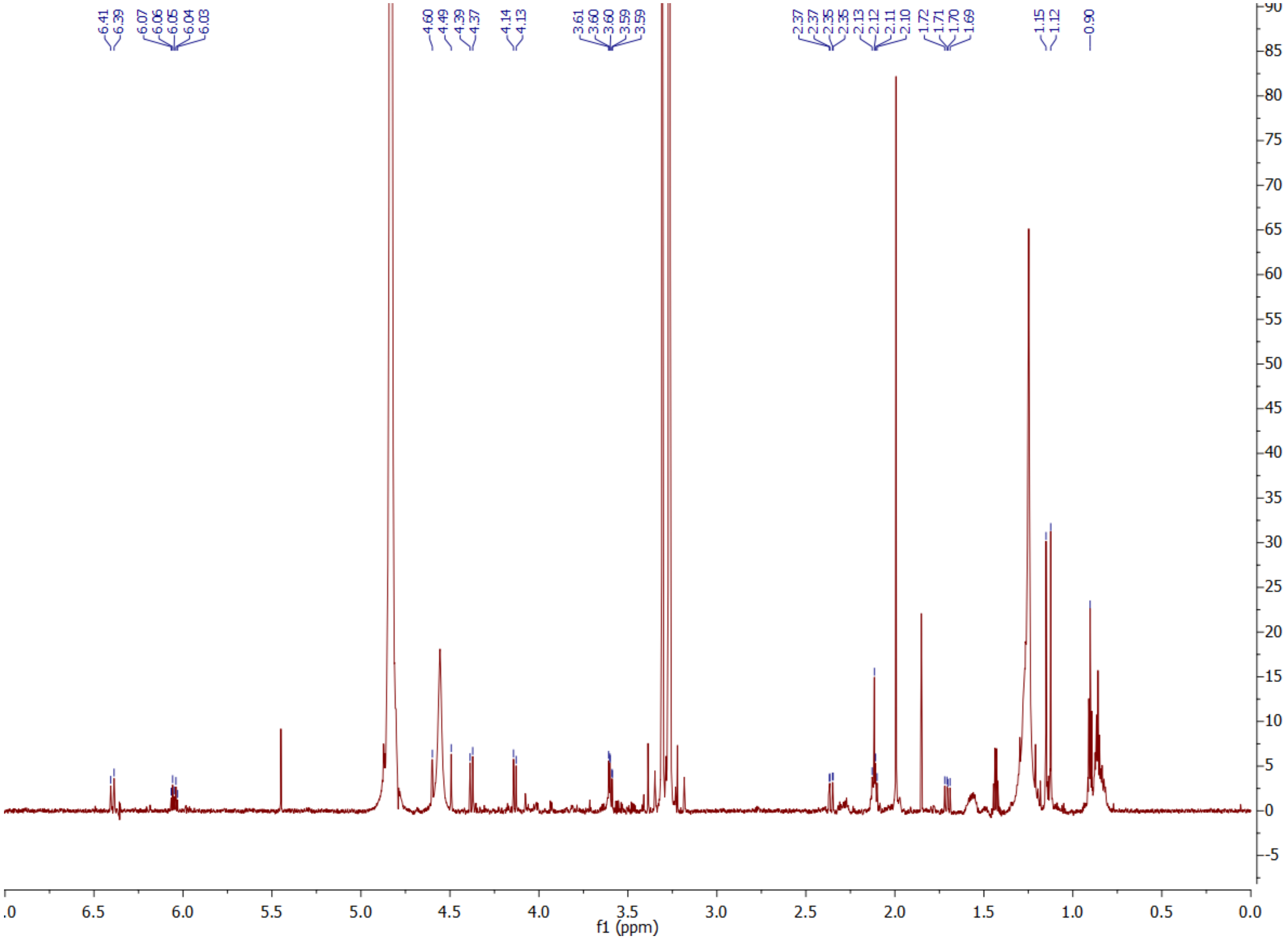

Figure S17. ^{1}H NMR spectrum of compound **3**.

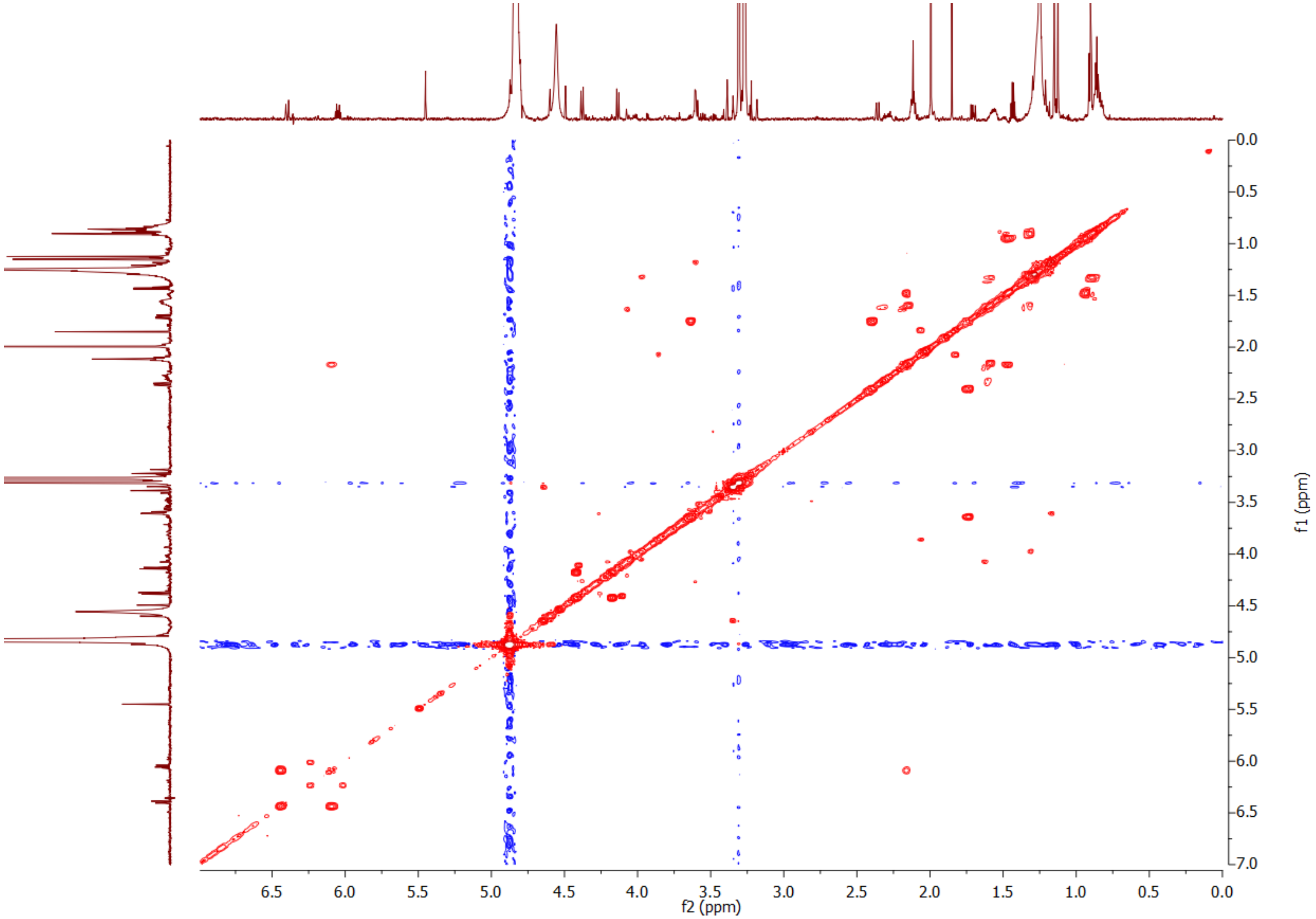

Figure S18. ^{1}H-^{1}H COSY spectrum of compound **3**.

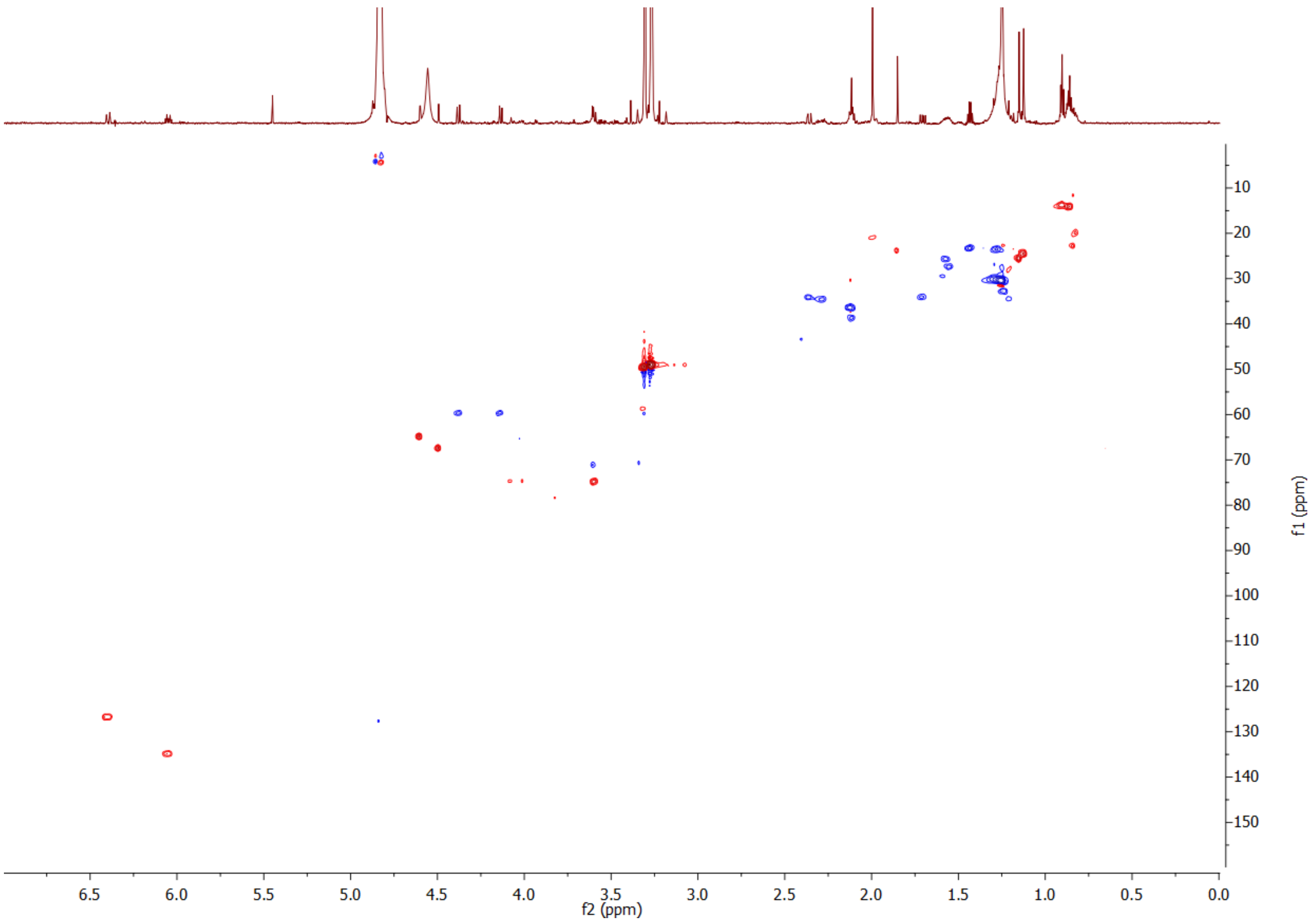

Figure S19. ^{1}H-^{13}C HSQC spectrum of compound **3**.

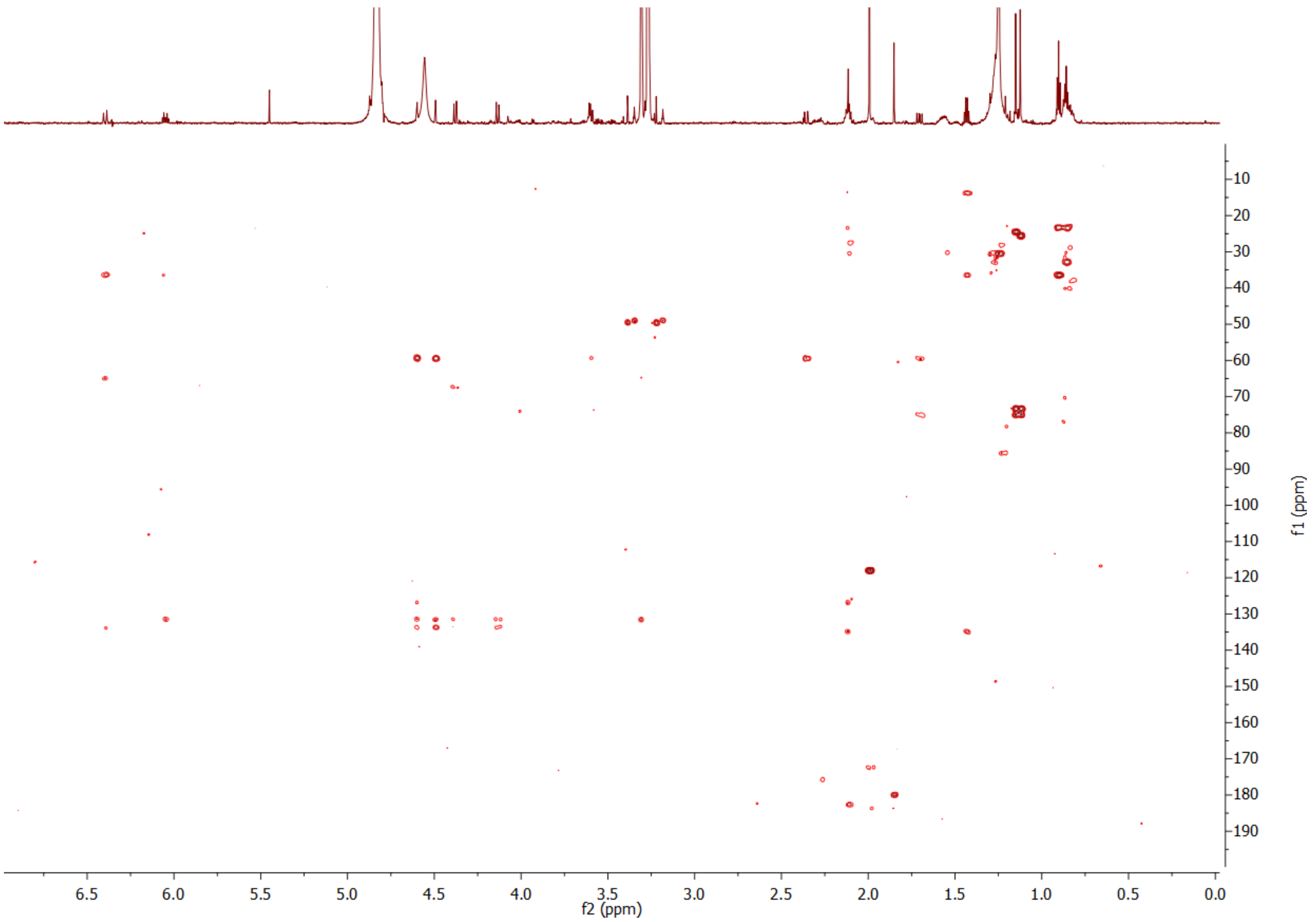

Figure S20. ^{1}H-^{13}C HMBC spectrum of compound **3**.

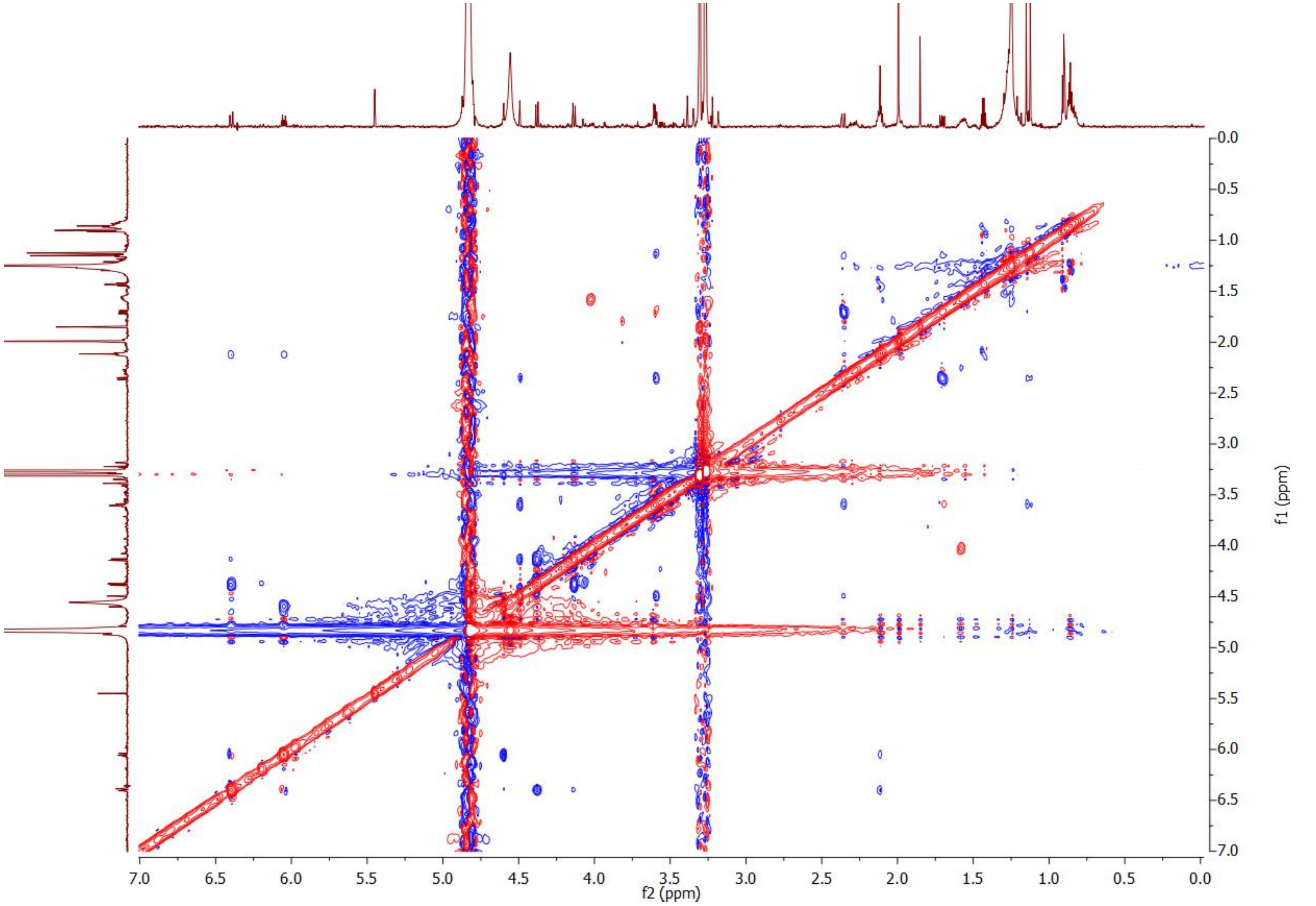

Figure S21. ^{1}H-^{1}H ROESY spectrum of compound **3**.

Table S6. Comparison of NMR data of compound **2** and cytosporin D[1] in MeOH-d_4.

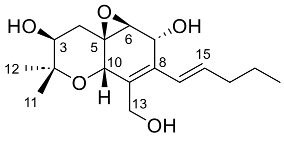

2

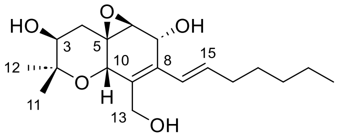

cytosporin D

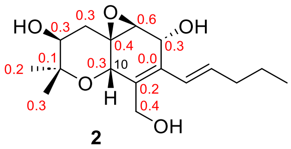

2

N°	compound **2**		cystoporin D[1]		$\lvert\Delta\delta_{(\text{compound } \mathbf{2}\text{-cytosporin D})}\rvert$	
	δ_C	δ_H	δ_C	δ_H	δ_C	δ_H
2	77.6		77.7		0.1	
3	73.7	3.63	74.0	3.68	0.3	0.05
4	36.2	1.63<br>2.23	36.5	1.70<br>2.32	0.3	0.07<br>0.09
5	57.0		57.4		0.4	
6	61.5	3.22	62.1	3.29	0.6	0.07
7	64.9	4.61	65.2	4.65	0.3	0.04
8	130.7		130.7		0.0	
9	133.3		133.5		0.2	
10	67.3	4.49	67.6	4.53	0.3	0.04
11	16.1	1.31	16.4	1.30	0.3	0.01
12	27.7	1.27	27.9	1.25	0.2	0.02
13	59.3	4.03<br>4.38	59.7	4.08<br>4.31	0.4	0.05<br>0.07
				**Average**	**0.24**	**0.04**
				s	**0.15**	**0.05**
				**R^2**	**0.999993**	**0.999336**

Table S7. Comparison of NMR data of compound **2** and cytosporin M[2] in MeOH-d_4.

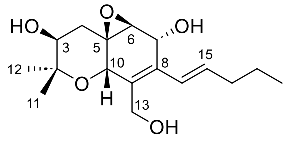

2

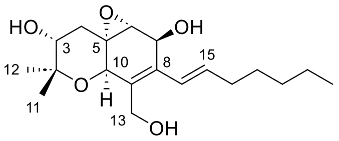

cystoporin M

N°	compound **2**		cystoporin M[2]		|Δδ(compound **2**-cytosporin M)|	
	δ_C	δ_H	δ_C	δ_H	δ_C	δ_H
2	77.6		77.3		0.3	
3	73.7	3.63	74.0	3.56	0.3	0.07
4	36.2	1.63<br>2.23	36.7	1.64<br>2.21	0.5	0.01<br>0.02
5	57.0		59.8		**2.8**	
6	61.5	3.22	63.4	3.36	1.9	0.14
7	64.9	4.61	67.1	4.61	**2.2**	0.00
8	130.7		130.8		0.1	
9	133.3		135.6		**2.3**	
10	67.3	4.49	67.4	4.47	0.1	0.02
11	16.1	1.31	16.4	1.30	0.3	0.01
12	27.7	1.27	28.0	1.24	0.3	0.03
13	59.3	4.03<br>4.38	60.1	4.02<br>4.30	0.8	0.01<br>0.08
				**Average**	**0.85**	**0.03**
				s	**1.01**	**0.06**
				**R^2**	**0.999576**	**0.999031**

Table S8. Comparison of NMR data of compound **3** and cytosporin D[1] in MeOH-d_4.

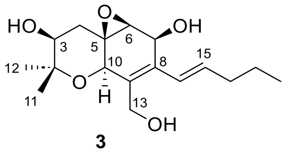

3

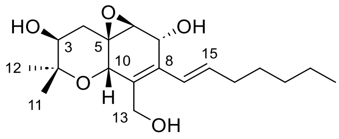

cytosporin D

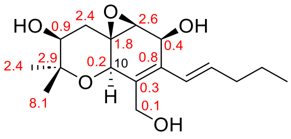

3

N°	compound **3**		cystoporin D[1]		$\lvert\Delta\delta_{(\text{compound } \mathbf{3}\text{-cytosporin D})}\rvert$	
	δ_C	δ_H	δ_C	δ_H	δ_C	δ_H
2	74.8		77.7		2.9	
3	73.1	3.59	74.0	3.68	0.9	0.09
4	34.1	1.70<br>2.36	36.5	1.70<br>2.32	2.4	0.00<br>0.04
5	59.2		57.4		1.8	
6	59.5	3.29	62.1	3.29	2.6	0.00
7	64.8	4.60	65.2	4.65	0.4	0.05
8	131.5		130.7		0.8	
9	133.8		133.5		0.3	
10	67.4	4.49	67.6	4.53	0.2	0.04
11	24.5	1.15	16.4	1.30	**8.1**	0.15
12	25.5	1.12	27.9	1.25	2.4	0.13
13	59.6	4.14<br>4.38	59.7	4.08<br>4.31	0.1	0.06<br>0.07
				**Average**	**1.64**	**0.05**
				s	**2.85**	**0.07**
				**R^2**	**0.996515**	**0.998805**

Table S9. Comparison of NMR data of compound **3** and cytosporin M[2] in MeOH-d_4.

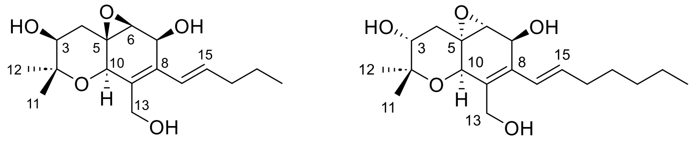

3 cystoporin M

N°	compound **3**		cystoporin M[2]		|Δδ(compound **3**-cytosporin M)|	
	δ_C	δ_H	δ_C	δ_H	δ_C	δ_H
2	74.80		77.30		2.5	
3	73.10	3.59	74.00	3.56	0.9	0.03
4	34.10	1.70<br>2.36	36.70	1.64<br>2.21	**2.6**	0.06<br>0.15
5	59.20		59.80		0.6	
6	59.50	3.29	63.40	3.36	**3.9**	0.07
7	64.80	4.60	67.10	4.61	**2.3**	0.01
8	131.50		130.80		0.7	
9	133.80		135.60		1.8	
10	67.40	4.49	67.40	4.47	0.0	0.02
11	24.50	1.15	16.40	1.30	**8.1**	0.15
12	25.50	1.12	28.00	1.24	2.5	0.12
13	59.60	4.14<br>4.38	60.10	4.02<br>4.30	0.50	0.12<br>0.08
				**Average**	**1.89**	**0.06**
				s	**2.94**	**0.09**
				**R^2**	**0.996401**	**0.997866**

Table S10. Comparison of ^{1}H NMR data of compound **2** and calculated ^{1}H NMR data of 6 possible isomers of **2** in MeOH-d_4.

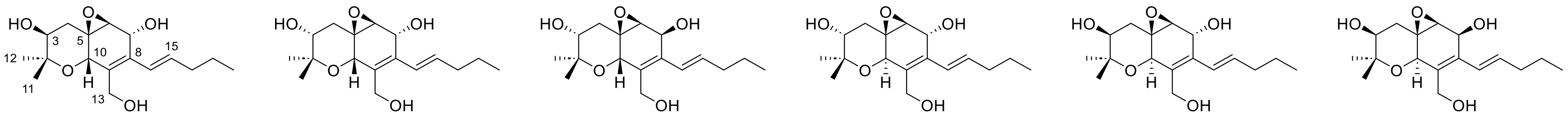

2_1 **2_2** **2_3** **2_4** **2_5** **2_6**

N°	Exp	2_1				2_2				2_3				2_4				2_5				2_6			
		unscaled	Δ	scaled	Δ	unscaled	Δ	scaled	Δ	unscaled	Δ	scaled	Δ	unscaled	Δ	scaled	Δ	unscaled	Δ	scaled	Δ	unscaled	Δ	scaled	Δ
3	**3,63**	3,85	0,22	3,68	0,05	3,99	0,36	3,76	0,13	3,96	0,33	3,82	0,19	3,96	0,33	3,74	0,11	3,81	0,18	3,68	0,05	3,79	0,16	3,71	0,08
4	**1,63**	1,56	0,07	1,58	0,05	1,32	0,31	1,31	0,32	1,32	0,31	1,29	0,34	1,56	0,07	1,53	0,10	1,57	0,06	1,53	0,10	1,53	0,10	1,49	0,14
	**2,23**	2,25	0,02	2,22	0,01	2,60	0,37	2,49	0,26	2,62	0,39	2,53	0,30	2,34	0,11	2,24	0,01	2,65	0,42	2,56	0,33	2,62	0,39	2,57	0,34
6	**3,22**	3,20	0,02	3,09	0,13	3,23	0,01	3,07	0,15	3,40	0,18	3,28	0,06	3,15	0,07	2,99	0,23	3,03	0,19	2,93	0,29	3,23	0,01	3,16	0,06
7	**4,61**	4,97	0,36	4,71	0,10	5,05	0,44	4,73	0,12	4,69	0,08	4,51	0,10	4,98	0,37	4,68	0,07	4,73	0,12	4,58	0,03	4,46	0,15	4,37	0,24
10	**4,49**	4,55	0,06	4,32	0,17	4,56	0,07	4,29	0,20	4,67	0,18	4,48	0,01	4,89	0,40	4,60	0,11	4,85	0,36	4,69	0,20	4,62	0,13	4,52	0,03
11	**1,31**	1,30	0,01	1,35	0,04	1,35	0,04	1,33	0,02	1,34	0,03	1,32	0,01	1,28	0,03	1,26	0,05	1,26	0,05	1,22	0,09	1,24	0,07	1,21	0,10
12	**1,27**	1,14	0,13	1,20	0,07	1,28	0,01	1,28	0,01	1,24	0,03	1,22	0,05	1,31	0,04	1,29	0,02	1,32	0,05	1,29	0,02	1,27	0,00	1,24	0,03
13	**4,03**	4,23	0,20	4,03	0,00	4,26	0,23	4,01	0,02	4,13	0,10	3,98	0,05	4,51	0,48	4,25	0,22	4,31	0,28	4,17	0,14	4,34	0,31	4,25	0,22
	**4,38**	4,63	0,25	4,39	0,01	4,61	0,23	4,33	0,05	4,61	0,23	4,43	0,05	4,58	0,20	4,31	0,07	4,39	0,01	4,24	0,14	4,35	0,03	4,26	0,12
14	**6,11**	6,89	0,78	6,47	0,36	6,93	0,82	6,46	0,35	6,41	0,30	6,15	0,04	6,77	0,66	6,33	0,22	6,47	0,36	6,26	0,15	6,45	0,34	6,32	0,21
15	**6,44**	6,56	0,12	6,17	0,27	6,62	0,18	6,18	0,26	6,59	0,15	6,32	0,12	6,50	0,06	6,09	0,35	6,44	0,00	6,23	0,21	6,41	0,03	6,28	0,16
16	**2,16**	2,20	0,04	2,17	0,01	2,26	0,10	2,17	0,01	2,27	0,11	2,20	0,04	2,25	0,09	2,16	0,00	2,21	0,05	2,14	0,02	2,20	0,04	2,15	0,01
17	**1,47**	1,43	0,04	1,47	0,00	1,50	0,03	1,48	0,01	1,50	0,03	1,46	0,01	1,50	0,03	1,47	0,00	1,47	0,00	1,43	0,04	1,48	0,01	1,44	0,03
18	**0,94**	1,00	0,06	1,07	0,13	1,02	0,08	1,03	0,09	0,94	0,00	0,93	0,01	0,98	0,04	0,99	0,05	0,98	0,04	0,95	0,01	0,99	0,05	0,96	0,02
**R^2**		**0,99676**		**0,99676**		**0,99485**		**0,99485**		**0,99687**		**0,99687**		**0,99638**		**0,99638**		**0,99597**		**0,99597**		**0,99617**		**0,99617**	
**MAE**			**0,16**		**0,09**		**0,22**		**0,13**		**0,17**		**0,09**		**0,20**		**0,11**		**0,14**		**0,12**		**0,12**		**0,12**

Table S11. Comparison of ^{13}C NMR data of compound **2** and calculated ^{13}C NMR data of 6 possible isomers of **1** in MeOH-d_4.

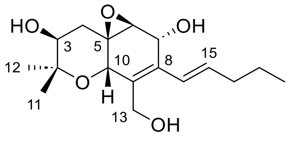

2_1

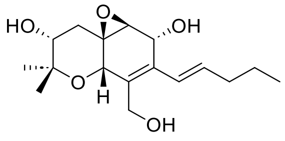

2_2

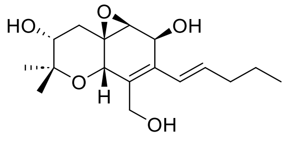

2_3

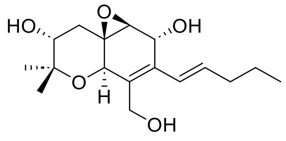

2_4

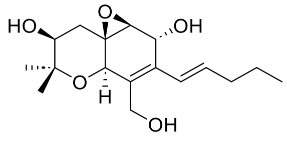

2_5

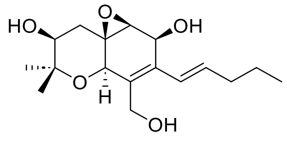

2_6

N°	Exp	2_1				2_2				2_3				2_4				2_5				2_6			
		unscaled	Δ	scaled	Δ	unscaled	Δ	scaled	Δ	unscaled	Δ	scaled	Δ	unscaled	Δ	scaled	Δ	unscaled	Δ	scaled	Δ	unscaled	Δ	scaled	Δ
2	**77,6**	80,8	3,2	78,7	1,1	79,8	2,2	77,3	0,3	79,4	1,8	76,2	1,4	81,9	4,3	80,2	2,6	81,1	3,5	78,7	1,1	80,6	3,0	77,9	0,3
3	**73,7**	77,5	3,8	75,3	1,6	76,5	2,8	73,9	0,2	76,3	2,6	73,0	0,7	76,3	2,6	74,5	0,8	75,3	1,6	72,8	0,9	75,5	1,8	72,8	0,9
4	**36,2**	37,3	1,1	34,4	1,8	39,4	3,2	35,8	0,4	39,5	3,3	35,8	0,4	37,7	1,5	35,0	1,2	36,3	0,1	33,2	3,0	36,7	0,5	33,6	2,6
5	**57**	60,1	3,1	57,6	0,6	59,0	2,0	55,8	1,2	61,9	4,9	58,5	1,5	63,0	6,0	60,9	3,9	60,2	3,2	57,5	0,5	61,2	4,2	58,3	1,3
6	**61,5**	65,0	3,5	62,6	1,1	65,1	3,6	62,1	0,6	66,6	5,1	63,2	1,7	61,8	0,3	59,7	1,8	59,2	2,3	56,5	5,0	61,2	0,3	58,4	3,1
7	**64,9**	68,0	3,1	65,6	0,7	68,1	3,2	65,2	0,3	71,1	6,2	67,8	2,9	68,0	3,1	66,0	1,1	71,4	6,5	68,9	4,0	72,1	7,2	69,3	4,4
8	**130,7**	135,4	4,7	134,2	3,5	135,8	5,1	134,9	4,2	138,7	8,0	136,2	5,5	132,0	1,3	131,4	0,7	135,6	4,9	134,0	3,3	139,3	8,6	137,2	6,5
9	**133,3**	132,4	0,9	131,2	2,1	132,5	0,8	131,5	1,8	132,9	0,4	130,3	3,0	135,4	2,1	134,9	1,6	134,2	0,9	132,5	0,8	133,1	0,2	130,9	2,4
10	**67,3**	71,1	3,8	68,8	1,5	73,7	6,4	71,0	3,7	71,6	4,3	68,3	1,0	71,8	4,5	69,9	2,6	73,6	6,3	71,1	3,8	72,4	5,1	69,6	2,3
11	**16,1**	16,3	0,2	13,1	3,0	22,9	6,8	18,7	2,6	22,8	6,7	18,9	2,8	28,3	12,2	25,5	9,4	25,7	9,6	22,5	6,4	25,7	9,6	22,5	6,4
12	**27,7**	28,1	0,4	25,0	2,7	26,6	1,1	22,6	5,1	26,5	1,2	22,6	5,1	16,1	11,6	13,0	14,7	22,2	5,5	19,0	8,7	22,0	5,7	18,7	9,0
13	**59,3**	62,0	2,7	59,5	0,2	64,6	5,3	61,7	2,4	64,4	5,1	61,0	1,7	60,8	1,5	58,6	0,7	63,1	3,8	60,4	1,1	62,1	2,8	59,3	0,0
14	**126,7**	122,9	3,8	121,5	5,2	124,5	2,2	123,3	3,4	122,8	3,9	120,1	6,6	123,1	3,6	122,3	4,4	124,2	2,5	122,4	4,3	123,9	2,8	121,6	5,1
15	**135,2**	136,9	1,7	135,8	0,6	134,9	0,3	134,0	1,2	139,5	4,3	137,0	1,8	133,8	1,4	133,3	1,9	136,8	1,6	135,2	0,0	137,4	2,2	135,3	0,1
16	**36,4**	41,5	5,1	38,6	2,2	40,8	4,4	37,2	0,8	40,4	4,0	36,7	0,3	40,5	4,1	37,9	1,5	40,7	4,3	37,8	1,4	40,8	4,4	37,7	1,3
17	**23,3**	28,8	5,5	25,8	2,5	28,6	5,3	24,6	1,3	27,4	4,1	23,5	0,2	28,4	5,1	25,5	2,2	28,3	5,0	25,2	1,9	28,3	5,0	25,1	1,8
18	**13,7**	16,2	2,5	12,9	0,8	15,4	1,7	11,0	2,7	15,4	1,7	11,4	2,3	15,1	1,4	11,9	1,8	16,0	2,3	12,7	1,0	15,9	2,2	12,6	1,1
**R^2**		**0,99850**		**0,99850**		**0,99823**		**0,99823**		**0,99740**		**0,99740**		**0,99325**		**0,99325**		**0,99604**		**0,99604**		**0,99561**		**0,99561**	
**MAE**			**2,90**		**1,84**		**3,32**		**1,90**		**3,98**		**2,28**		**3,91**		**3,11**		**3,76**		**2,77**		**3,86**		**2,86**

Functional	Solvent?	Basis Set	Type of Data
B3LYP	PCM	6-31+G(d,p)	Unscaled Shifts

	Isomer 1	Isomer 2	Isomer 3	Isomer 4	Isomer 5	Isomer 6
sDP4+ (H data)	34,28%	0,09%	58,80%	5,10%	0,71%	1,02%
sDP4+ (C data)	78,33%	21,48%	0,19%	0,00%	0,00%	0,00%
sDP4+ (all data)	99,51%	0,08%	0,41%	0,00%	0,00%	0,00%
uDP4+ (H data)	4,81%	0,01%	5,09%	0,02%	5,22%	84,84%
uDP4+ (C data)	53,25%	46,24%	0,51%	0,00%	0,00%	0,00%
uDP4+ (all data)	98,82%	0,17%	1,01%	0,00%	0,00%	0,00%
DP4+ (H data)	29,71%	0,00%	53,95%	0,02%	0,66%	15,66%
DP4+ (C data)	80,76%	19,24%	0,00%	0,00%	0,00%	0,00%
DP4+ (all data)	100,00%	0,00%	0,00%	0,00%	0,00%	0,00%

Figure S22. DP4+ probability analysis for the determination of the absolute configuration of **2**.

Table S12. Comparison of ^1H NMR data of compound **3** and calculated ^1H NMR data of 6 possible isomers of **3** in MeOH-d_4.

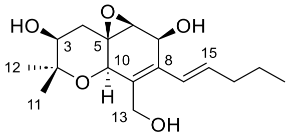

3_1

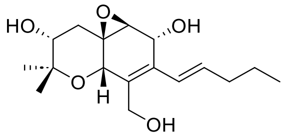

3_2

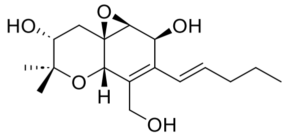

3_3

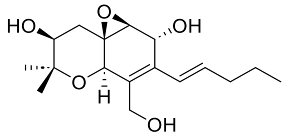

3_4

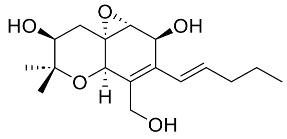

3_5

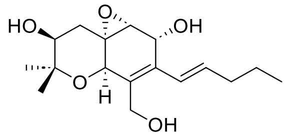

3_6

N°	Exp	3_1				3_2				3_3				3_4				3_5				3_6			
		unscaled	Δ	scaled	Δ	unscaled	Δ	scaled	Δ	unscaled	Δ	scaled	Δ	unscaled	Δ	scaled	Δ	unscaled	Δ	scaled	Δ	unscaled	Δ	scaled	Δ
3	**3,59**	3,79	0,20	3,71	0,12	3,99	0,40	3,75	0,16	3,96	0,37	3,81	0,22	3,81	0,22	3,68	0,09	3,99	0,40	3,78	0,19	3,97	0,38	3,77	0,18
4	**1,7**	1,53	0,17	1,47	0,23	1,32	0,38	1,29	0,41	1,32	0,38	1,26	0,44	1,57	0,13	1,50	0,20	1,32	0,38	1,29	0,41	1,30	0,40	1,27	0,43
	**2,36**	2,62	0,26	2,55	0,19	2,60	0,24	2,47	0,11	2,62	0,26	2,52	0,16	2,65	0,29	2,55	0,19	2,64	0,28	2,52	0,16	2,62	0,26	2,51	0,15
6	**3,29**	3,23	0,06	3,15	0,14	3,23	0,06	3,05	0,24	3,40	0,11	3,27	0,02	3,03	0,26	2,92	0,37	3,34	0,05	3,17	0,12	3,42	0,13	3,25	0,04
7	**4,6**	4,46	0,14	4,36	0,24	5,05	0,45	4,73	0,13	4,69	0,09	4,51	0,09	4,73	0,13	4,58	0,02	4,70	0,10	4,44	0,16	4,65	0,05	4,41	0,19
10	**4,49**	4,62	0,13	4,52	0,03	4,56	0,07	4,28	0,21	4,67	0,18	4,49	0,00	4,85	0,36	4,69	0,20	4,73	0,24	4,47	0,02	4,54	0,05	4,31	0,18
11	**1,15**	1,24	0,09	1,18	0,03	1,35	0,20	1,31	0,16	1,34	0,19	1,29	0,14	1,26	0,11	1,20	0,05	1,23	0,08	1,21	0,06	1,24	0,09	1,21	0,06
12	**1,12**	1,27	0,15	1,22	0,10	1,28	0,16	1,25	0,13	1,24	0,12	1,19	0,07	1,32	0,20	1,26	0,14	1,34	0,22	1,31	0,19	1,35	0,23	1,31	0,19
13	**4,14**	4,34	0,20	4,24	0,10	4,26	0,12	4,00	0,14	4,13	0,01	3,97	0,17	4,31	0,17	4,16	0,02	4,25	0,11	4,02	0,12	4,22	0,08	4,01	0,13
	**4,38**	4,35	0,03	4,26	0,12	4,61	0,23	4,33	0,05	4,61	0,23	4,43	0,05	4,39	0,01	4,24	0,14	4,60	0,22	4,35	0,03	4,52	0,14	4,29	0,09
14	**6,4**	6,45	0,05	6,32	0,08	6,93	0,53	6,47	0,07	6,41	0,01	6,17	0,23	6,47	0,07	6,27	0,13	6,85	0,45	6,44	0,04	6,68	0,28	6,32	0,08
15	**6,05**	6,41	0,36	6,28	0,23	6,62	0,57	6,19	0,14	6,59	0,54	6,34	0,29	6,44	0,39	6,24	0,19	6,60	0,55	6,21	0,16	6,84	0,79	6,46	0,41
16	**2,13**	2,20	0,07	2,13	0,00	2,26	0,13	2,16	0,03	2,27	0,14	2,18	0,05	2,21	0,08	2,12	0,01	2,21	0,08	2,12	0,01	2,29	0,16	2,19	0,06
17	**1,44**	1,48	0,04	1,42	0,02	1,50	0,06	1,46	0,02	1,50	0,06	1,44	0,00	1,47	0,03	1,41	0,03	1,47	0,03	1,43	0,01	1,56	0,12	1,51	0,07
18	**0,91**	0,99	0,08	0,93	0,02	1,02	0,11	1,01	0,10	0,94	0,03	0,90	0,01	0,98	0,07	0,93	0,02	1,01	0,10	1,00	0,09	0,95	0,04	0,93	0,02
**R^2**		**0,99697**		**0,99697**		**0,99538**		**0,99538**		**0,99488**		**0,99488**		**0,99608**		**0,99608**		**0,99609**		**0,99609**		**0,99385**		**0,99385**	
**MAE**			**0,13**		**0,11**		**0,25**		**0,14**		**0,18**		**0,13**		**0,17**		**0,12**		**0,22**		**0,12**		**0,21**		**0,15**

Table S13. Comparison of ^{13}C NMR data of compound **3** and calculated ^{13}C NMR data of 6 possible isomers of **3** in MeOH-d_4.

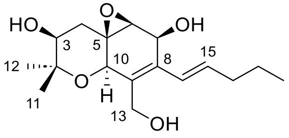

3_1

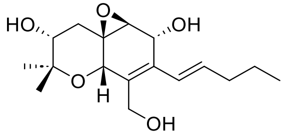

3_2

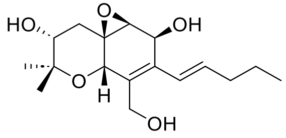

3_3

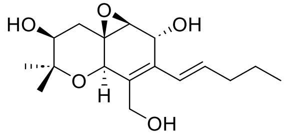

3_4

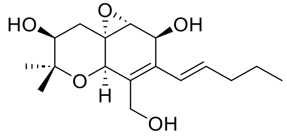

3_5

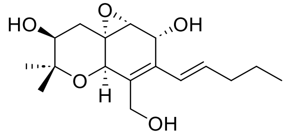

3_6

N°	Exp	3_1				3_2				3_3				3_4				3_5				3_6			
		unscaled	Δ	scaled	Δ	unscaled	Δ	scaled	Δ	unscaled	Δ	scaled	Δ	unscaled	Δ	scaled	Δ	unscaled	Δ	scaled	Δ	unscaled	Δ	scaled	Δ
2	**74,8**	80,6	5,8	77,9	3,1	79,8	5,0	77,4	2,6	79,4	4,6	76,2	1,4	81,1	6,3	78,7	3,9	80,1	5,3	77,6	2,8	79,3	4,5	76,0	1,2
3	**73,1**	75,5	2,4	72,8	0,3	76,5	3,4	74,0	0,9	76,3	3,2	73,1	0,0	75,3	2,2	72,9	0,2	76,7	3,6	74,2	1,1	76,3	3,2	72,9	0,2
4	**34,1**	36,7	2,6	34,0	0,1	39,4	5,3	36,1	2,0	39,5	5,4	36,2	2,1	36,3	2,2	33,6	0,5	39,3	5,2	36,1	2,0	39,7	5,6	36,2	2,1
5	**59,2**	61,2	2,0	58,5	0,7	59,0	0,2	56,0	3,2	61,9	2,7	58,7	0,5	60,2	1,0	57,7	1,5	58,4	0,8	55,6	3,6	61,8	2,6	58,4	0,8
6	**59,5**	61,2	1,7	58,6	0,9	65,1	5,6	62,3	2,8	66,6	7,1	63,4	3,9	59,2	0,3	56,7	2,8	64,2	4,7	61,5	2,0	66,5	7,0	63,1	3,6
7	**64,8**	72,1	7,3	69,4	4,6	68,1	3,3	65,4	0,6	71,1	6,3	67,9	3,1	71,4	6,6	69,0	4,2	69,2	4,4	66,5	1,7	71,3	6,5	67,9	3,1
8	**131,5**	139,3	7,8	136,7	5,2	135,8	4,3	134,5	3,0	138,7	7,2	135,8	4,3	135,6	4,1	133,6	2,1	137,2	5,7	135,7	4,2	140,7	9,2	137,4	5,9
9	**133,8**	133,1	0,7	130,4	3,4	132,5	1,3	131,2	2,6	132,9	0,9	130,0	3,8	134,2	0,4	132,1	1,7	131,9	1,9	130,3	3,5	131,3	2,5	128,0	5,8
10	**67,4**	72,4	5,0	69,7	2,3	73,7	6,3	71,1	3,7	71,6	4,2	68,4	1,0	73,6	6,2	71,2	3,8	71,9	4,5	69,3	1,9	74,2	6,8	70,8	3,4
11	**24,5**	25,7	1,2	23,1	1,4	22,9	1,6	19,2	5,3	22,8	1,7	19,4	5,1	25,7	1,2	19,5	5,0	26,5	2,0	23,2	1,3	26,7	2,2	23,2	1,3
12	**25,5**	22,0	3,5	19,3	6,2	26,6	1,1	23,0	2,5	26,5	1,0	23,1	2,4	22,2	3,3	23,0	2,5	22,9	2,6	19,5	6,0	22,9	2,6	19,4	6,1
13	**59,6**	62,1	2,5	59,5	0,1	64,6	5,0	61,8	2,2	64,4	4,8	61,1	1,5	63,1	3,5	60,6	1,0	62,0	2,4	59,2	0,4	65,9	6,3	62,5	2,9
14	**126,7**	123,9	2,8	121,2	5,5	124,5	2,2	123,0	3,7	122,8	3,9	119,9	6,8	124,2	2,5	122,1	4,6	123,2	3,5	121,5	5,2	123,8	2,9	120,5	6,2
15	**134,8**	137,4	2,6	134,7	0,1	134,9	0,1	133,6	1,2	139,5	4,7	136,6	1,8	136,8	2,0	134,8	0,0	137,4	2,6	135,9	1,1	138,6	3,8	135,4	0,6
16	**36,4**	40,8	4,4	38,1	1,7	40,8	4,4	37,5	1,1	40,4	4,0	37,1	0,7	40,7	4,3	38,1	1,7	41,6	5,2	38,5	2,1	39,5	3,1	36,1	0,3
17	**23,3**	28,3	5,0	25,6	2,3	28,6	5,3	25,0	1,7	27,4	4,1	24,0	0,7	28,3	5,0	25,6	2,3	28,7	5,4	25,3	2,0	27,2	3,9	23,8	0,5
18	**13,8**	15,9	2,1	13,2	0,6	15,4	1,6	11,5	2,3	15,4	1,6	12,0	1,8	16,0	2,2	13,2	0,6	16,2	2,4	12,7	1,1	14,6	0,8	11,1	2,7
**R^2**		**0,99718**		**0,99718**		**0,99775**		**0,99775**		**0,99719**		**0,99719**		**0,99757**		**0,99770**		**0,99740**		**0,99740**		**0,99631**		**0,99631**	
**MAE**			**3,50**		**2,26**		**3,31**		**2,44**		**3,96**		**2,41**		**3,13**		**2,25**		**3,65**		**2,48**		**4,33**		**2,74**

Functional	Solvent?	Basis Set	Type of Data
B3LYP	PCM	6-31+G(d,p)	Unscaled Shifts

	Isomer 1	Isomer 2	Isomer 3	Isomer 4	Isomer 5	Isomer 6
sDP4+ (H data)	59,77%	1,03%	3,47%	13,40%	22,16%	0,16%
sDP4+ (C data)	19,44%	17,63%	6,92%	49,63%	6,32%	0,07%
sDP4+ (all data)	57,83%	0,91%	1,20%	33,09%	6,97%	0,00%
uDP4+ (H data)	97,77%	0,00%	0,11%	1,93%	0,05%	0,14%
uDP4+ (C data)	0,20%	88,19%	2,27%	0,13%	9,16%	0,05%
uDP4+ (all data)	94,57%	0,75%	1,24%	1,29%	2,12%	0,03%
DP4+ (H data)	99,53%	0,00%	0,01%	0,44%	0,02%	0,00%
DP4+ (C data)	0,23%	94,87%	0,96%	0,41%	3,53%	0,00%
DP4+ (all data)	98,92%	0,01%	0,03%	0,77%	0,27%	0,00%

Figure S23. DP4+ probability analysis for the determination of the absolute configuration of **3**.

Single Mass Analysis
Tolerance = 5.0 mDa / DBE: min = -1.5, max = 50.0
Element prediction: Off
Number of isotope peaks used for i-FIT = 3
Monoisotopic Mass, Even Electron Ions
229 formula(e) evaluated with 4 results within limits (up to 50 closest results for each mass)
Elements Used:

Mass	Calc. Mass	mDa	PPM	DBE	Formula	i-FIT	i-FIT Norm	Fit Conf %	C	H	O	Na
351.1784	351.1784	0.0	0.0	3.5	C17 H28 O6 Na	212.3	1.982	13.78	17	28	6	1
	351.1760	2.4	6.8	0.5	C15 H29 O6 Na2	213.7	3.455	3.16	15	29	6	2
	351.1808	-2.4	-6.8	6.5	C19 H27 O6	211.0	0.702	49.55	19	27	6	
	351.1749	3.5	10.0	15.5	C26 H23 O	211.4	1.093	33.51	26	23	1	

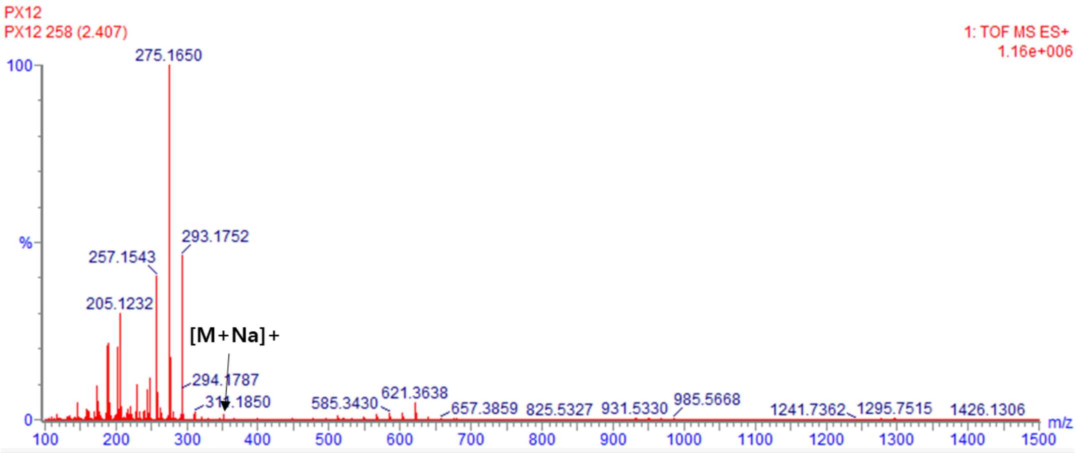

Figure S24. HR-ESIMS data of compound **4**.

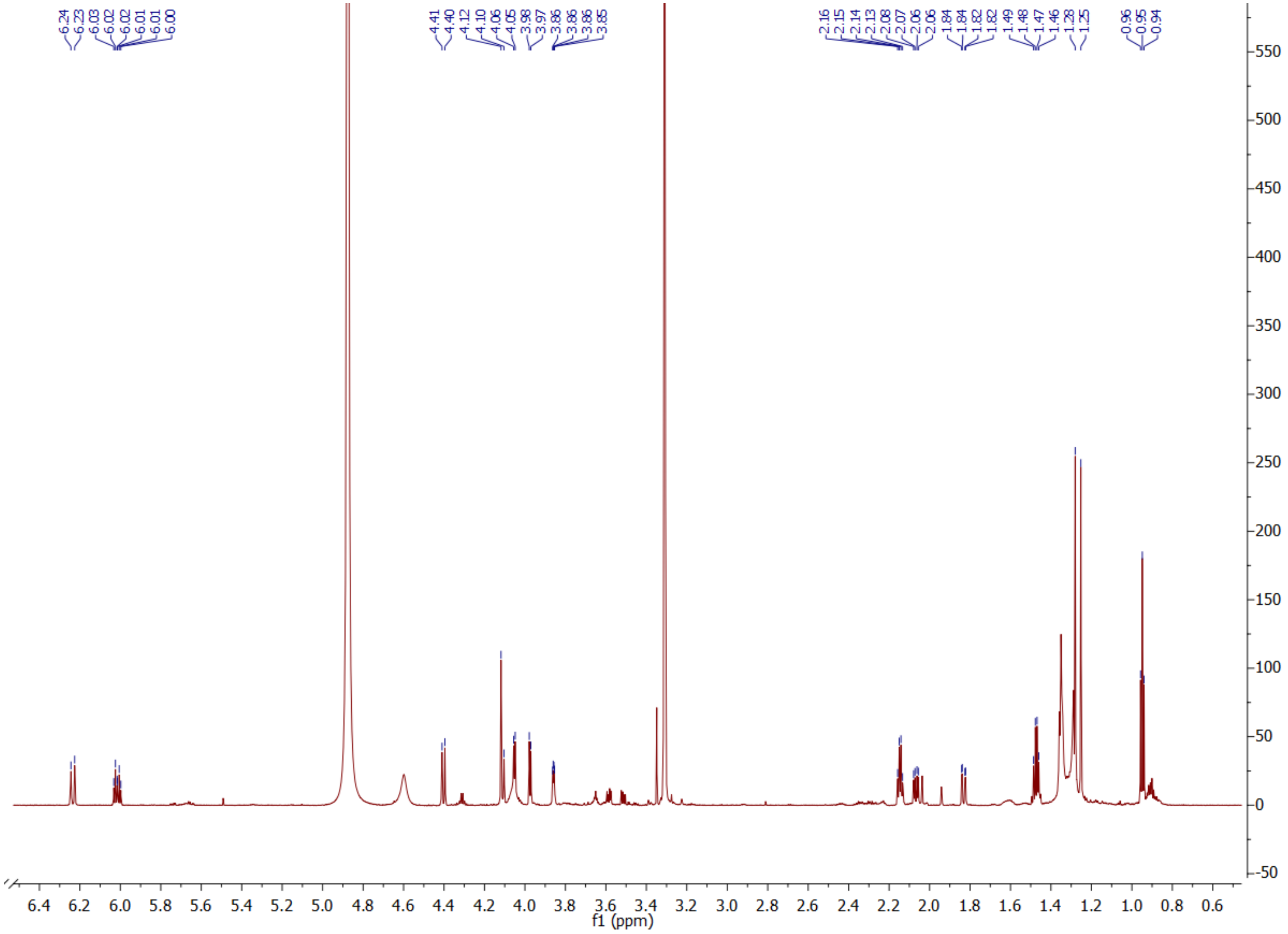

Figure S25. ^{1}H NMR spectrum of compound **4**.

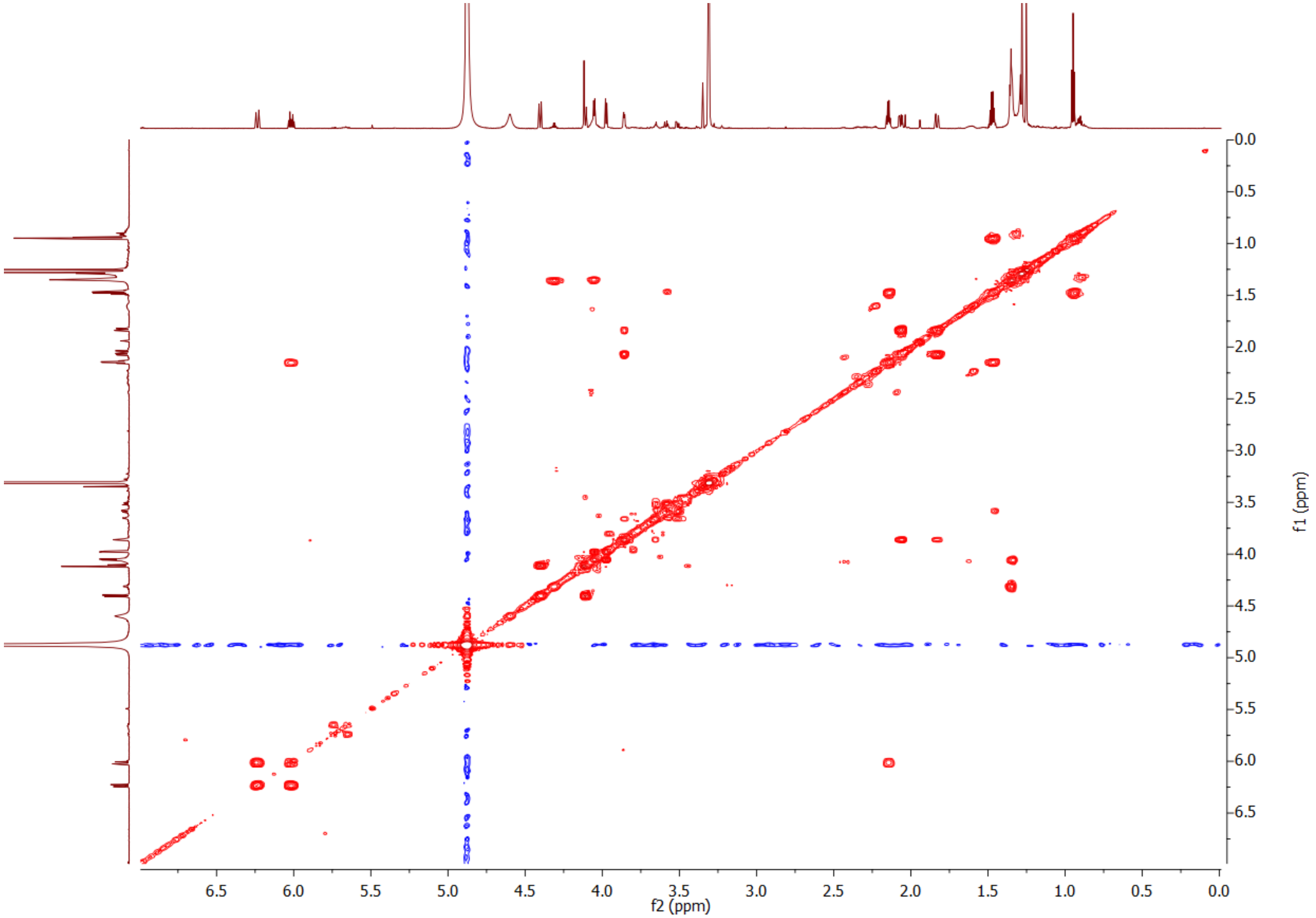

Figure S26. ^{1}H-^{1}H COSY spectrum of compound **4**.

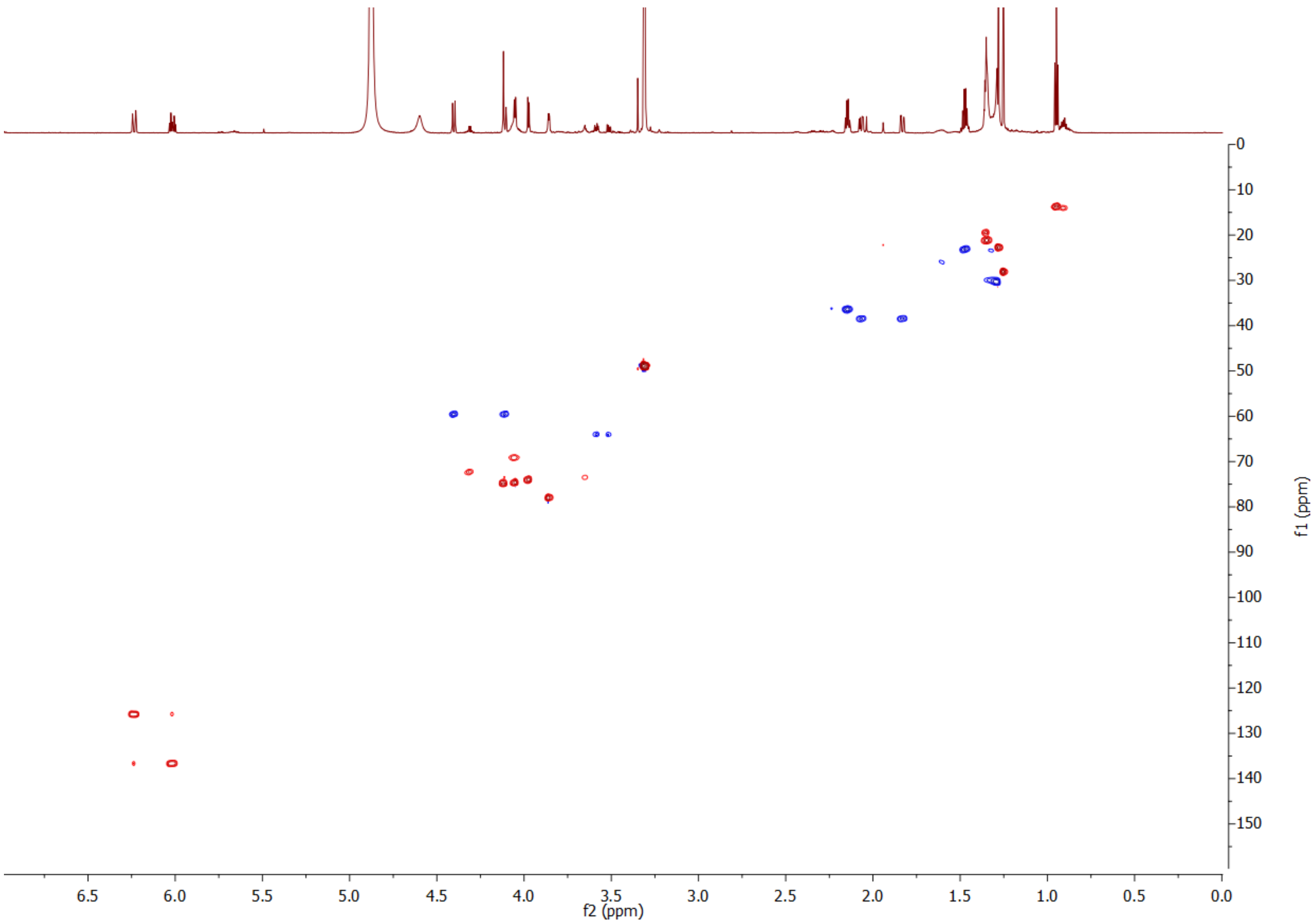

Figure S27. ^{1}H-^{13}C HSQC spectrum of compound **4**.

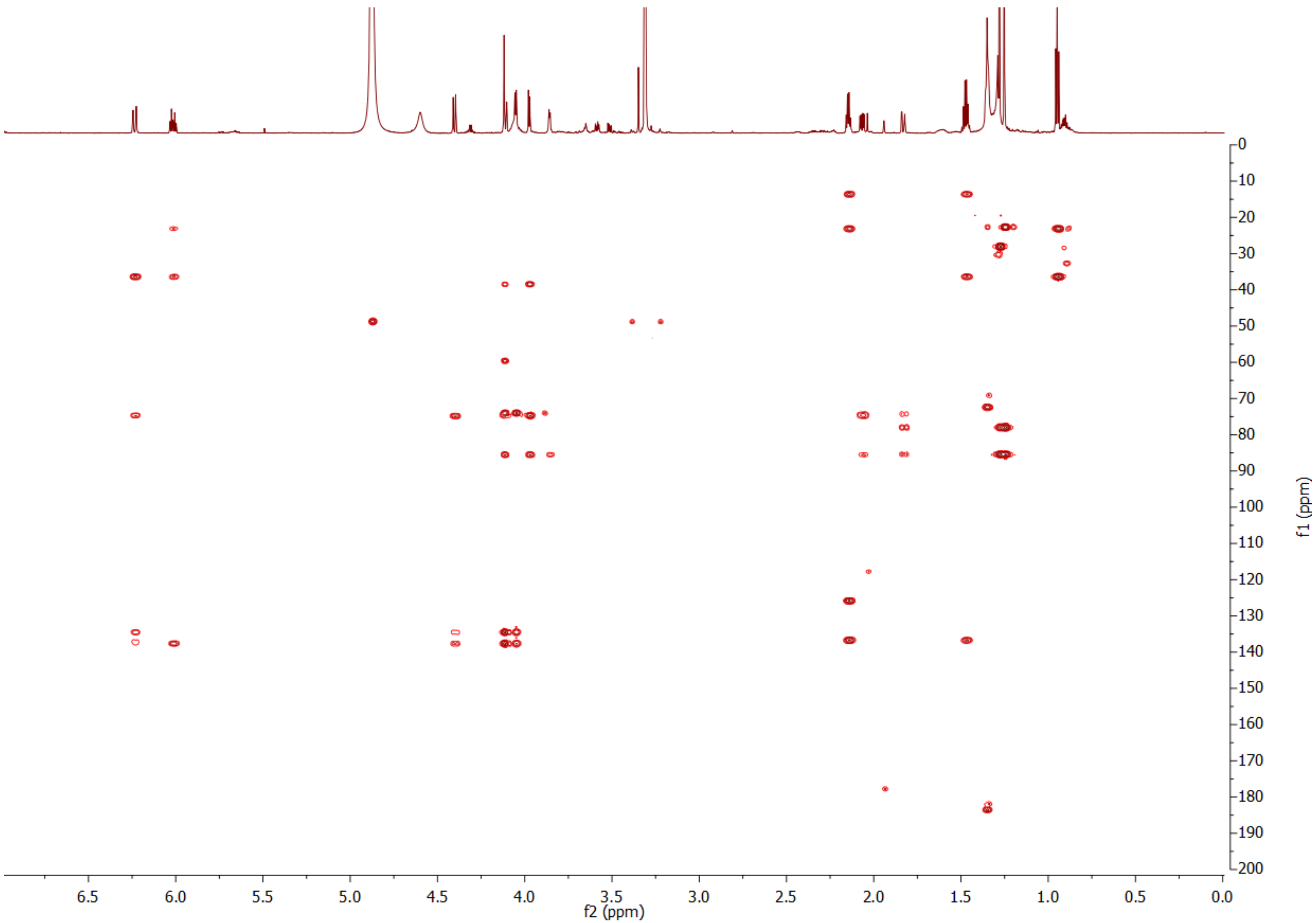

Figure S28. ^{1}H-^{13}C HMBC spectrum of compound **4**.

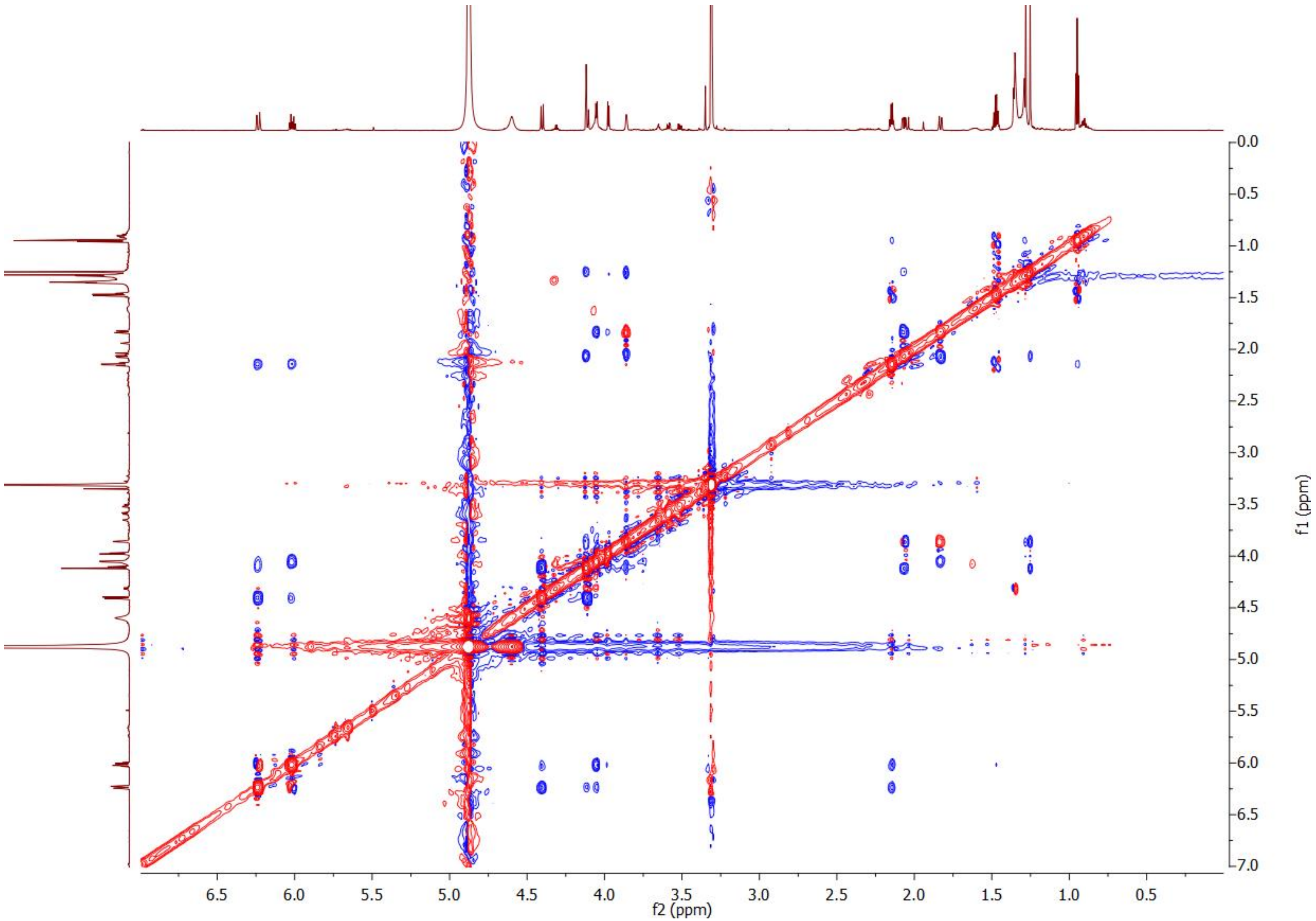

Figure S29. ^1H-^1H ROESY spectrum of compound **4**.

Table S14. Comparison of ^{1}H NMR data of compound **4** and calculated ^{1}H NMR data of 7 possible isomers of **4** in MeOH-d_4.

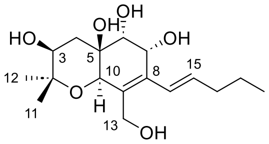

4_1

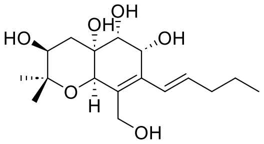

4_2

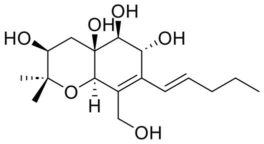

4_3

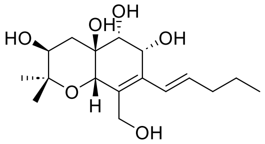

4_4

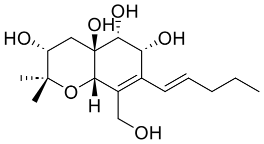

4_5

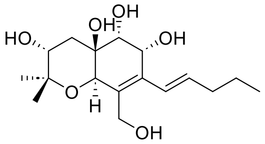

4_6

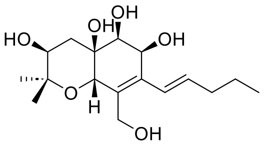

4_7

N°	Exp	4_1				4_2				4_3				4_4				4_5				4_6				4_7			
		unscaled	Δ	scaled	Δ	unscaled	Δ	scaled	Δ	unscaled	Δ	scaled	Δ	unscaled	Δ	scaled	Δ	unscaled	Δ	scaled	Δ	unscaled	Δ	scaled	Δ	unscaled	Δ	scaled	Δ
3	**3,86**	3,92	0,06	3,77	0,09	4,11	0,25	3,92	0,06	3,88	0,02	3,75	0,11	4,35	0,49	4,13	0,27	3,49	0,37	3,35	0,51	3,93	0,07	3,73	0,13	3,69	0,17	3,45	0,41
4	**2,07**	2,35	0,28	2,28	0,21	1,68	0,39	1,75	0,32	2,32	0,25	2,26	0,19	2,03	0,04	1,93	0,14	2,32	0,25	2,20	0,13	1,74	0,33	1,69	0,38	2,10	0,03	2,04	0,03
	**1,83**	1,74	0,09	1,70	0,13	1,28	0,55	1,39	0,44	1,94	0,11	1,90	0,07	2,02	0,19	1,92	0,09	2,12	0,29	2,00	0,17	2,37	0,54	2,27	0,44	1,60	0,23	1,59	0,24
6	**3,97**	3,73	0,24	3,59	0,38	3,67	0,30	3,53	0,44	3,41	0,56	3,30	0,67	3,67	0,30	3,49	0,48	3,88	0,09	3,74	0,23	3,76	0,21	3,57	0,40	4,26	0,29	3,97	0,00
7	**4,05**	4,44	0,39	4,26	0,21	4,35	0,30	4,14	0,09	4,40	0,35	4,24	0,19	4,42	0,37	4,20	0,15	4,61	0,56	4,46	0,41	4,50	0,45	4,26	0,21	5,02	0,97	4,65	0,60
10	**4,11**	4,71	0,60	4,52	0,41	4,89	0,78	4,62	0,51	4,53	0,42	4,37	0,26	4,72	0,61	4,48	0,37	4,62	0,51	4,47	0,36	4,88	0,77	4,60	0,49	4,31	0,20	4,01	0,10
11	**1,28**	1,23	0,05	1,26	0,02	1,07	0,21	1,20	0,08	1,23	0,05	1,22	0,06	1,32	0,04	1,26	0,09	1,31	0,03	1,21	0,07	1,23	0,05	1,21	0,07	1,25	0,03	1,28	0,00
12	**1,25**	1,27	0,02	1,22	0,03	1,28	0,03	1,39	0,14	1,24	0,01	1,23	0,02	1,25	0,00	1,19	0,01	1,18	0,07	1,08	0,17	1,28	0,03	1,26	0,01	1,18	0,07	1,22	0,03
13	**4,40**	4,43	0,03	4,25	0,15	4,77	0,37	4,51	0,11	4,67	0,27	4,50	0,10	4,72	0,32	4,49	0,09	4,69	0,29	4,54	0,14	4,71	0,31	4,45	0,05	4,55	0,15	4,22	0,18
	**4,12**	4,07	0,05	3,91	0,21	4,65	0,53	4,40	0,28	4,10	0,02	3,96	0,16	4,48	0,36	4,26	0,14	4,50	0,38	4,36	0,24	4,16	0,04	3,94	0,18	4,29	0,17	3,99	0,13
14	**6,23**	6,55	0,32	6,26	0,03	6,61	0,38	6,16	0,07	6,29	0,06	6,05	0,18	6,41	0,18	6,09	0,14	6,18	0,05	6,00	0,23	6,34	0,11	5,96	0,27	6,70	0,47	6,14	0,09
15	**6,01**	6,36	0,35	6,08	0,07	6,21	0,20	5,80	0,21	6,62	0,61	6,36	0,35	6,16	0,15	5,85	0,16	6,08	0,07	5,91	0,10	6,68	0,67	6,28	0,27	6,81	0,80	6,25	0,24
16	**2,15**	2,21	0,06	2,15	0,00	2,23	0,08	2,24	0,09	2,22	0,07	2,17	0,02	2,21	0,06	2,10	0,05	2,29	0,14	2,17	0,02	2,20	0,05	2,11	0,04	2,36	0,21	2,27	0,12
17	**1,47**	1,49	0,02	1,46	0,01	1,50	0,03	1,58	0,11	1,49	0,02	1,47	0,00	1,49	0,02	1,42	0,05	1,53	0,06	1,42	0,05	1,48	0,01	1,44	0,03	1,56	0,09	1,56	0,09
18	**0,95**	1,03	0,08	1,02	0,07	0,99	0,04	1,13	0,18	0,97	0,02	0,97	0,02	0,98	0,03	0,94	0,01	0,93	0,02	0,83	0,12	1,01	0,06	1,00	0,05	1,05	0,10	1,10	0,15
**R^2**		0,99399		0,99402		**0,98836**		**0,98836**		**0,99056**		**0,99056**		**0,99315**		**0,99315**		**0,99009**		**0,99009**		**0,98827**		**0,98827**		**0,99123**		**0,99123**	
**MAE**			0,18		0,13		**0,30**		**0,21**		**0,19**		**0,16**		**0,21**		**0,15**		**0,21**		**0,20**		**0,25**		**0,20**		**0,27**		**0,16**

Table S15. Comparison of ^{13}C NMR data of compound **4** and calculated ^{13}C NMR data of 7 possible isomers of **4** in MeOH-d_4.

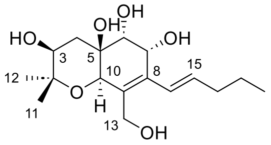

4_1

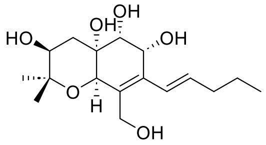

4_2

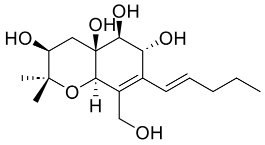

4_3

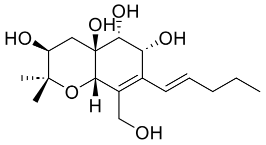

4_4

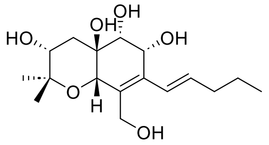

4_5

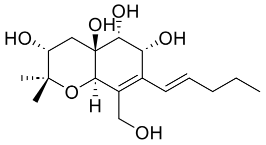

4_6

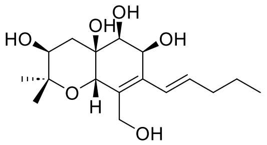

4_7

N°	Exp	4_1				4_2				4_3				4_4				4_5				4_6				4_7			
		unscaled	Δ	scaled	Δ	unscaled	Δ	scaled	Δ	unscaled	Δ	scaled	Δ	unscaled	Δ	scaled	Δ	unscaled	Δ	scaled	Δ	unscaled	Δ	scaled	Δ	unscaled	Δ	scaled	Δ
2	**85,4**	79,5	5,9	79,6	5,8	80,2	5,2	79,8	5,6	79,8	5,6	79,7	5,7	80,9	4,5	81,1	4,3	80,7	4,7	81,5	3,9	79,6	5,8	80,0	5,4	79,7	5,7	80,9	4,5
3	**78**	77,4	0,6	77,5	0,5	73,4	4,6	72,9	5,1	76,2	1,8	76,1	1,9	74,7	3,3	74,7	3,3	74,4	3,6	74,9	3,1	77,4	0,6	77,6	0,4	74,6	3,4	75,7	2,3
4	**38,5**	36,4	2,1	36,1	2,4	39,4	0,9	38,4	0,1	35,6	2,9	35,2	3,3	46,8	8,3	46,1	7,6	45,0	6,5	44,9	6,4	36,6	1,9	36,4	2,1	39,7	1,2	39,9	1,4
5	**85,5**	76,1	9,4	76,2	9,3	78,9	6,6	78,5	7,0	75,3	10,2	75,2	10,3	72,4	13,1	72,4	13,1	70,8	14,7	71,2	14,3	76,3	9,2	76,6	8,9	77,0	8,5	78,2	7,3
6	**74**	78,4	4,4	78,4	4,4	79,7	5,7	79,3	5,3	79,6	5,6	79,6	5,6	80,3	6,3	80,5	6,5	74,7	0,7	75,3	1,3	78,4	4,4	78,7	4,7	70,8	3,2	71,8	2,2
7	**74,6**	72,7	1,9	72,7	1,9	74,4	0,2	73,9	0,7	79,1	4,5	79,0	4,4	72,8	1,8	72,8	1,8	71,9	2,7	72,4	2,2	72,3	2,3	72,6	2,0	71,9	2,7	72,9	1,7
8	**134,5**	142,9	8,4	143,6	9,1	138,0	3,5	138,4	3,9	140,5	6,0	141,0	6,5	142,2	7,7	144,1	9,6	137,9	3,4	140,0	5,5	142,3	7,8	143,5	9,0	138,7	4,2	141,4	6,9
9	**137,6**	134,8	2,8	135,4	2,2	137,3	0,3	137,7	0,1	135,1	2,5	135,5	2,1	130,0	7,6	131,5	6,1	133,8	3,8	135,9	1,7	134,0	3,6	135,0	2,6	133,9	3,7	136,5	1,1
10	**74,8**	73,4	1,4	73,5	1,3	76,1	1,3	75,7	0,9	76,0	1,2	76,0	1,2	72,3	2,5	72,3	2,5	71,4	3,4	71,9	2,9	72,4	2,4	72,6	2,2	78,3	3,5	79,5	4,7
11	**22,7**	25,4	2,7	25,0	2,3	18,9	3,8	17,6	5,1	25,4	2,7	24,8	2,1	16,8	5,9	15,2	7,5	22,6	0,1	21,9	0,8	25,5	2,8	25,1	2,4	16,5	6,2	16,1	6,6
12	**28,1**	22,1	6,0	21,6	6,5	30,0	1,9	28,9	0,8	21,9	6,2	21,3	6,8	28,6	0,5	27,3	0,8	26,9	1,2	26,3	1,8	22,1	6,0	21,7	6,4	29,1	1,0	29,1	1,0
13	**59,6**	62,5	2,9	62,4	2,8	62,7	3,1	62,0	2,4	60,9	1,3	60,7	1,1	62,3	2,7	62,0	2,4	62,0	2,4	62,3	2,7	61,0	1,4	61,1	1,5	61,2	1,6	61,9	2,3
14	**125,8**	126,7	0,9	127,3	1,5	123,2	2,6	123,4	2,4	124,0	1,8	124,4	1,4	124,2	1,6	125,6	0,2	120,8	5,0	122,5	3,3	125,8	0,0	126,7	0,9	123,0	2,8	125,3	0,5
15	**136,7**	135,3	1,4	135,9	0,8	140,8	4,1	141,2	4,5	137,8	1,1	138,3	1,6	139,9	3,2	141,7	5,0	144,9	8,2	147,2	10,5	136,1	0,6	137,2	0,5	135,2	1,5	137,8	1,1
16	**36,4**	40,5	4,1	40,2	3,8	40,7	4,3	39,7	3,3	40,3	3,9	39,9	3,5	40,9	4,5	40,0	3,6	40,0	3,6	39,7	3,3	40,8	4,4	40,6	4,2	39,7	3,3	39,9	3,5
17	**23,1**	29,0	5,9	28,6	5,5	28,2	5,1	27,1	4,0	28,0	4,9	27,5	4,4	28,6	5,5	27,3	4,2	27,1	4,0	26,5	3,4	28,7	5,6	28,3	5,2	28,3	5,2	28,2	5,1
18	**13,7**	15,8	2,1	15,2	1,5	15,8	2,1	14,4	0,7	15,6	1,9	15,0	1,3	16,1	2,4	14,4	0,7	15,4	1,7	14,6	0,9	16,0	2,3	15,5	1,8	14,2	0,5	13,7	0,0
R^2		**0,99397**		**0,99397**		**0,99589**		**0,99589**		**0,99402**		**0,99402**		**0,99033**		**0,99033**		**0,99177**		**0,99177**		**0,99424**		**0,99424**		**0,99566**		**0,99566**	
**MAE**			**3,70**		**3,63**		**3,24**		**3,04**		**3,78**		**3,71**		**4,78**		**4,66**		**4,11**		**4,00**		**3,59**		**3,54**		**3,41**		**3,07**

Functional	Solvent?	Basis Set	Type of Data
B3LYP	PCM	6-31+G(d,p)	Unscaled Shifts

	Isomer 1	Isomer 2	Isomer 3	Isomer 4	Isomer 5	Isomer 6	Isomer 7
sDP4+ (H data)	74,53%	0,00%	4,64%	16,45%	0,01%	0,01%	4,36%
sDP4+ (C data)	0,05%	58,61%	0,02%	0,00%	0,00%	0,14%	41,18%
sDP4+ (all data)	2,13%	0,09%	0,06%	0,00%	0,00%	0,00%	97,72%
uDP4+ (H data)	99,25%	0,00%	0,59%	0,15%	0,00%	0,01%	0,00%
uDP4+ (C data)	0,33%	84,08%	15,46%	0,00%	0,00%	0,08%	0,06%
uDP4+ (all data)	78,11%	0,02%	21,87%	0,00%	0,00%	0,00%	0,00%
DP4+ (H data)	99,93%	0,00%	0,04%	0,03%	0,00%	0,00%	0,00%
DP4+ (C data)	0,00%	99,94%	0,01%	0,00%	0,00%	0,00%	0,05%
DP4+ (all data)	99,17%	0,00%	0,79%	0,00%	0,00%	0,00%	0,03%

Figure S30. DP4+ probability analysis for the determination of the absolute configuration of **4**.

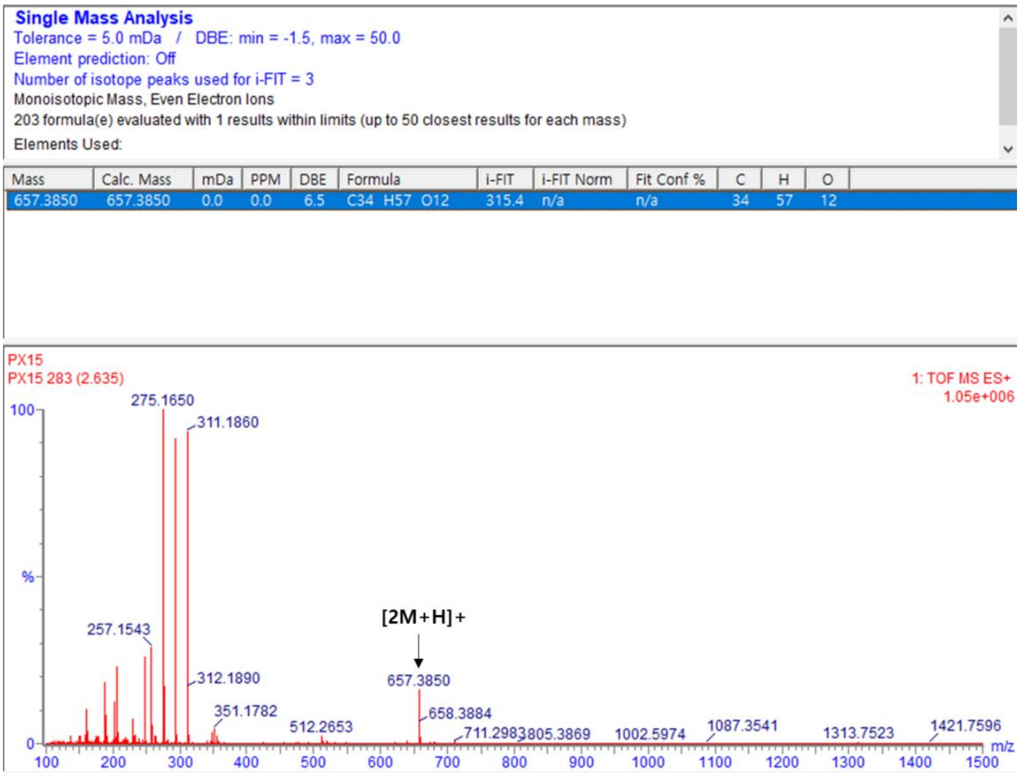

Figure S31. HR-ESIMS data of compound **5**.

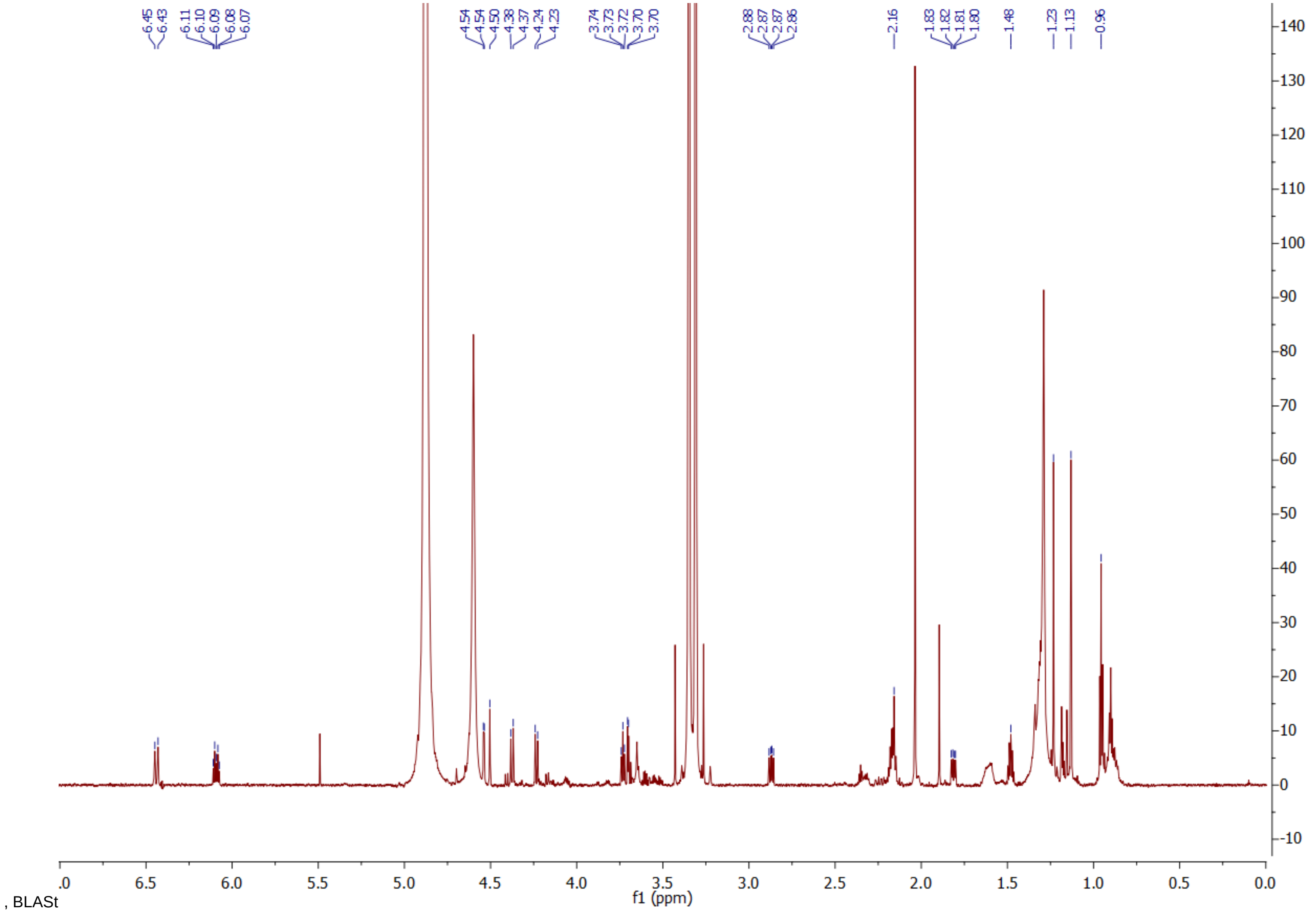
6.45
6.43
6.11
6.10
6.09
6.08
6.07
4.54
4.54
4.50
4.38
4.37
4.24
4.23
3.74
3.73
3.72
3.70
3.70
2.88
2.87
2.87
2.86
2.16
1.83
1.82
1.81
1.80
1.48
1.23
1.13
0.96
f1 (ppm)

, BLASt

Figure S32. ^{1}H NMR spectrum of compound **5**.

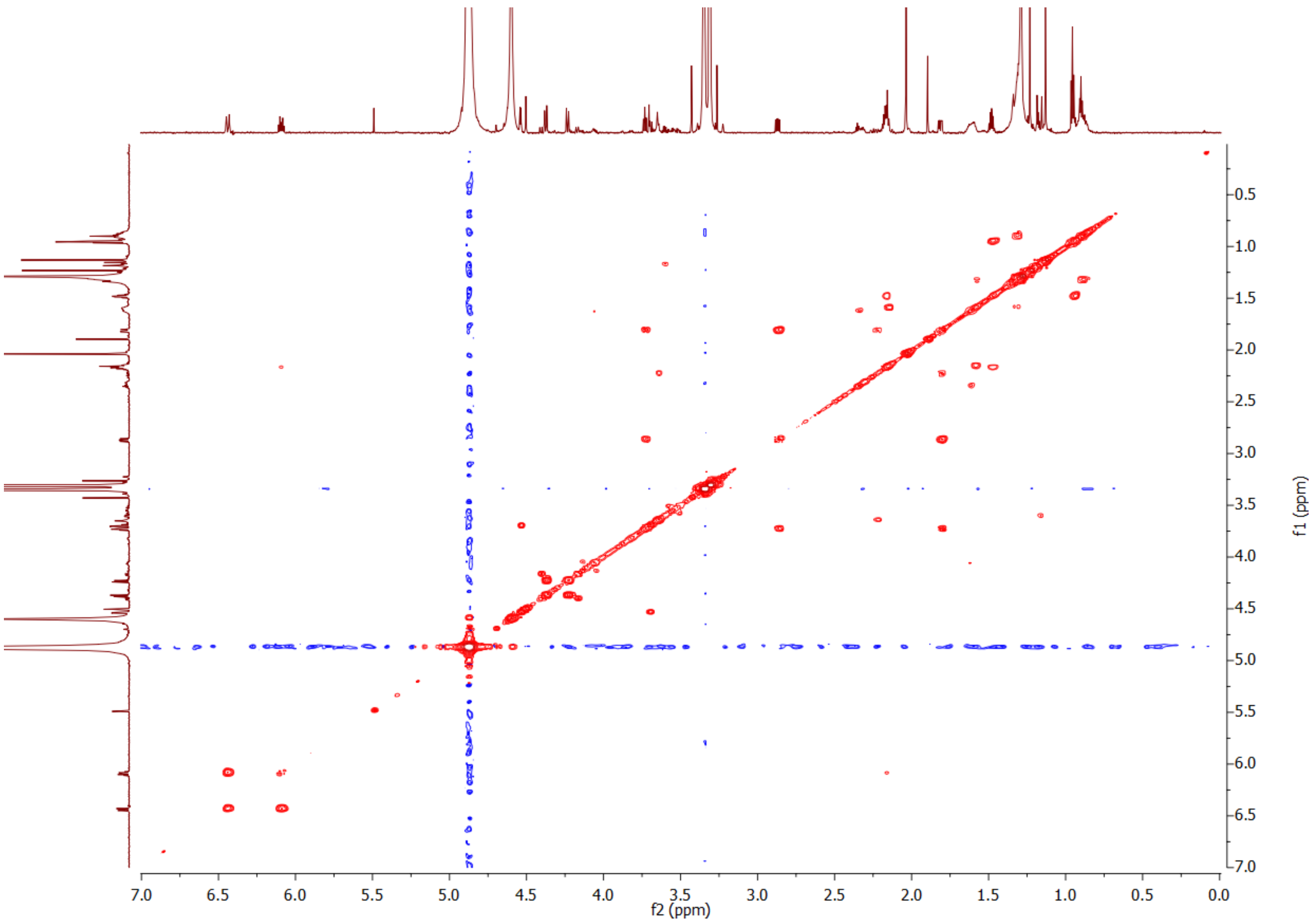

Figure S33. ^{1}H-^{1}H COSY spectrum of compound **5**.

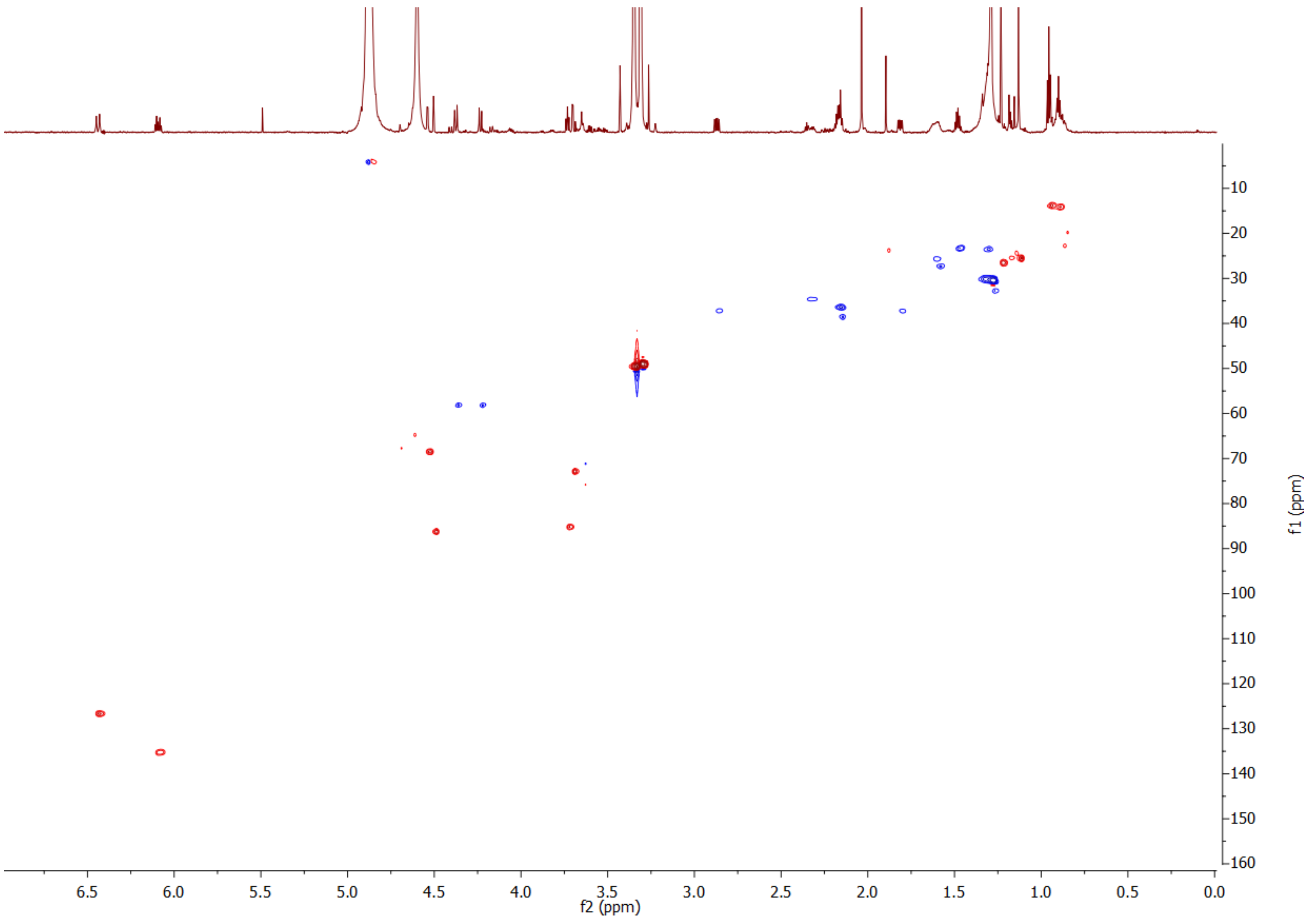

Figure S34. ^{1}H-^{13}C HSQC spectrum of compound **5**.

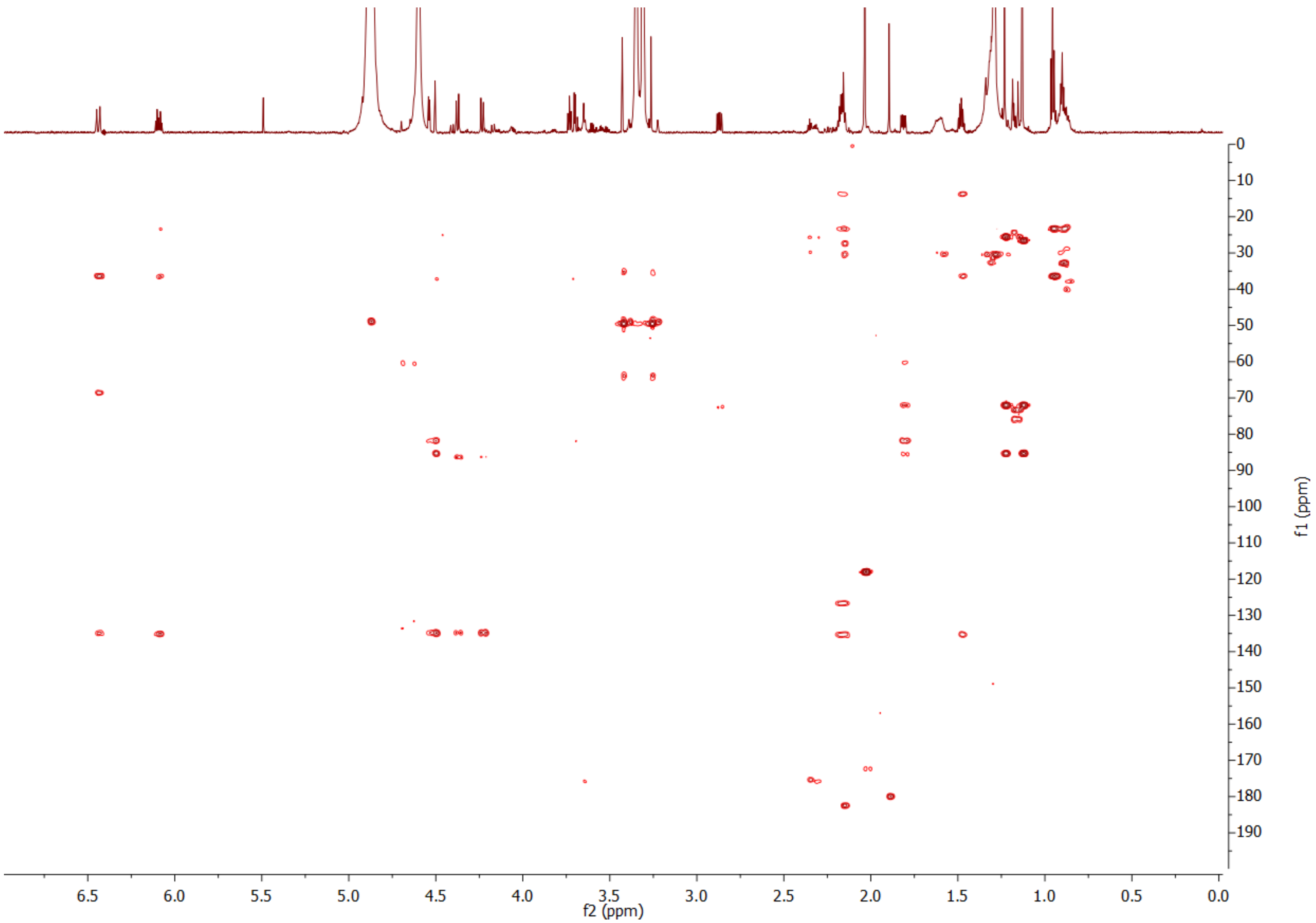

Figure S35. ^{1}H-^{13}C HMBC spectrum of compound **5**.

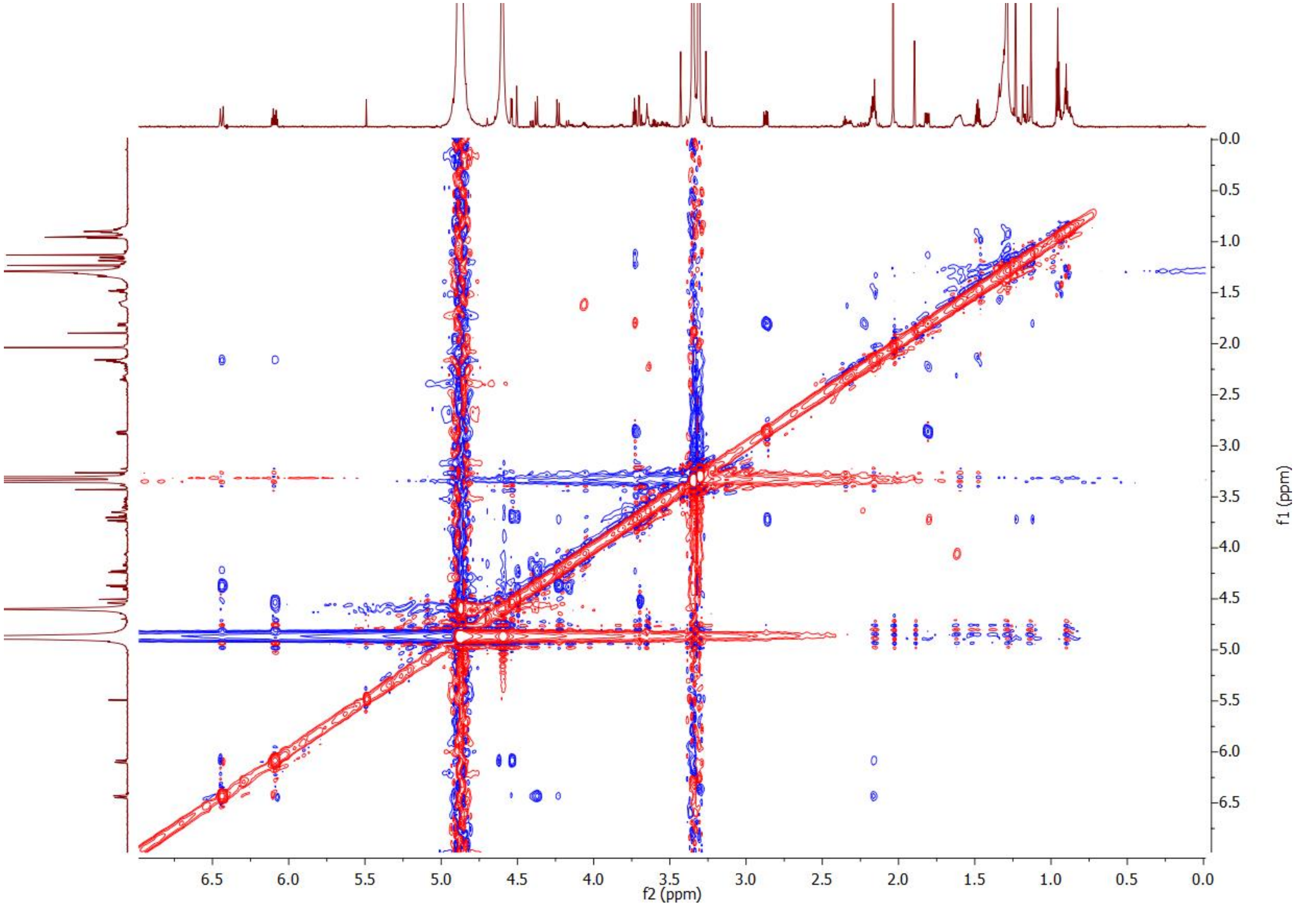

Figure S36. ^1H-^1H ROESY spectrum of compound **5**.

Table S16. Comparison of ^{1}H NMR data of compound **5** and calculated ^{1}H NMR data of 6 possible isomers of **5** in MeOH-d_4.

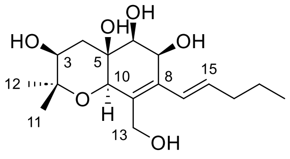

5_1

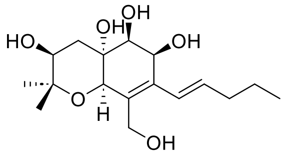

5_2

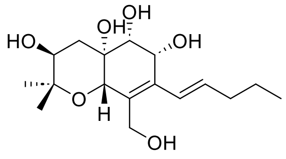

5_3

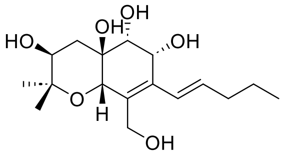

5_4

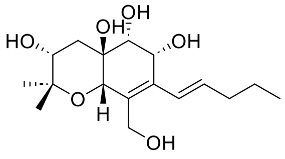

5_5

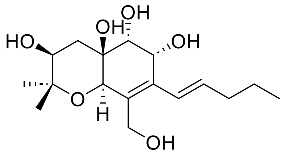

5_6

N°	Exp	5_1				5_2				5_3				5_4				5_5				5_6			
		unscaled	Δ	scaled	Δ	unscaled	Δ	scaled	Δ	unscaled	Δ	scaled	Δ	unscaled	Δ	scaled	Δ	unscaled	Δ	scaled	Δ	unscaled	Δ	scaled	Δ
3	**3,73**	3,70	0,03	3,65	0,08	4,08	0,35	3,94	0,21	4,11	0,38	4,11	0,38	4,35	0,62	4,27	0,54	3,49	0,24	3,46	0,27	3,93	0,20	3,85	0,12
4	**2,87**	2,14	0,73	2,17	0,70	2,43	0,44	2,42	0,45	1,92	0,95	1,98	0,89	2,03	0,84	1,98	0,89	2,32	0,55	2,27	0,60	1,74	1,13	1,72	1,15
	**1,81**	1,82	0,01	1,87	0,06	1,71	0,10	1,76	0,05	1,69	0,12	1,76	0,05	2,02	0,21	1,97	0,16	2,12	0,31	2,07	0,26	2,37	0,56	2,33	0,52
6	**3,70**	3,60	0,10	3,55	0,15	3,64	0,06	3,54	0,16	3,85	0,15	3,85	0,15	3,67	0,03	3,60	0,10	3,88	0,18	3,86	0,16	3,76	0,06	3,68	0,02
7	**4,54**	4,85	0,31	4,73	0,19	4,55	0,01	4,37	0,17	4,22	0,32	4,21	0,33	4,42	0,12	4,34	0,20	4,61	0,07	4,60	0,06	4,50	0,04	4,40	0,14
10	**4,50**	4,12	0,38	4,04	0,46	4,36	0,14	4,20	0,30	4,14	0,36	4,13	0,37	4,72	0,22	4,63	0,13	4,62	0,12	4,61	0,11	4,88	0,38	4,76	0,26
11	**1,23**	1,16	0,07	1,25	0,02	0,97	0,26	1,08	0,15	1,23	0,00	1,31	0,08	1,32	0,09	1,28	0,05	1,31	0,08	1,25	0,02	1,28	0,05	1,27	0,04
12	**1,13**	1,24	0,11	1,33	0,20	1,25	0,12	1,34	0,21	1,38	0,25	1,46	0,33	1,25	0,12	1,21	0,08	1,18	0,05	1,12	0,01	1,23	0,10	1,22	0,09
13	**4,38**	4,51	0,13	4,41	0,03	4,83	0,45	4,63	0,25	4,51	0,13	4,49	0,11	4,72	0,34	4,64	0,26	4,69	0,31	4,68	0,30	4,71	0,33	4,60	0,22
	**4,23**	4,50	0,27	4,40	0,17	4,55	0,32	4,37	0,14	3,93	0,30	3,94	0,29	4,48	0,25	4,40	0,17	4,50	0,27	4,49	0,26	4,16	0,07	4,07	0,16
14	**6,44**	6,79	0,35	6,56	0,12	6,89	0,45	6,52	0,08	6,52	0,08	6,45	0,01	6,41	0,03	6,31	0,13	6,18	0,26	6,19	0,25	6,34	0,10	6,19	0,25
15	**6,09**	6,57	0,48	6,35	0,26	6,53	0,44	6,19	0,10	6,76	0,67	6,68	0,59	6,16	0,07	6,06	0,03	6,08	0,01	6,10	0,01	6,68	0,59	6,51	0,42
16	**2,16**	2,24	0,08	2,26	0,10	2,23	0,07	2,24	0,08	2,18	0,02	2,23	0,07	2,21	0,05	2,16	0,00	2,29	0,13	2,24	0,08	2,20	0,04	2,16	0,00
17	**1,48**	1,48	0,00	1,56	0,08	1,44	0,04	1,51	0,03	1,46	0,02	1,54	0,06	1,49	0,01	1,45	0,03	1,53	0,05	1,46	0,02	1,48	0,00	1,47	0,01
18	**0,96**	1,02	0,06	1,12	0,16	1,01	0,05	1,12	0,16	1,00	0,04	1,09	0,13	0,98	0,02	0,95	0,01	0,93	0,03	0,86	0,10	1,01	0,05	1,00	0,04
**R^2**		**0,98935**		**0,98934**		**0,99340**		**0,99340**		**0,98049**		**0,98049**		**0,98561**		**0,98561**		**0,99133**		**0,99133**		**0,97796**		**0,97796**	
**MAE**			**0,21**		**0,19**		**0,22**		**0,17**		**0,25**		**0,26**		**0,20**		**0,19**		**0,18**		**0,17**		**0,25**		**0,23**

Table S17. Comparison of ^{13}C NMR data of compound **5** and calculated ^{13}C NMR data of 6 possible isomers of **5** in MeOH-d_4.

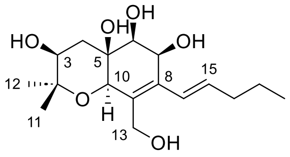

5_1

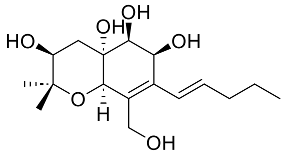

5_2

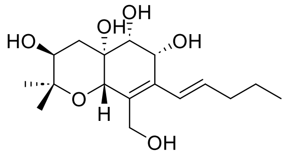

5_3

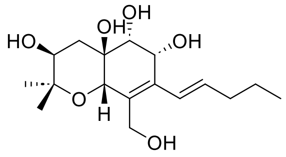

5_4

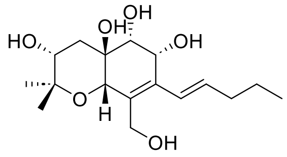

5_5

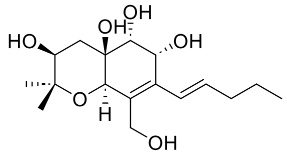

5_6

N°	Exp	5_1				5_2				5_3				5_4				5_5				5_6			
		unscaled	Δ	scaled	Δ	unscaled	Δ	scaled	Δ	unscaled	Δ	scaled	Δ	unscaled	Δ	scaled	Δ	unscaled	Δ	scaled	Δ	unscaled	Δ	scaled	Δ
2	**85,3**	80,6	4,7	80,9	4,4	80,1	5,2	79,6	5,7	80,4	4,9	80,5	4,8	80,9	4,4	80,6	4,7	80,7	4,6	80,9	4,4	79,6	5,7	79,4	5,9
3	**72**	74,4	2,4	74,5	2,5	74,0	2,0	73,4	1,4	74,4	2,4	74,3	2,3	74,7	2,7	74,1	2,1	74,4	2,4	74,4	2,4	77,4	5,4	77,1	5,1
4	**37,2**	36,9	0,3	36,1	1,1	36,8	0,4	35,7	1,5	44,4	7,2	43,4	6,2	46,8	9,6	45,6	8,4	45,0	7,8	44,4	7,2	36,6	0,6	35,9	1,3
5	**81,9**	75,6	6,3	75,8	6,1	78,0	3,9	77,5	4,4	75,0	6,9	74,9	7,0	72,4	9,5	71,9	10,0	70,8	11,1	70,7	11,2	76,3	5,6	76,0	5,9
6	**72,9**	75,0	2,1	75,1	2,2	78,0	5,1	77,4	4,5	79,6	6,7	79,6	6,7	80,3	7,4	79,9	7,0	74,7	1,8	74,8	1,9	78,4	5,5	78,1	5,2
7	**68,5**	72,3	3,8	72,3	3,8	71,3	2,8	70,6	2,1	71,5	3,0	71,3	2,8	72,8	4,3	72,2	3,7	71,9	3,4	71,9	3,4	72,3	3,8	72,0	3,5
8	**135,1**	135,4	0,3	137,1	2,0	134,7	0,4	135,0	0,1	138,8	3,7	140,6	5,5	142,2	7,1	143,4	8,3	137,9	2,8	139,3	4,2	142,3	7,2	142,7	7,6
9	**134,9**	137,2	2,3	138,9	4,0	139,9	5,0	140,2	5,3	134,2	0,7	135,9	1,0	130,0	4,9	130,9	4,0	133,8	1,1	135,2	0,3	134,0	0,9	134,3	0,6
10	**86,2**	78,2	8,0	78,4	7,8	80,3	5,9	79,8	6,4	74,5	11,7	74,4	11,8	72,3	13,9	71,7	14,5	71,4	14,8	71,4	14,8	72,4	13,8	72,0	14,2
11	**25,6**	22,8	2,8	21,6	4,0	19,1	6,5	17,8	7,8	16,2	9,4	14,4	11,2	16,8	8,8	14,8	10,8	26,9	0,4	25,8	0,7	25,5	1,0	24,7	1,8
12	**26,5**	25,7	0,8	24,6	1,9	29,9	3,4	28,7	2,2	29,1	2,6	27,7	1,2	28,6	2,1	26,9	0,4	22,6	3,0	21,5	4,1	22,1	3,5	21,3	4,3
13	**58,1**	59,8	1,7	59,5	1,4	60,1	2,0	59,3	1,2	58,4	0,3	57,9	0,2	62,3	4,2	61,5	3,4	62,0	3,9	61,8	3,7	61,0	2,9	60,6	2,5
14	**126,7**	123,1	3,6	124,4	2,3	124,4	2,3	124,5	2,2	121,1	5,6	122,4	4,3	124,2	2,5	124,9	1,8	120,8	5,9	121,8	4,9	125,8	0,9	126,0	0,7
15	**135,3**	135,6	0,3	137,3	2,0	136,9	1,6	137,2	1,9	138,1	2,8	139,8	4,5	139,9	4,6	141,0	5,7	144,9	9,6	146,5	11,2	136,1	0,8	136,5	1,2
16	**36,4**	41,7	5,3	41,0	4,6	41,4	5,0	40,4	4,0	41,6	5,2	40,5	4,1	40,9	4,5	39,6	3,2	40,0	3,6	39,2	2,8	40,8	4,4	40,1	3,7
17	**23,3**	28,7	5,4	27,6	4,3	28,9	5,6	27,7	4,4	29,2	5,9	27,8	4,5	28,6	5,3	26,9	3,6	27,1	3,8	26,1	2,8	28,7	5,4	27,9	4,6
18	**13,9**	16,0	2,1	14,7	0,8	16,3	2,4	14,9	1,0	16,3	2,4	14,5	0,6	16,1	2,2	14,1	0,2	15,4	1,5	14,2	0,3	16,0	2,1	15,1	1,2
**R^2**		**0,99582**		**0,99582**		**0,99535**		**0,99535**		**0,99046**		**0,99046**		**0,98706**		**0,98706**		**0,98847**		**0,98847**		**0,99177**		**0,99177**	
**MAE**			**3,06**		**3,25**		**3,50**		**3,31**		**4,78**		**4,64**		**5,75**		**5,39**		**4,79**		**4,71**		**4,09**		**4,07**

Functional	Solvent?	Basis Set	Type of Data
B3LYP	PCM	6-31+G(d,p)	Unscaled Shifts

	Isomer 1	Isomer 2	Isomer 3	Isomer 4	Isomer 5	Isomer 6
sDP4+ (H data)	7,93%	23,15%	0,00%	19,56%	49,22%	0,13%
sDP4+ (C data)	82,27%	17,73%	0,00%	0,00%	0,00%	0,00%
sDP4+ (all data)	61,38%	38,62%	0,00%	0,00%	0,00%	0,00%
uDP4+ (H data)	90,59%	8,18%	0,06%	0,63%	0,42%	0,13%
uDP4+ (C data)	17,06%	82,35%	0,01%	0,00%	0,00%	0,58%
uDP4+ (all data)	69,64%	30,36%	0,00%	0,00%	0,00%	0,00%
DP4+ (H data)	76,37%	20,15%	0,00%	1,30%	2,18%	0,00%
DP4+ (C data)	49,03%	50,97%	0,00%	0,00%	0,00%	0,00%
DP4+ (all data)	78,48%	21,52%	0,00%	0,00%	0,00%	0,00%

Figure S37. DP4+ probability analysis for the determination of the absolute configuration of **5**.

Single Mass Analysis
Tolerance = 5.0 mDa / DBE: min = -1.5, max = 50.0
Element prediction: Off
Number of isotope peaks used for i-FIT = 3
Monoisotopic Mass, Even Electron Ions
203 formula(e) evaluated with 1 results within limits (up to 50 closest results for each mass)
Elements Used:

Mass	Calc. Mass	mDa	PPM	DBE	Formula	i-FIT	i-FIT Norm	Fit Conf %	C
657.3855	657.3850	0.5	0.8	6.5	C34 H57 O12	148.0	n/a	n/a	34

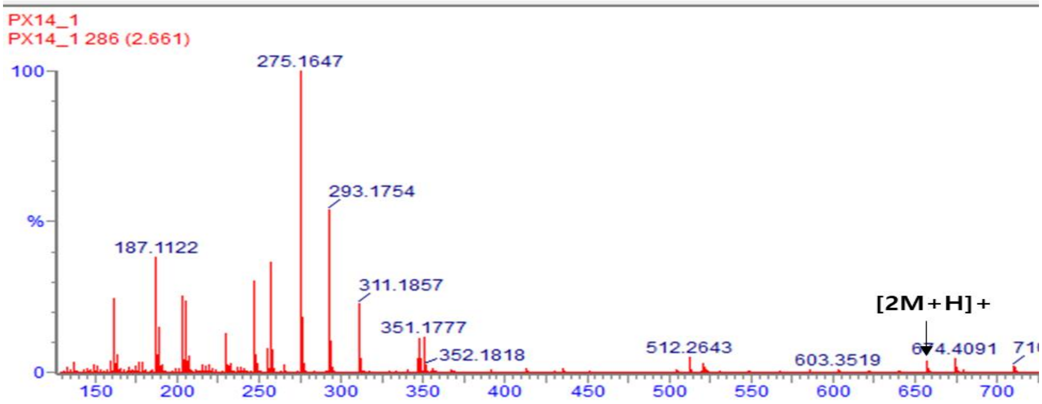

Figure S38. HR-ESIMS data of compound **6**.

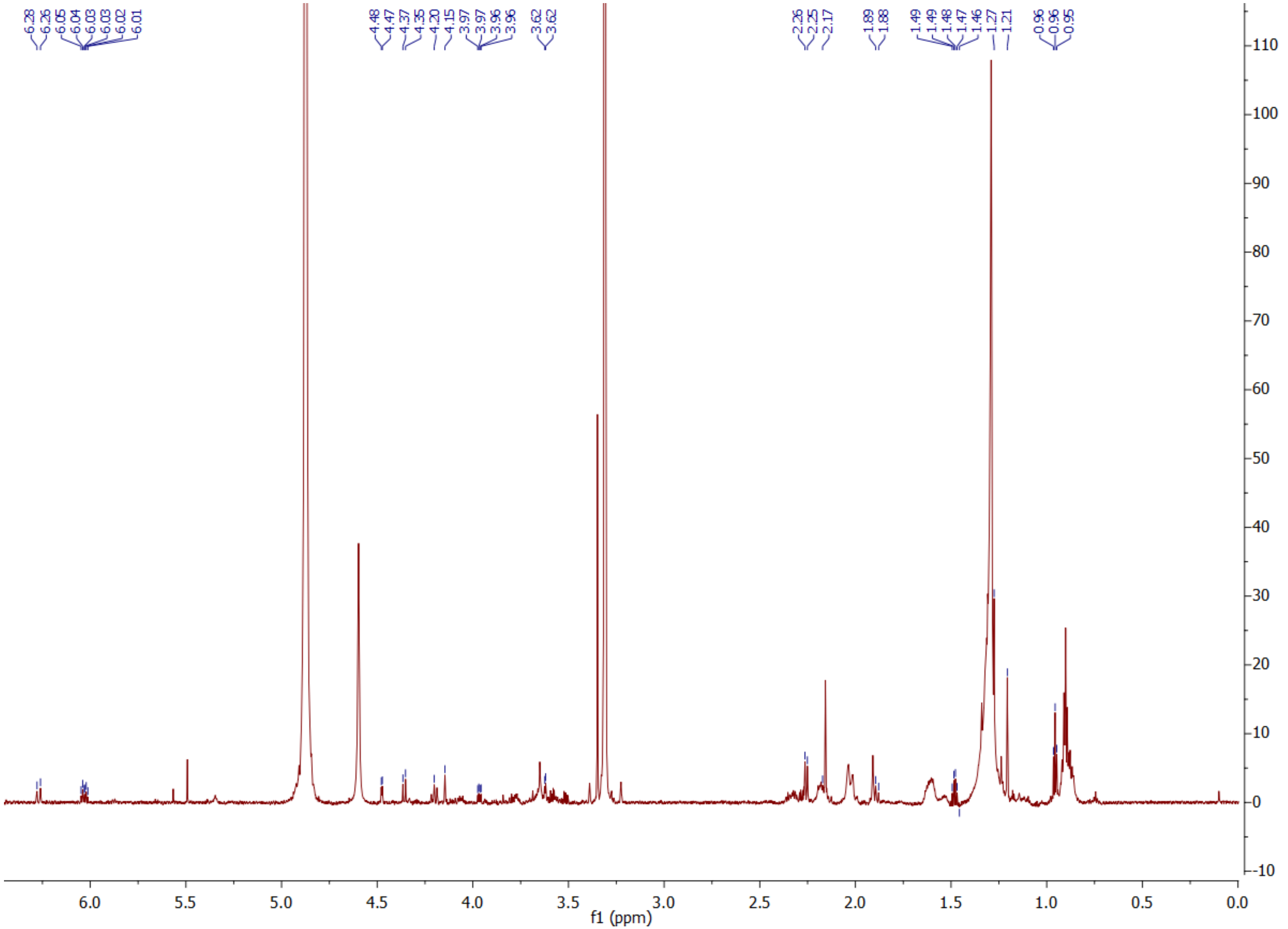

Figure S39. ^{1}H NMR spectrum of compound **6**.

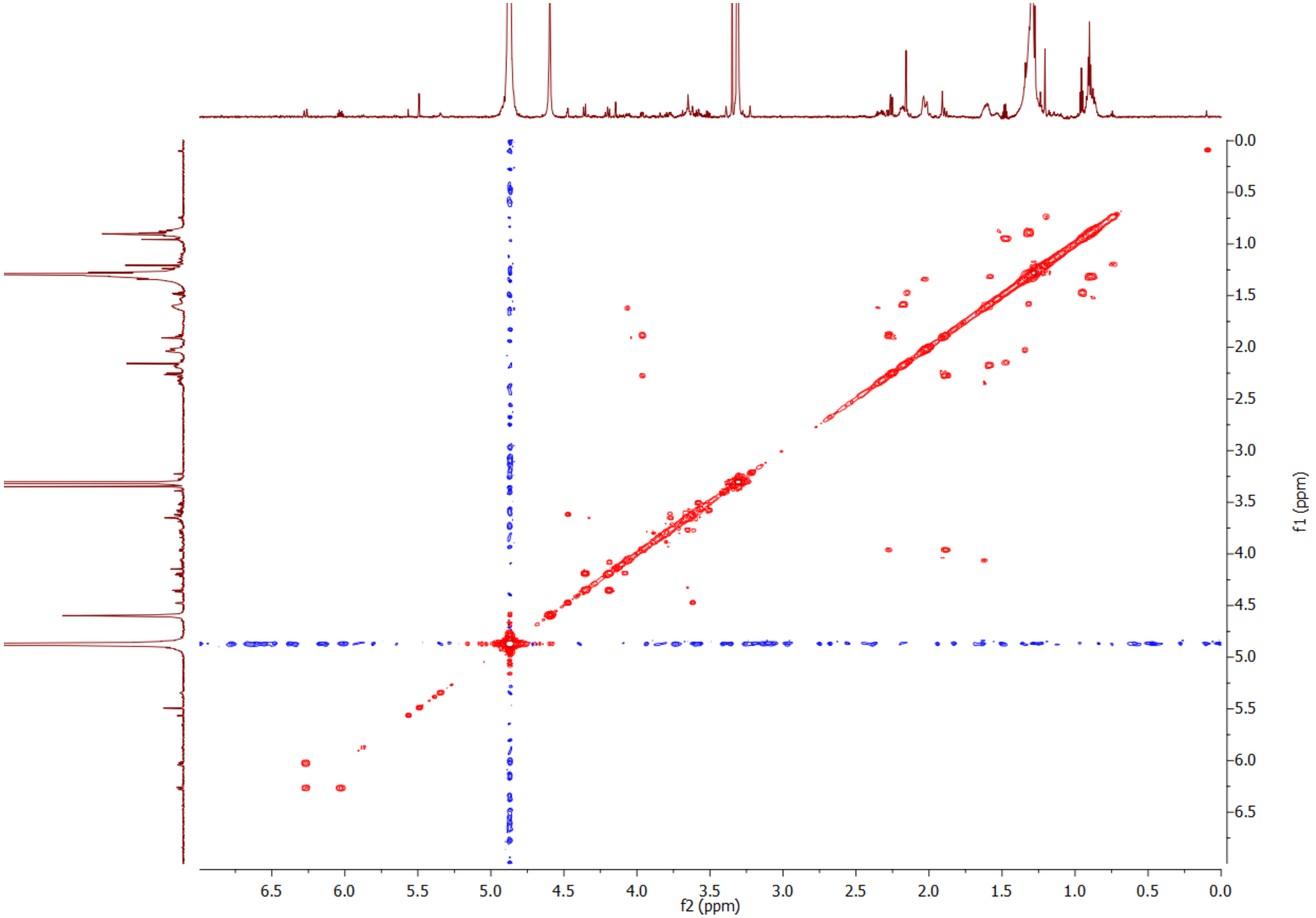

Figure S40. ^{1}H-^{1}H COSY spectrum of compound **6**.

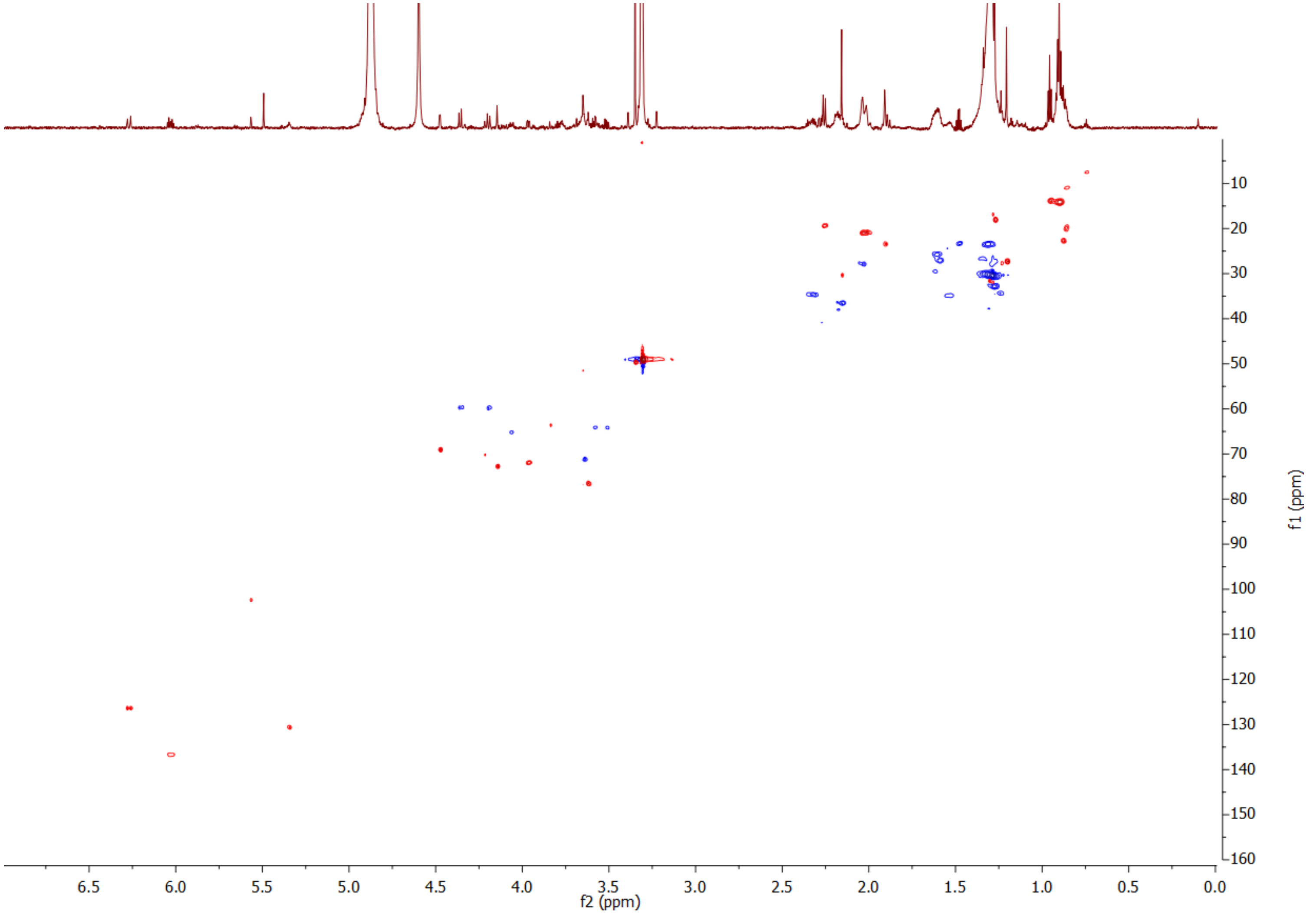

Figure S41. ^{1}H-^{13}C HSQC spectrum of compound **6**.

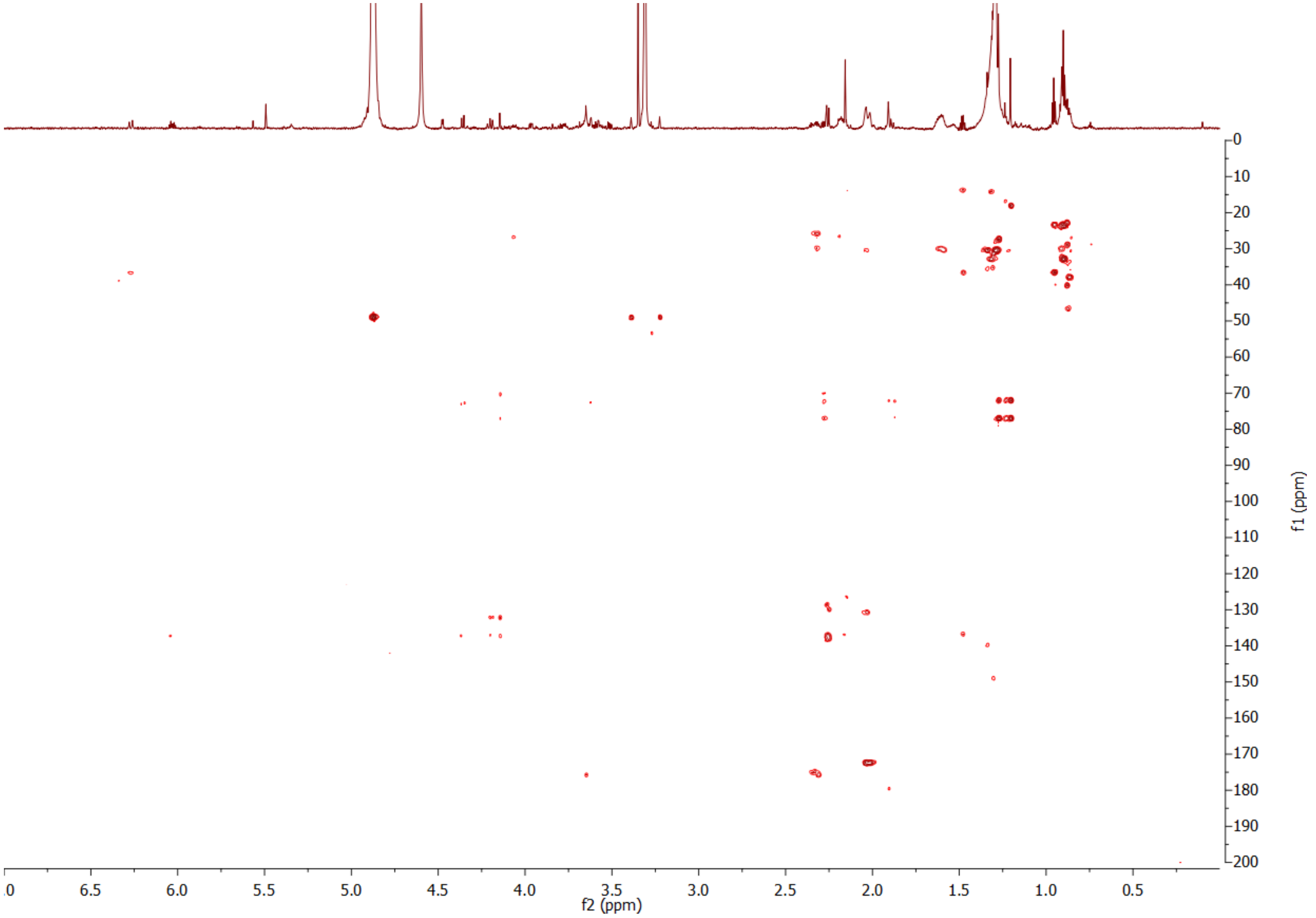

Figure S42. ^{1}H-^{13}C HMBC spectrum of compound **6**.

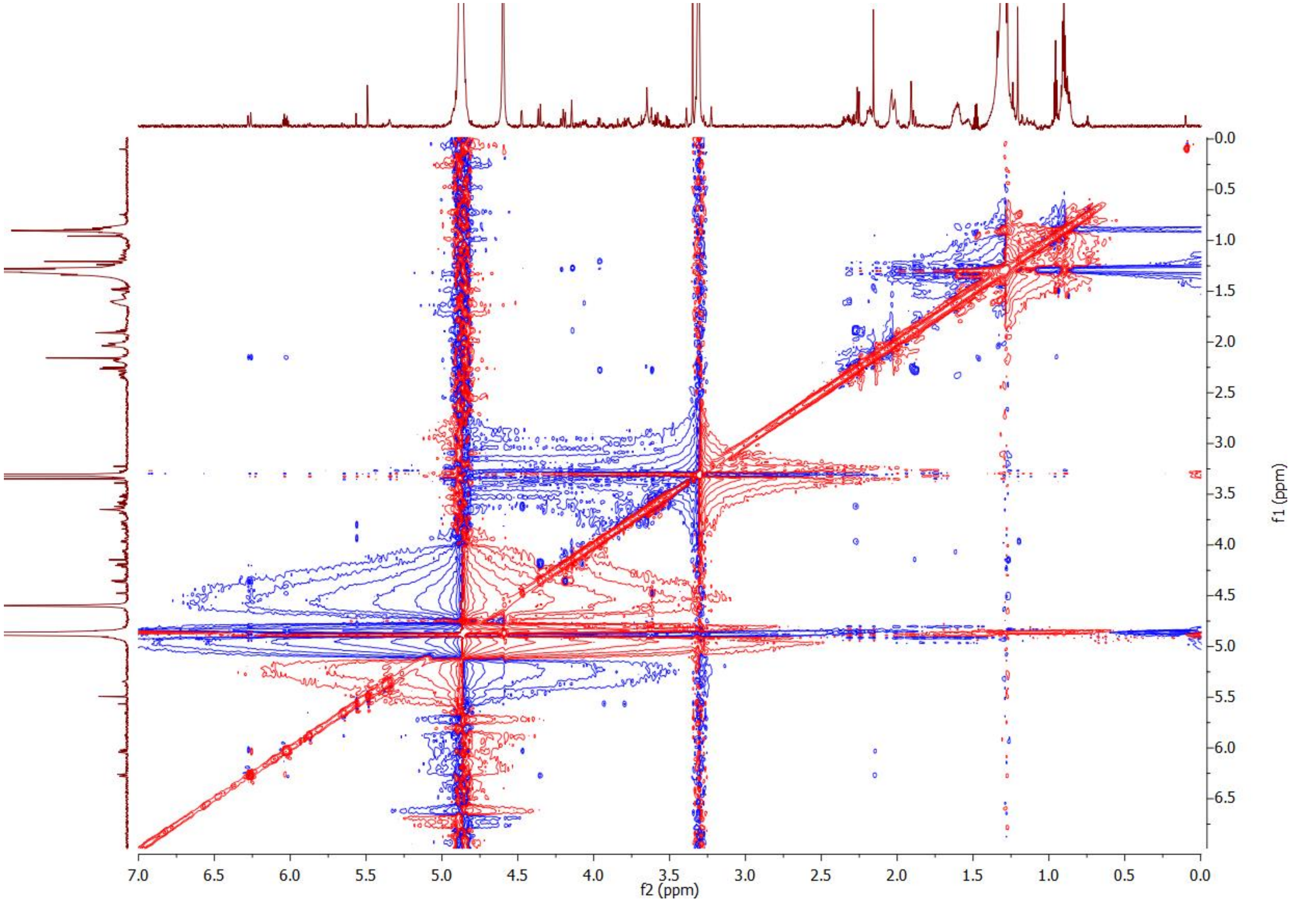

Figure S43. ^1H-^1H ROESY spectrum of compound **6**.

Table S18. Comparison of ^{1}H NMR data of compound **6** and calculated ^{1}H NMR data of 6 possible isomers of **6** in MeOH-d_4.

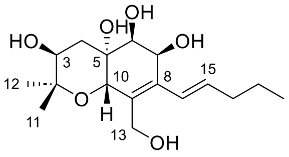

6_1

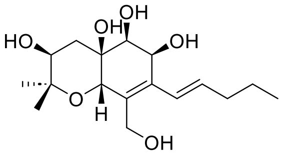

6_2

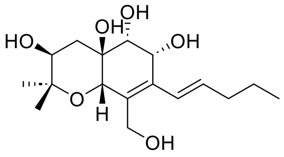

6_3

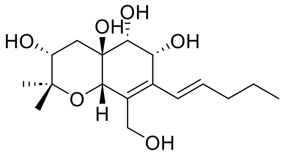

6_4

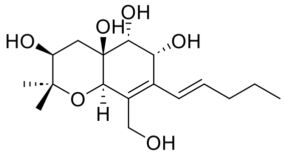

6_5

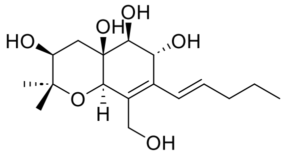

6_6

N°	Exp	6_1				6_2				6_3				6_4				6_5				6_6			
		unscaled	Δ	scaled	Δ	unscaled	Δ	scaled	Δ	unscaled	Δ	scaled	Δ	unscaled	Δ	scaled	Δ	unscaled	Δ	scaled	Δ	unscaled	Δ	scaled	Δ
3	**3,97**	4,14	0,17	4,02	0,05	3,69	0,28	3,49	0,48	4,35	0,38	4,18	0,21	3,49	0,48	3,39	0,58	3,93	0,04	3,77	0,20	3,88	0,09	3,79	0,18
4	**2,28**	1,99	0,29	1,98	0,30	2,10	0,18	2,07	0,21	2,03	0,25	1,96	0,32	2,32	0,04	2,23	0,05	1,74	0,54	1,71	0,57	2,32	0,04	2,29	0,01
	**1,88**	1,71	0,17	1,71	0,17	1,60	0,28	1,62	0,26	2,02	0,14	1,95	0,07	2,12	0,24	2,03	0,15	2,37	0,49	2,30	0,42	1,94	0,06	1,93	0,05
6	**3,62**	3,50	0,12	3,41	0,21	4,26	0,64	4,01	0,39	3,67	0,05	3,53	0,09	3,88	0,26	3,78	0,16	3,76	0,14	3,61	0,01	3,41	0,21	3,34	0,28
7	**4,48**	4,53	0,05	4,39	0,09	5,02	0,54	4,69	0,21	4,42	0,06	4,24	0,24	4,61	0,13	4,50	0,02	4,50	0,02	4,30	0,18	4,40	0,08	4,28	0,20
10	**4,15**	4,95	0,80	4,79	0,64	4,31	0,16	4,05	0,10	4,72	0,57	4,53	0,38	4,62	0,47	4,52	0,37	4,88	0,73	4,65	0,50	4,53	0,38	4,41	0,26
11	**1,27**	1,38	0,11	1,25	0,02	1,25	0,02	1,30	0,03	1,32	0,05	1,29	0,02	1,31	0,04	1,23	0,04	1,28	0,01	1,28	0,01	1,24	0,03	1,26	0,01
12	**1,21**	1,22	0,01	1,40	0,19	1,18	0,03	1,24	0,03	1,25	0,04	1,21	0,00	1,18	0,03	1,11	0,10	1,23	0,02	1,23	0,02	1,23	0,02	1,25	0,04
13	**4,36**	4,54	0,18	4,40	0,04	4,55	0,19	4,27	0,09	4,72	0,36	4,53	0,17	4,69	0,33	4,59	0,23	4,71	0,35	4,49	0,13	4,67	0,31	4,55	0,19
	**4,19**	4,30	0,11	4,17	0,02	4,29	0,10	4,04	0,15	4,48	0,29	4,31	0,12	4,50	0,31	4,40	0,21	4,16	0,03	3,98	0,21	4,10	0,09	4,00	0,19
14	**6,27**	6,32	0,05	6,10	0,17	6,70	0,43	6,20	0,07	6,41	0,14	6,15	0,12	6,18	0,09	6,06	0,21	6,34	0,07	6,03	0,24	6,29	0,02	6,09	0,18
15	**6,03**	6,25	0,22	6,03	0,00	6,81	0,78	6,30	0,27	6,16	0,13	5,90	0,13	6,08	0,05	5,96	0,07	6,68	0,65	6,34	0,31	6,62	0,59	6,41	0,38
16	**2,17**	2,22	0,05	2,19	0,02	2,36	0,19	2,30	0,13	2,21	0,04	2,13	0,04	2,29	0,12	2,20	0,03	2,20	0,03	2,14	0,03	2,22	0,05	2,20	0,03
17	**1,48**	1,48	0,00	1,50	0,02	1,56	0,08	1,59	0,11	1,49	0,01	1,45	0,03	1,53	0,05	1,45	0,03	1,48	0,00	1,47	0,01	1,49	0,01	1,50	0,02
18	**0,96**	0,94	0,02	0,98	0,02	1,05	0,09	1,13	0,17	0,98	0,02	0,96	0,00	0,93	0,03	0,86	0,10	1,01	0,05	1,02	0,06	0,97	0,01	1,00	0,04
**R^2**		**0,99262**		**0,99242**		**0,99168**		**0,99168**		**0,99491**		**0,99491**		**0,99192**		**0,99192**		**0,98782**		**0,98782**		**0,99446**		**0,99446**	
**MAE**			**0,16**		**0,13**		**0,27**		**0,18**		**0,17**		**0,13**		**0,18**		**0,16**		**0,21**		**0,19**		**0,13**		**0,14**

Table S19. Comparison of ^{13}C NMR data of compound **6** and calculated ^{13}C NMR data of 6 possible isomers of **6** in MeOH-d_4.

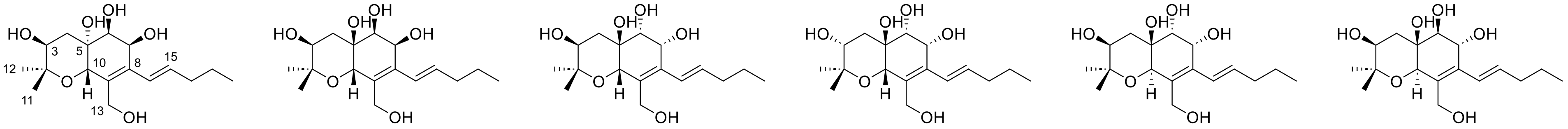

6_1 **6_2** **6_3** **6_4** **6_5** **6_6**

N°	Exp	6_1				6_2				6_3				6_4				6_5				6_6			
		unscaled	Δ	scaled	Δ	unscaled	Δ	scaled	Δ	unscaled	Δ	scaled	Δ	unscaled	Δ	scaled	Δ	unscaled	Δ	scaled	Δ	unscaled	Δ	scaled	Δ
2	**77,1**	80,8	3,7	79,3	2,2	79,7	2,6	79,3	2,2	80,9	3,8	79,5	2,4	80,7	3,6	79,8	2,7	79,6	2,5	78,3	1,2	79,8	2,7	78,1	1,0
3	**72**	74,9	2,9	73,3	1,3	74,6	2,6	74,1	2,1	74,7	2,7	73,1	1,1	74,4	2,4	73,3	1,3	77,4	5,4	76,0	4,0	76,2	4,2	74,5	2,5
4	**40,9**	41,7	0,8	39,7	1,2	39,7	1,2	38,6	2,3	46,8	5,9	44,7	3,8	45,0	4,1	43,5	2,6	36,6	4,3	35,0	5,9	35,6	5,3	33,8	7,1
5	**82**	80,0	2,0	78,5	3,5	77,0	5,0	76,5	5,5	72,4	9,6	70,8	11,2	70,8	11,2	69,7	12,3	76,3	5,7	75,0	7,0	75,3	6,7	73,6	8,4
6	**76,5**	79,6	3,1	78,1	1,6	70,8	5,7	70,2	6,3	80,3	3,8	78,8	2,3	74,7	1,8	73,7	2,8	78,4	1,9	77,1	0,6	79,6	3,1	78,0	1,5
7	**69,1**	71,6	2,5	69,9	0,8	71,9	2,8	71,3	2,2	72,8	3,7	71,2	2,1	71,9	2,8	70,8	1,7	72,3	3,2	71,0	1,9	79,1	10,0	77,4	8,3
8	**137,2**	141,5	4,3	140,7	3,5	138,7	1,5	139,3	2,1	142,2	5,0	141,8	4,6	137,9	0,7	138,0	0,8	142,3	5,1	141,4	4,2	140,5	3,3	139,1	1,9
9	**132,2**	134,4	2,2	133,5	1,3	133,9	1,7	134,4	2,2	130,0	2,2	129,4	2,8	133,8	1,6	133,9	1,7	134,0	1,8	133,1	0,9	135,1	2,9	133,6	1,4
10	**72,9**	74,5	1,6	72,9	0,0	78,3	5,4	77,9	5,0	72,3	0,6	70,7	2,2	71,4	1,5	70,3	2,6	72,4	0,5	71,0	1,9	76,0	3,1	74,3	1,4
11	**18,1**	16,4	1,7	14,1	4,0	16,5	1,6	15,0	3,1	16,8	1,3	14,2	3,9	22,6	4,5	20,7	2,6	22,1	4,0	20,4	2,3	21,9	3,8	20,0	1,9
12	**27,3**	29,3	2,0	27,2	0,1	29,1	1,8	27,8	0,5	28,6	1,3	26,2	1,1	26,9	0,4	25,0	2,3	25,5	1,8	23,8	3,5	25,4	1,9	23,4	3,9
13	**59,8**	61,7	1,9	60,0	0,2	61,2	1,4	60,4	0,6	62,3	2,5	60,5	0,7	62,0	2,2	60,8	1,0	61,0	1,2	59,6	0,2	60,9	1,1	59,1	0,7
14	**126,4**	123,7	2,7	122,7	3,7	123,0	3,4	123,3	3,1	124,2	2,2	123,5	2,9	120,8	5,6	120,6	5,8	125,8	0,6	124,8	1,6	124,0	2,4	122,6	3,8
15	**136,7**	135,4	1,3	134,5	2,2	135,2	1,5	135,7	1,0	139,9	3,2	139,4	2,7	144,9	8,2	145,1	8,4	136,1	0,6	135,2	1,5	137,8	1,1	136,4	0,3
16	**36,6**	40,4	3,8	38,4	1,8	39,7	3,1	38,5	1,9	40,9	4,3	38,7	2,1	40,0	3,4	38,3	1,7	40,8	4,2	39,2	2,6	40,3	3,7	38,4	1,8
17	**23,4**	28,0	4,6	25,9	2,5	28,3	4,9	27,0	3,6	28,6	5,2	26,2	2,8	27,1	3,7	25,3	1,9	28,7	5,3	27,0	3,6	28,0	4,6	26,1	2,7
18	**13,9**	15,6	1,7	13,3	0,6	14,2	0,3	12,6	1,3	16,1	2,2	13,4	0,5	15,4	1,5	13,4	0,5	16,0	2,1	14,3	0,4	15,6	1,7	13,7	0,2
**R^2**		0,99857		0,99857		0,99715		0,99715		0,99584		0,99584		0,99448		0,99448		0,99700		0,99700		0,99554		0,99554	
**MAE**			2,51		1,78		2,74		2,64		3,49		2,89		3,49		3,10		2,95		2,55		3,62		2,88

Functional	Solvent?	Basis Set	Type of Data
B3LYP	PCM	6-31+G(d,p)	Unscaled Shifts

	Isomer 1	Isomer 2	Isomer 3	Isomer 4	Isomer 5	Isomer 6
sDP4+ (H data)	63,93%	0,02%	28,09%	0,90%	0,00%	7,06%
sDP4+ (C data)	99,99%	0,00%	0,00%	0,00%	0,01%	0,00%
sDP4+ (all data)	100,00%	0,00%	0,00%	0,00%	0,00%	0,00%
uDP4+ (H data)	22,77%	0,00%	2,63%	0,02%	0,07%	74,51%
uDP4+ (C data)	99,37%	0,01%	0,60%	0,00%	0,02%	0,00%
uDP4+ (all data)	99,92%	0,00%	0,07%	0,00%	0,00%	0,01%
DP4+ (H data)	70,81%	0,00%	3,60%	0,00%	0,00%	25,59%
DP4+ (C data)	100,00%	0,00%	0,00%	0,00%	0,00%	0,00%
DP4+ (all data)	100,00%	0,00%	0,00%	0,00%	0,00%	0,00%

Figure S44. DP4+ probability analysis for the determination of the absolute configuration of **6**.

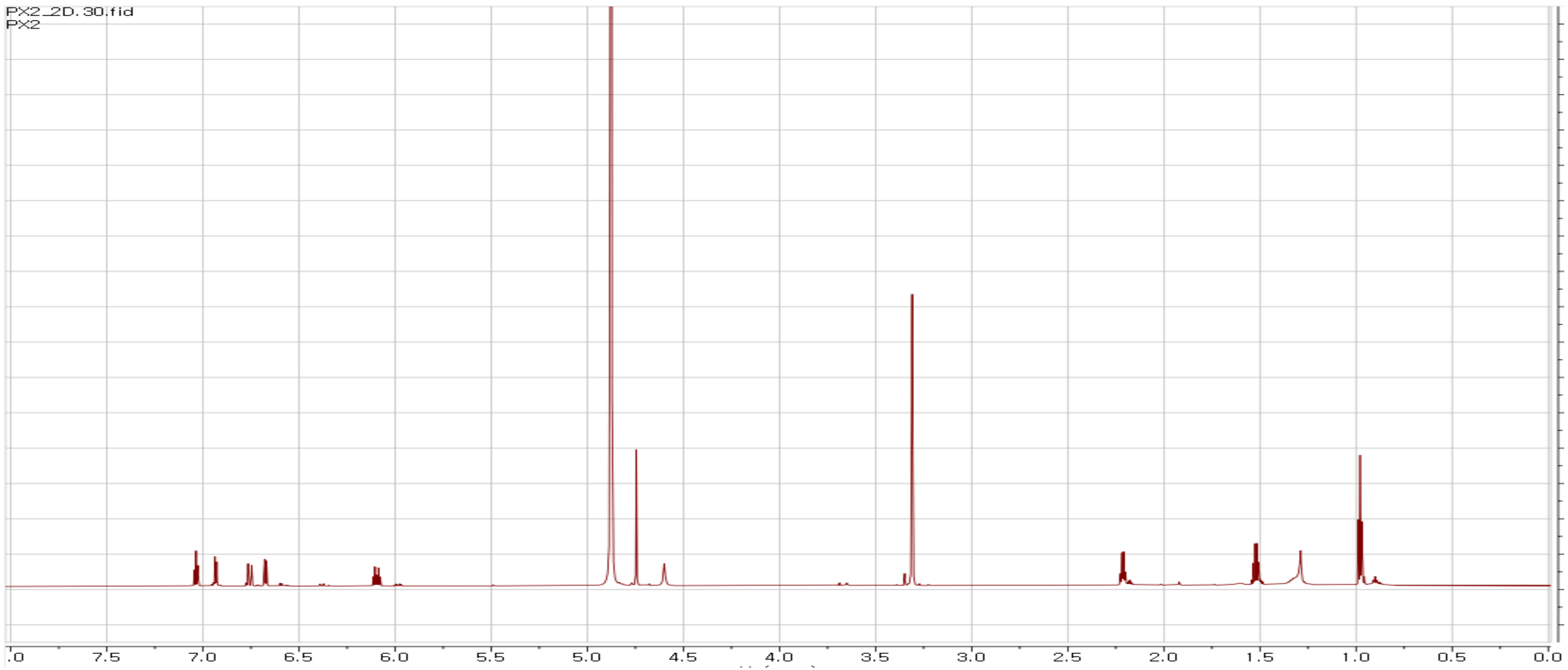

Figure S45. ^{1}H NMR spectrum of compound **7**.

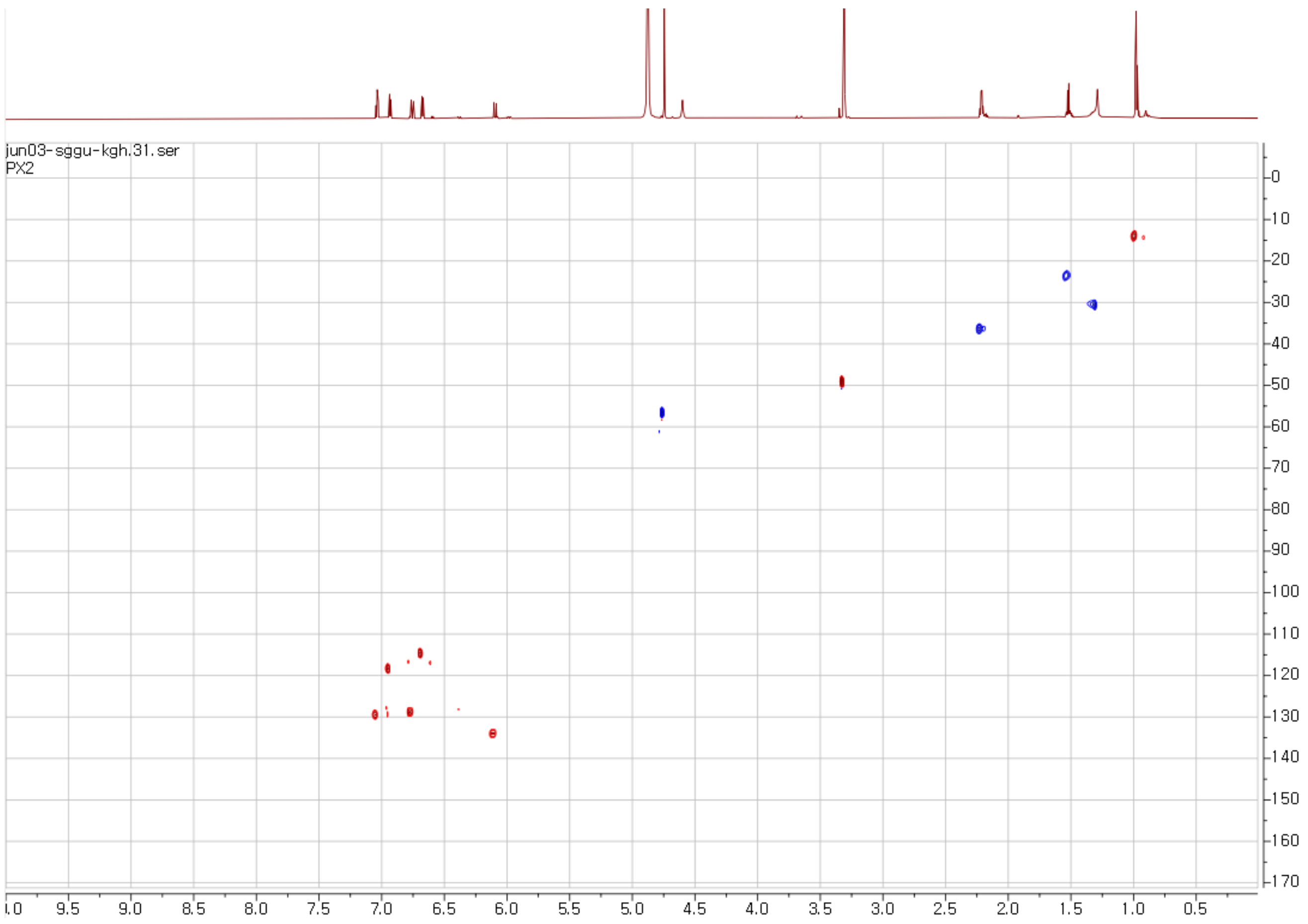

Figure S46. ^{1}H-^{13}C HSQC spectrum of compound **7**.

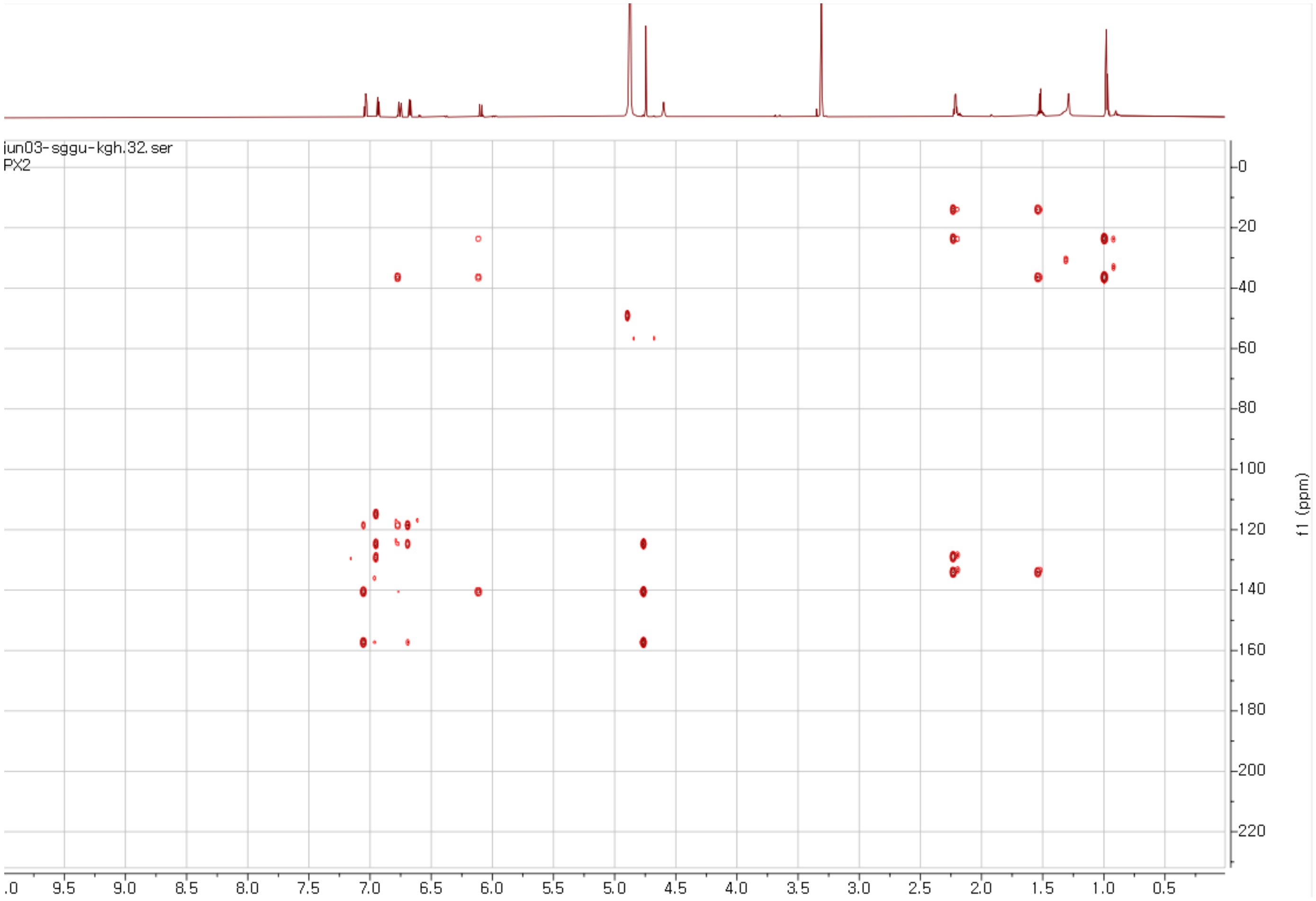

Figure S47. ^{1}H-^{13}C HMBC spectrum of compound **7**.

Table S20. ^{1}H and ^{13}C NMR (700 MHz) data of xylasporin I (**8**) in $CDCl_3$.[a]

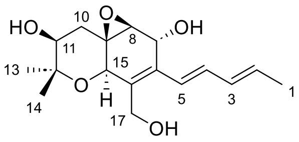

N°	δ_C	δ_H
1	18.6	1.81 d (6.7)
2	132.5	5.85 dq (13.8, 6.8)
3	132.2	6.15 m
4	124.4	6.37 d (15.5)
5	133.8	6.63 dd (15.5, 10.4)
6	132.0	
7	66.6	4.74 d (5.2)
8	70.3	3.94 d (5.2)
9	80.2	
10*α*	35.1	3.07 dd (14.3, 9.8)
10*β*		1.93 dd (14.3, 4.0)
11	84.6	3.88 dd (9.8, 4.0)
12	72.3	
13	27.4	1.36 s
14	26.2	1.14 s
15	87.1	4.60 s
16	134.7	
17	60.3	4.46 d (13.2),<br>4.37 d (13.2)

[a] Coupling constants (in parentheses) are in Hz.

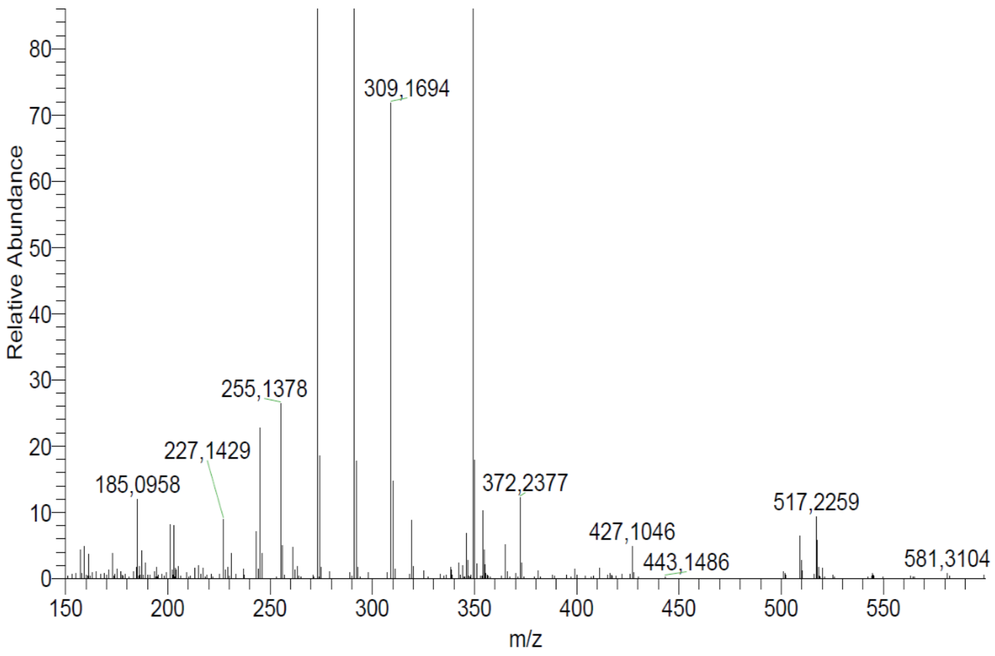

Figure S48. HR-ESIMS data of compound **8**.

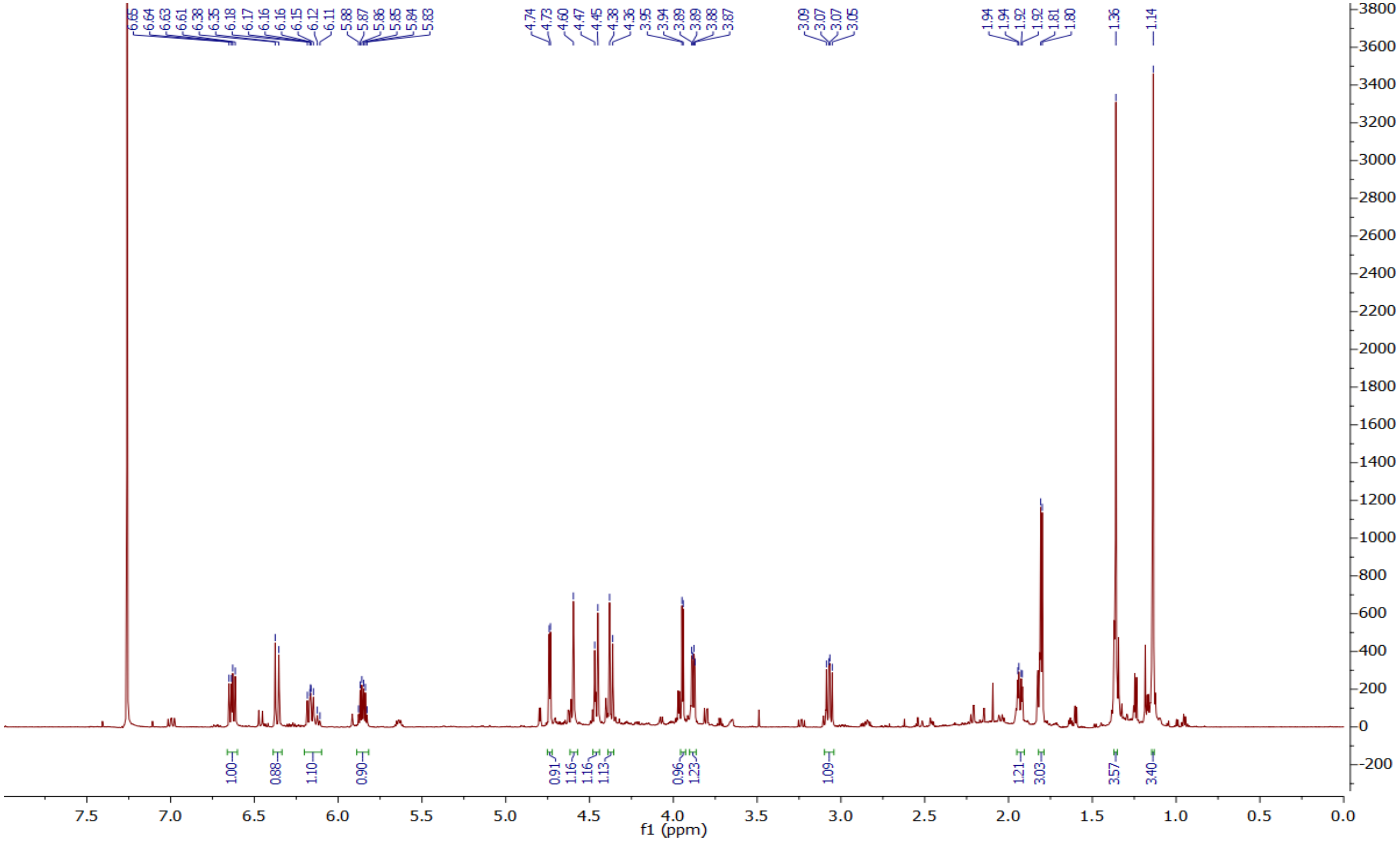

Figure S49. ^{1}H NMR spectrum of compound **8**.

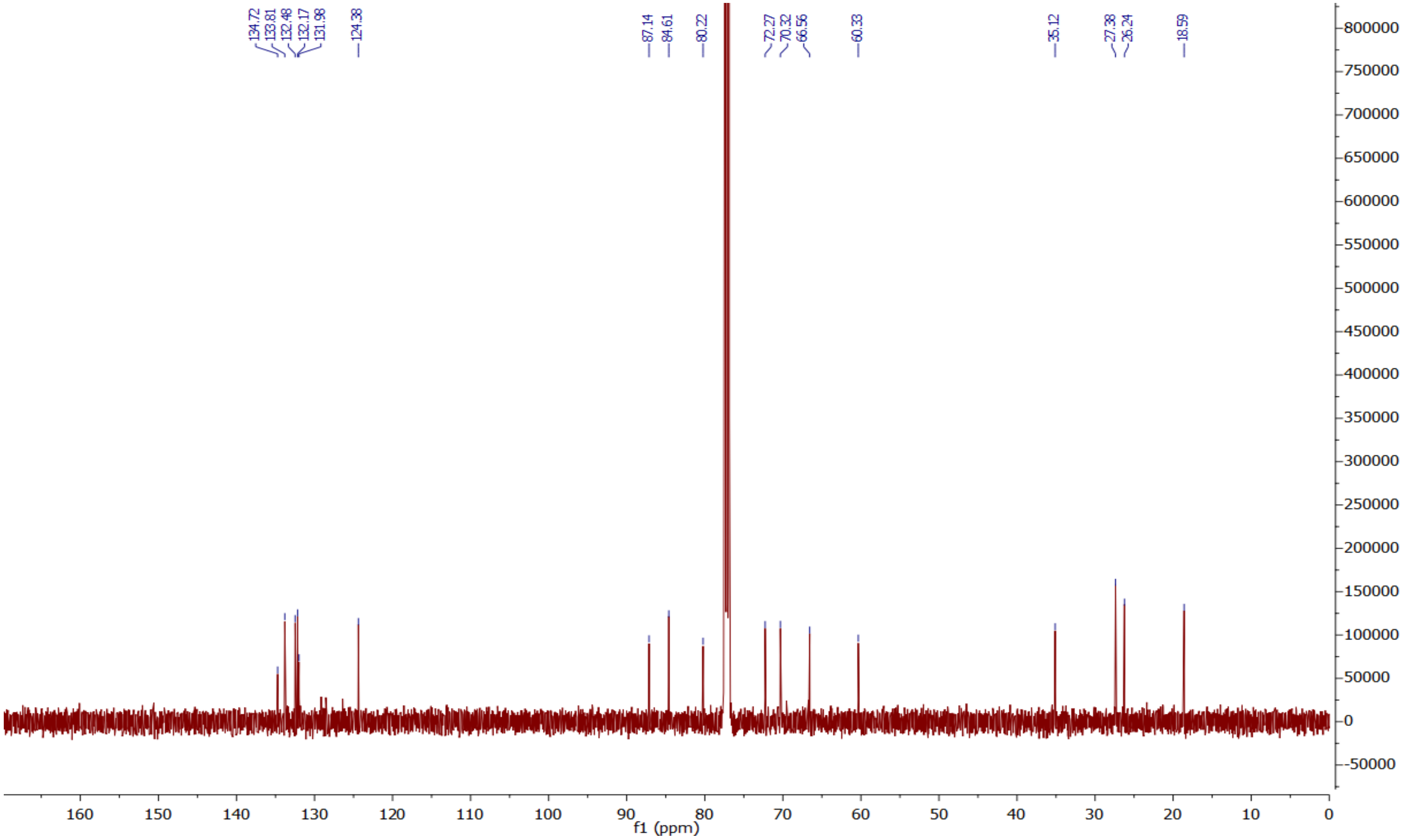

Figure S50. ^{13}C NMR spectrum of compound **8**.

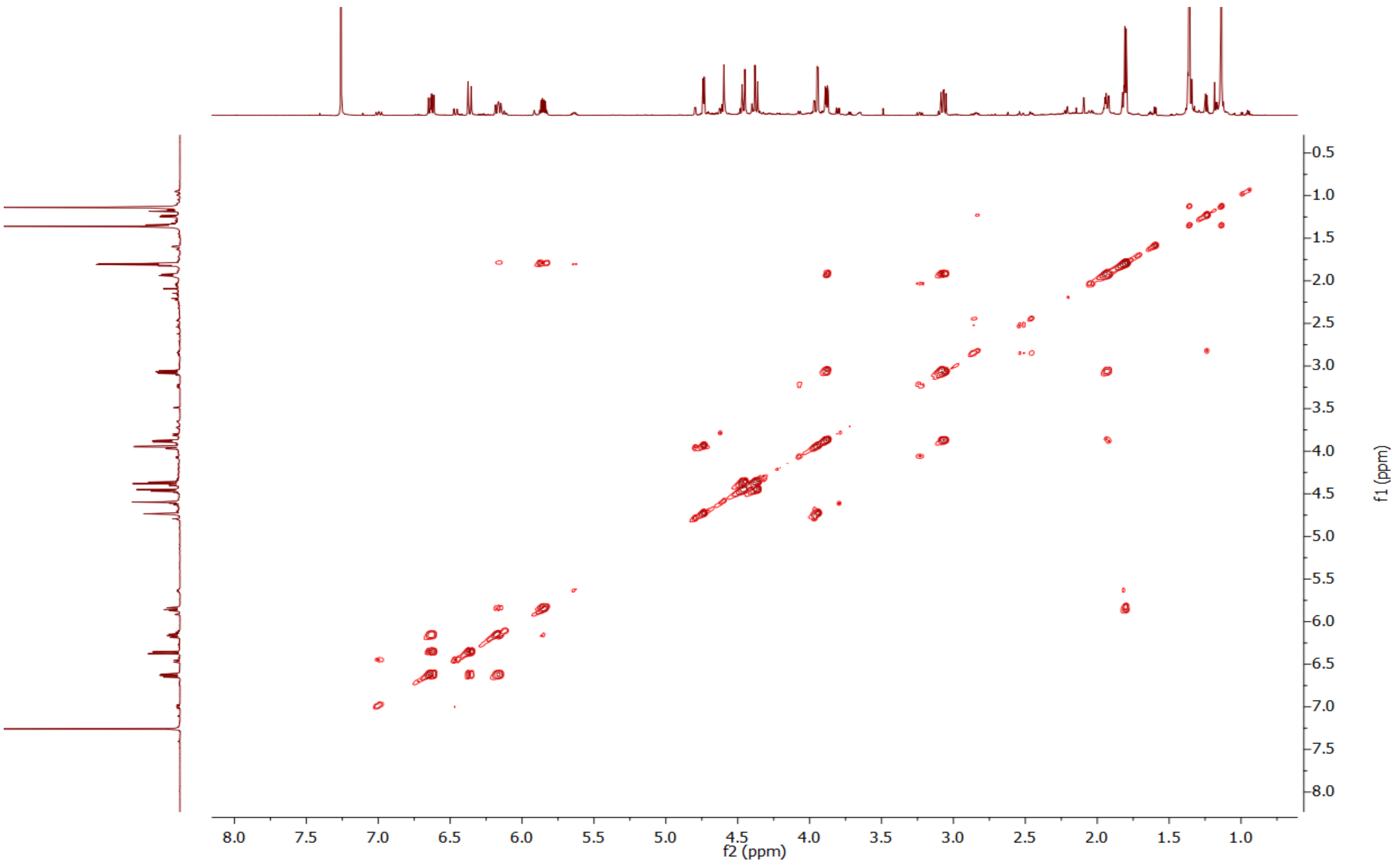

Figure S51. ^1H-^1H COSY spectrum of compound **8**.

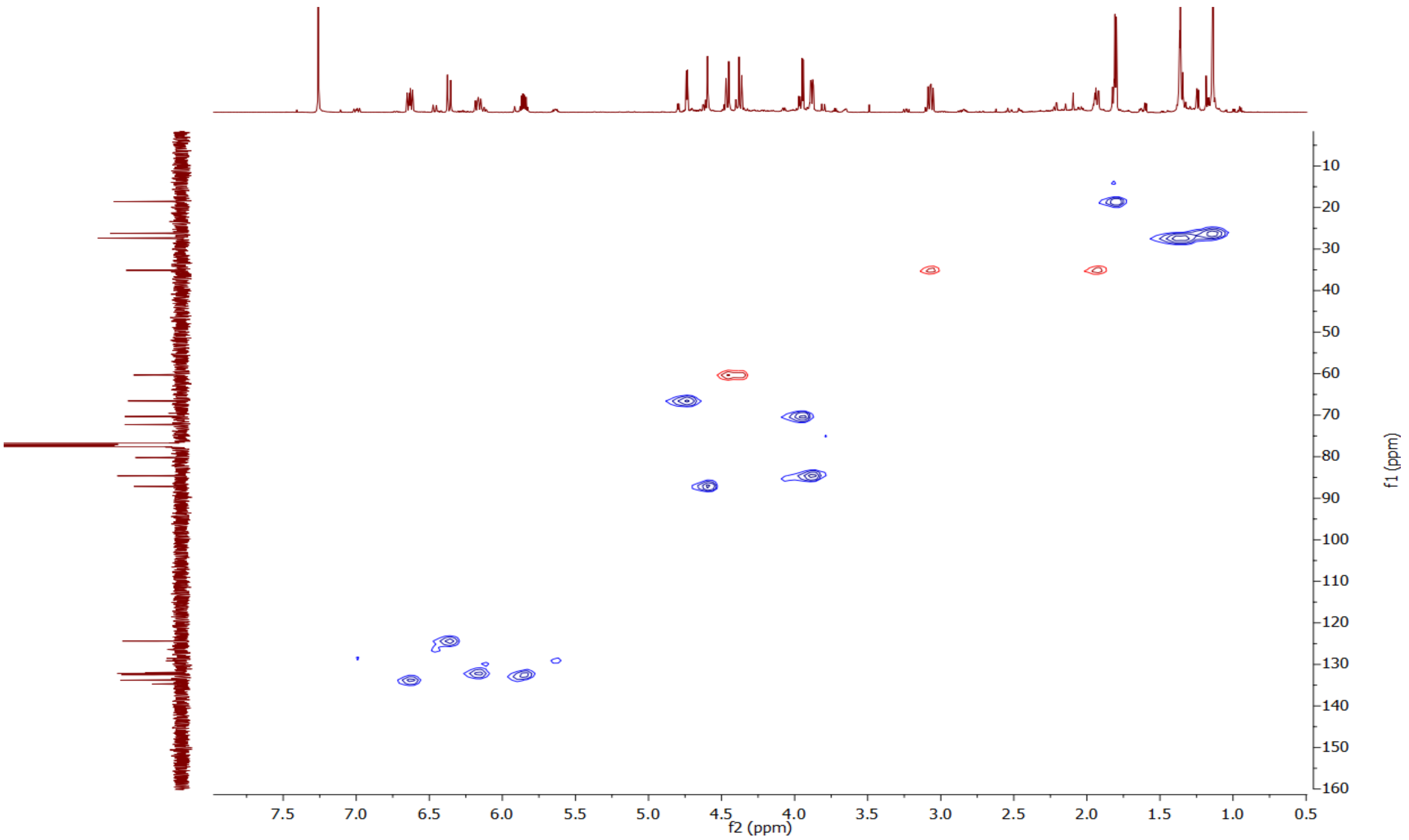

Figure S52. ^{1}H-^{13}C HSQC spectrum of compound **8**.

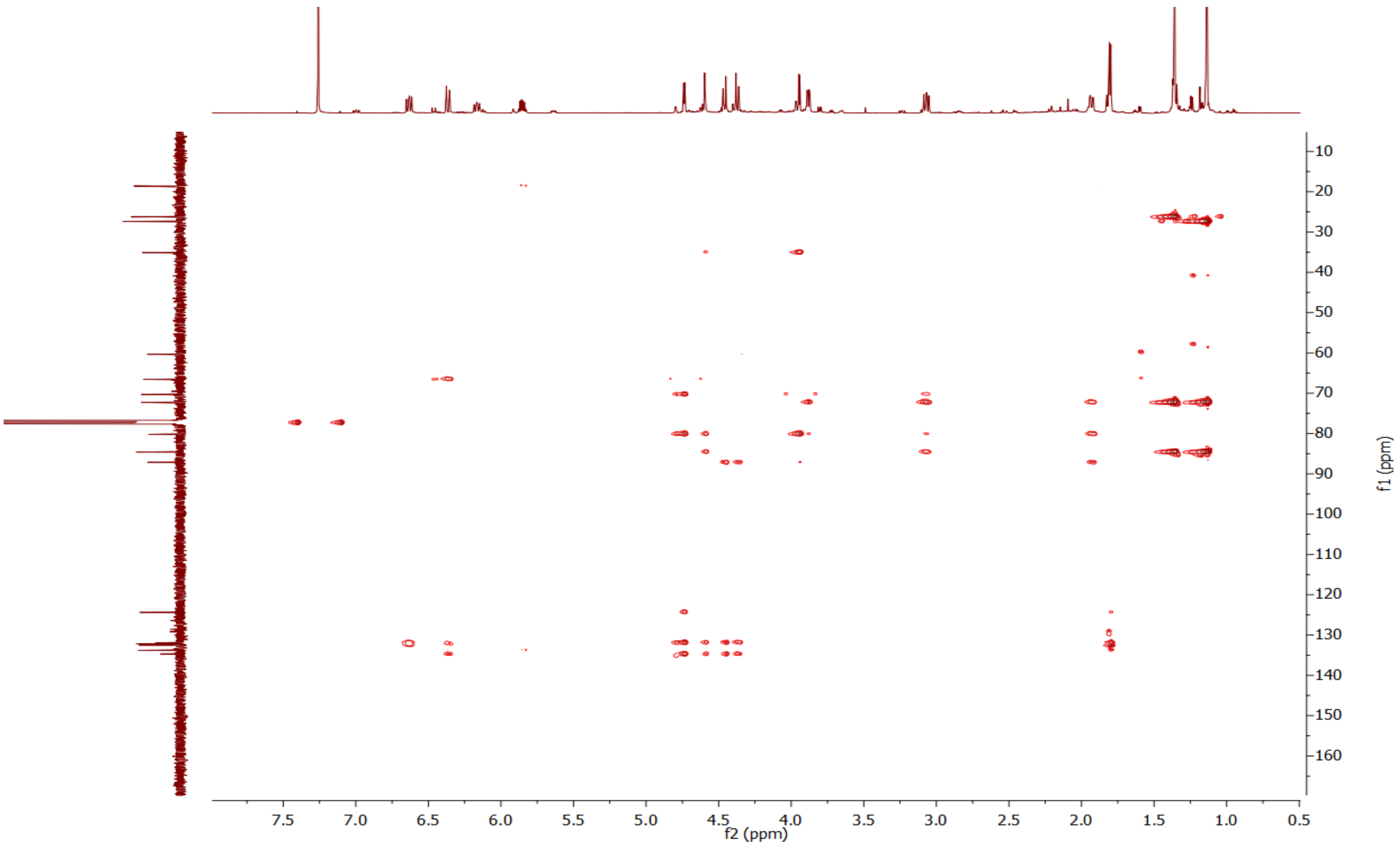

Figure S53. ^{1}H-^{13}C HMBC spectrum of compound **8**.

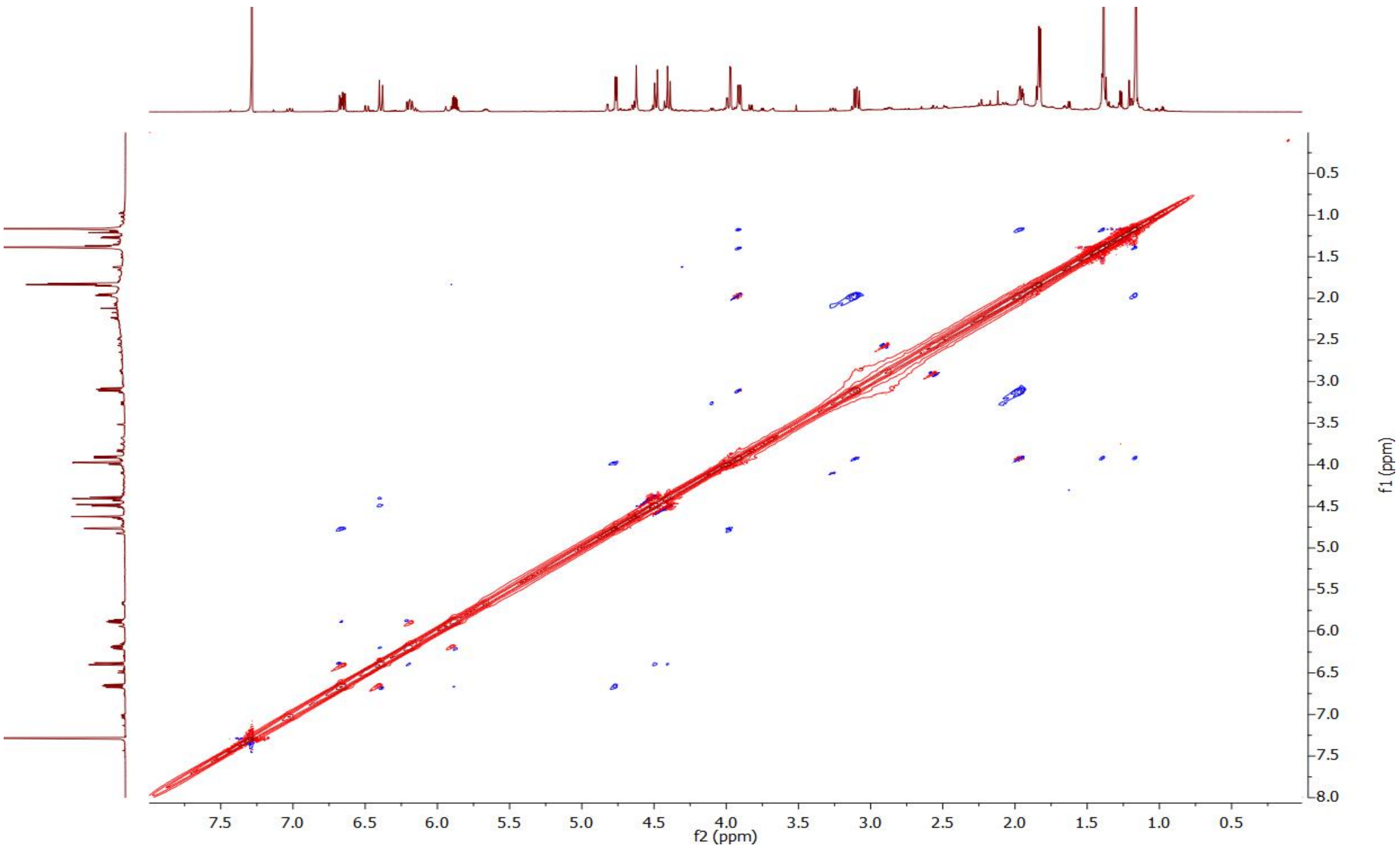

Figure S54. ^{1}H-^{1}H ROESY spectrum of compound **8**.

Table S21. Comparison of ^{1}H NMR data of compound **8** and calculated ^{1}H NMR data of 10 possible isomers of **8** in $CDCl_3$.

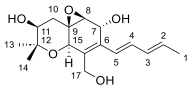

8_1

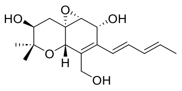

8_2

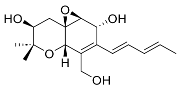

8_3

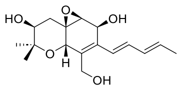

8_4

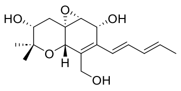

8_5

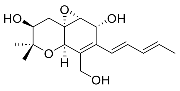

8_6

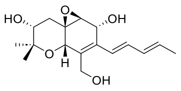

8_7

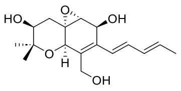

8_8

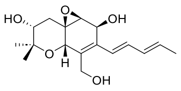

8_9

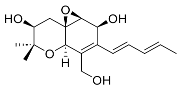

8_10

N°	Exp	8_1				8_2				8_3				8_4				8_5			
		unscaled	Δ	scaled	Δ	unscaled	Δ	scaled	Δ	unscaled	Δ	scaled	Δ	unscaled	Δ	scaled	Δ	unscaled	Δ	scaled	Δ
1	**1,81**	1,92	0,11	2,06	0,25	1,88	0,07	2,10	0,29	1,93	0,12	2,16	0,35	1,89	0,08	2,12	0,31	1,90	0,09	2,09	0,28
2	**5,85**	6,29	0,44	6,16	0,31	6,25	0,40	6,19	0,34	6,36	0,51	6,20	0,35	6,33	0,48	6,15	0,30	6,29	0,44	6,20	0,35
3	**6,15**	6,55	0,40	6,41	0,26	6,42	0,27	6,35	0,20	6,53	0,38	6,35	0,20	6,50	0,35	6,30	0,15	6,49	0,34	6,39	0,24
4	**6,37**	6,61	0,24	6,46	0,09	7,08	0,71	6,96	0,59	6,98	0,61	6,76	0,39	7,29	0,92	7,03	0,66	7,11	0,74	6,97	0,60
5	**6,63**	6,67	0,04	6,52	0,11	6,35	0,28	6,28	0,35	6,66	0,03	6,48	0,15	6,72	0,09	6,51	0,12	6,43	0,20	6,33	0,30
7	**4,74**	4,97	0,23	4,93	0,19	4,47	0,27	4,53	0,21	4,68	0,06	4,66	0,08	4,40	0,34	4,40	0,34	4,51	0,23	4,54	0,20
8	**3,94**	3,23	0,71	3,29	0,65	3,38	0,56	3,50	0,44	3,24	0,70	3,36	0,58	3,39	0,55	3,47	0,47	3,37	0,57	3,47	0,47
10	**3,07**	2,63	0,44	2,73	0,34	2,19	0,88	2,39	0,68	2,26	0,81	2,46	0,61	2,27	0,80	2,46	0,61	2,56	0,51	2,72	0,35
10	**1,93**	1,54	0,39	1,71	0,22	1,41	0,52	1,66	0,27	1,46	0,47	1,73	0,20	1,59	0,34	1,84	0,09	1,51	0,42	1,73	0,20
11	**3,88**	3,58	0,30	3,62	0,26	3,98	0,10	4,07	0,19	3,79	0,09	3,85	0,03	3,78	0,10	3,83	0,05	3,57	0,31	3,66	0,22
13	**1,36**	1,36	0,00	1,53	0,17	1,33	0,03	1,58	0,22	1,32	0,04	1,60	0,24	1,38	0,02	1,65	0,29	1,34	0,02	1,57	0,21
14	**1,14**	1,34	0,20	1,51	0,37	1,31	0,17	1,56	0,42	1,21	0,07	1,50	0,36	1,22	0,08	1,51	0,37	1,29	0,15	1,53	0,39
15	**4,6**	4,81	0,21	4,78	0,18	4,56	0,04	4,60	0,00	4,55	0,05	4,55	0,05	4,61	0,01	4,59	0,01	4,58	0,02	4,60	0,00
17	**4,46**	4,38	0,08	4,37	0,09	4,20	0,26	4,27	0,19	4,37	0,09	4,39		4,25	0,21	4,26	0,20	4,26	0,20	4,31	0,15
17	**4,37**	4,21	0,16	4,21	0,16	4,19	0,18	4,26	0,11	4,20	0,17	4,23	0,14	4,15	0,22	4,17	0,20	4,15	0,22	4,20	0,17
**R^2**		**0,98779**		**0,98779**		**0,98147**		**0,98147**		**0,98500**		**0,98500**		**0,98262**		**0,98262**		**0,98509**		**0,98509**	
**MAE**			**0,26**		**0,24**		**0,32**		**0,30**		**0,28**		**0,27**		**0,31**		**0,28**		**0,30**		**0,28**

N°	Exp	8_6				8_7				8_8				8_9				8_10			
		unscaled	Δ	scaled	Δ	unscaled	Δ	scaled	Δ	unscaled	Δ	scaled	Δ	unscaled	Δ	scaled	Δ	unscaled	Δ	scaled	Δ
1	**1,81**	1,90	0,09	2,10	0,29	1,93	0,12	2,10	0,29	1,93	0,12	2,10	0,29	2,02	0,21	2,10	0,29	1,90	0,09	2,09	0,28
2	**5,85**	6,33	0,48	6,18	0,33	6,36	0,51	6,18	0,33	6,36	0,51	6,18	0,33	6,14	0,29	6,18	0,33	6,29	0,44	6,20	0,35
3	**6,15**	6,52	0,37	6,35	0,20	6,58	0,43	6,38	0,23	6,58	0,43	6,38	0,23	6,35	0,20	6,35	0,20	6,49	0,34	6,39	0,24
4	**6,37**	7,14	0,77	6,93	0,56	6,84	0,47	6,63	0,26	6,84	0,47	6,63	0,26	6,59	0,22	6,93	0,56	7,11	0,74	6,97	0,60
5	**6,63**	6,69	0,06	6,51	0,12	6,70	0,07	6,49	0,14	6,70	0,07	6,49	0,14	6,46	0,17	6,51	0,12	6,43	0,20	6,33	0,30
7	**4,74**	4,34	0,40	4,34	0,40	5,00	0,26	4,93	0,19	5,00	0,26	4,93	0,19	4,88	0,14	4,34	0,40	4,51	0,23	4,54	0,20
8	**3,94**	3,36	0,58	3,44	0,50	3,32	0,62	3,38	0,56	3,32	0,62	3,38	0,56	3,31	0,63	3,44	0,50	3,37	0,57	3,47	0,47
10	**3,07**	2,53	0,54	2,68	0,39	2,56	0,51	2,68	0,39	2,56	0,51	2,68	0,39	2,61	0,46	2,68	0,39	2,56	0,51	2,72	0,35
10	**1,93**	1,56	0,37	1,79	0,14	1,56	0,37	1,76	0,17	1,56	0,37	1,76	0,17	1,67	0,26	1,79	0,14	1,51	0,42	1,73	0,20
11	**3,88**	3,63	0,25	3,69	0,19	3,63	0,25	3,66	0,22	3,63	0,25	3,66	0,22	3,60	0,28	3,69	0,19	3,57	0,31	3,66	0,22
13	**1,36**	1,40	0,04	1,65	0,29	1,38	0,02	1,59	0,23	1,38	0,02	1,59	0,23	1,50	0,14	1,65	0,29	1,34	0,02	1,57	0,21
14	**1,14**	1,22	0,08	1,48	0,34	1,24	0,10	1,46	0,32	1,24	0,10	1,46	0,32	1,38	0,24	1,48	0,34	1,29	0,15	1,53	0,39
15	**4,6**	4,68	0,08	4,66	0,06	4,64	0,04	4,59	0,01	4,64	0,04	4,59	0,01	4,54	0,06	4,66	0,06	4,58	0,02	4,60	0,00
17	**4,46**	4,26	0,20	4,27	0,19	4,44	0,02	4,41	0,05	4,44	0,02	4,41	0,05	4,36	0,10	4,27	0,19	4,26	0,20	4,31	0,15
17	**4,37**	4,21	0,16	4,23	0,14	4,06	0,31	4,06	0,31	4,06	0,31	4,06	0,31	4,00	0,37	4,23	0,14	4,15	0,22	4,20	0,17
**R^2**		**0,98526**		**0,98526**		**0,98779**		**0,98779**		**0,98779**		**0,98779**		**0,98779**		**0,98526**		**0,98509**		**0,98509**	
**MAE**			**0,30**		**0,27**		**0,27**		**0,25**		**0,27**		**0,25**		**0,25**		**0,27**		**0,30**		**0,28**

Table S22. Comparison of ^{13}C NMR data of compound **8** and calculated ^{1}H NMR data of 10 possible isomers of **8** in $CDCl_3$.

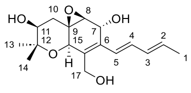

8_1

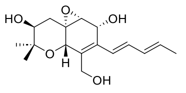

8_2

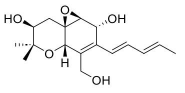

8_3

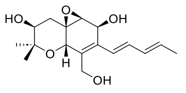

8_4

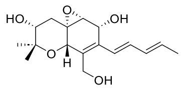

8_5

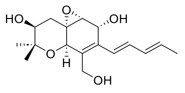

8_6

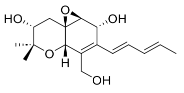

8_7

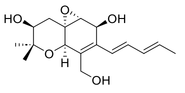

8_8

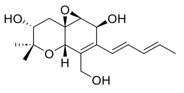

8_9

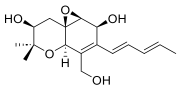

8_10

N°	Exp	8_1				8_2				8_3				8_4				8_5				8_6				8_7				8_8				8_9				8_10			
		unscaled	Δ	scaled	Δ	unscaled	Δ	scaled	Δ	unscaled	Δ	scaled	Δ	unscaled	Δ	scaled	Δ	unscaled	Δ	scaled	Δ	unscaled	Δ	scaled	Δ	unscaled	Δ	scaled	Δ	unscaled	Δ	scaled	Δ	unscaled	Δ	scaled	Δ	unscaled	Δ	scaled	Δ
1	**18,6**	21,0	2,4	23,1	4,5	21,0	2,4	22,6	4,0	21,1	2,5	23,0	4,4	21,1	2,5	22,4	3,8	21,0	2,4	22,2	3,6	20,9	2,3	21,8	3,2	21,0	2,4	22,3	3,7	21,0	2,4	22,3	3,7	20,9	2,3	21,8	3,2	21,0	2,4	22,2	3,6
2	**132,5**	133,4	0,9	136,9	4,4	133,3	0,8	135,5	3,0	134,0	1,5	137,0	4,5	134,7	2,2	136,2	3,7	133,4	0,9	135,8	3,3	133,8	1,3	135,7	3,2	134,9	2,4	138,4	5,9	134,9	2,4	138,4	5,9	133,8	1,3	135,7	3,2	133,4	0,9	135,8	3,3
3	**132,2**	129,2	3,0	132,6	0,4	130,1	2,1	132,4	0,2	129,3	2,9	132,3	0,1	130,0	2,2	131,5	0,7	129,7	2,5	132,1	0,1	129,2	3,0	131,1	1,1	128,9	3,3	132,2	0,0	128,9	3,3	132,2	0,0	129,2	3,0	131,1	1,1	129,7	2,5	132,1	0,1
4	**124,4**	128,7	4,3	132,1	7,7	133,3	8,9	135,6	11,2	132,6	8,2	135,6	11,2	135,7	11,3	137,2	12,8	134,3	9,9	136,8	12,4	136,6	12,2	138,5	14,1	131,0	6,6	134,4	10,0	131,0	6,6	134,4	10,0	136,6	12,2	138,5	14,1	134,3	9,9	136,8	12,4
5	**133,8**	123,1	10,7	126,4	7,4	122,2	11,6	124,4	9,4	122,1	11,7	125,0	8,8	123,8	10,0	125,3	8,5	121,9	11,9	124,3	9,5	123,5	10,3	125,3	8,5	122,3	11,5	125,5	8,3	122,3	11,5	125,5	8,3	123,5	10,3	125,3	8,5	121,9	11,9	124,3	9,5
6	**132**	133,4	1,4	136,9	4,9	136,9	4,9	139,1	7,1	135,1	3,1	138,1	6,1	139,4	7,4	140,9	8,9	136,1	4,1	138,6	6,6	138,3	6,3	140,3	8,3	134,6	2,6	138,1	6,1	134,6	2,6	138,1	6,1	138,3	6,3	140,3	8,3	136,1	4,1	138,6	6,6
7	**66,6**	68,0	1,4	70,7	4,1	70,7	4,1	72,5	5,9	68,2	1,6	70,6	4,0	71,0	4,4	72,4	5,8	70,6	4,0	72,3	5,7	71,2	4,6	72,5	5,9	67,2	0,6	69,4	2,8	67,2	0,6	69,4	2,8	71,2	4,6	72,5	5,9	70,6	4,0	72,3	5,7
8	**70,3**	58,8	11,5	61,4	8,9	63,7	6,6	65,5	4,8	63,7	6,6	66,1	4,2	65,1	5,2	66,5	3,8	62,2	8,1	63,9	6,4	65,4	4,9	66,7	3,6	63,8	6,5	65,9	4,4	63,8	6,5	65,9	4,4	65,4	4,9	66,7	3,6	62,2	8,1	63,9	6,4
9	**80,2**	60,8	19,4	63,3	16,9	63,4	16,8	65,2	15,0	58,8	21,4	61,1	19,1	62,5	17,7	63,9	16,3	62,6	17,6	64,3	15,9	60,9	19,3	62,1	18,1	56,6	23,6	58,5	21,7	56,6	23,6	58,5	21,7	60,9	19,3	62,1	18,1	62,6	17,6	64,3	15,9
10	**35,1**	35,9	0,8	38,2	3,1	38,5	3,4	40,1	5,0	38,3	3,2	40,4	5,3	38,4	3,3	39,8	4,7	36,3	1,2	37,7	2,6	37,9	2,8	39,0	3,9	37,5	2,4	39,1	4,0	37,5	2,4	39,1	4,0	37,9	2,8	39,0	3,9	36,3	1,2	37,7	2,6
11	**84,6**	76,4	8,2	79,2	5,4	75,6	9,0	77,6	7,0	76,9	7,7	79,4	5,2	76,4	8,2	77,8	6,8	76,5	8,1	78,4	6,2	76,2	8,4	77,6	7,0	76,7	7,9	79,1	5,5	76,7	7,9	79,1	5,5	76,2	8,4	77,6	7,0	76,5	8,1	78,4	6,2
12	**72,3**	82,2	9,9	85,1	12,8	81,9	9,6	83,8	11,5	80,8	8,5	83,3	11,0	80,4	8,1	81,8	9,5	82,3	10,0	84,2	11,9	80,6	8,3	82,0	9,7	81,4	9,1	83,8	11,5	81,4	9,1	83,8	11,5	80,6	8,3	82,0	9,7	82,3	10,0	84,2	11,9
13	**27,4**	26,9	0,5	29,0	1,6	28,6	1,2	30,2	2,8	16,3	11,1	18,3	9,1	16,4	11,0	17,7	9,7	22,0	5,4	23,2	4,2	26,6	0,8	27,5	0,1	22,4	5,0	23,7	3,7	22,4	5,0	23,7	3,7	26,6	0,8	27,5	0,1	22,0	5,4	23,2	4,2
14	**26,2**	22,0	4,2	24,2	2,0	15,9	10,3	17,5	8,7	28,5	2,3	30,5	4,3	28,4	2,2	29,7	3,5	26,9	0,7	28,2	2,0	22,3	3,9	23,2	3,0	26,8	0,6	28,2	2,0	26,8	0,6	28,2	2,0	22,3	3,9	23,2	3,0	26,9	0,7	28,2	2,0
15	**87,1**	74,6	12,5	77,3	9,8	74,8	12,3	76,7	10,4	73,5	13,6	76,0	11,1	74,8	12,3	76,2	10,9	74,9	12,2	76,7	10,4	73,8	13,3	75,1	12,0	75,3	11,8	77,6	9,5	75,3	11,8	77,6	9,5	73,8	13,3	75,1	12,0	74,9	12,2	76,7	10,4
16	**134,7**	134,3	0,4	137,8	3,1	131,6	3,1	133,8	0,9	131,8	2,9	134,8	0,1	129,1	5,6	130,6	4,1	131,9	2,8	134,4	0,3	130,7	4,0	132,5	2,2	131,4	3,3	134,9	0,2	131,4	3,3	134,9	0,2	130,7	4,0	132,5	2,2	131,9	2,8	134,4	0,3
17	**60,3**	61,4	1,1	64,0	3,7	63,8	3,5	65,6	5,3	64,3	4,0	66,7	6,4	66,9	6,6	68,3	8,0	63,3	3,0	65,0	4,7	66,1	5,8	67,4	7,1	65,1	4,8	67,3	7,0	65,1	4,8	67,3	7,0	66,1	5,8	67,4	7,1	63,3	3,0	65,0	4,7
R^2		**0,98457**		**0,98457**		**0,98266**		**0,98266**		**0,98061**		**0,98061**		**0,98056**		**0,98056**		**0,98305**		**0,98305**		**0,98091**		**0,98091**		**0,98119**		**0,98119**		**0,98119**		**0,98119**		**0,98091**		**0,98091**		**0,98305**		**0,98305**	
MAE			**5,46**		**5,93**		**6,48**		**6,60**		**6,62**		**6,76**		**7,08**		**7,16**		**6,16**		**6,23**		**6,57**		**6,53**		**6,15**		**6,25**		**6,15**		**6,25**		**6,57**		**6,53**		**6,16**		**6,23**

Functional	Solvent?	Basis Set	Type of Data
B3LYP	PCM	6-31+G(d,p)	Unscaled Shifts

	Isomer 1	Isomer 2	Isomer 3	Isomer 4	Isomer 5	Isomer 6	Isomer 7	Isomer 8	Isomer 9	Isomer 10
sDP4+ (H data)	36,91%	1,12%	10,41%	0,79%	0,17%	1,05%	16,45%	16,45%	16,45%	0,17%
sDP4+ (C data)	93,25%	0,00%	0,00%	0,00%	0,80%	0,04%	2,53%	2,53%	0,04%	0,80%
sDP4+ (all data)	97,61%	0,00%	0,00%	0,00%	0,00%	0,00%	1,18%	1,18%	0,02%	0,00%
uDP4+ (H data)	21,31%	0,90%	19,84%	1,10%	0,99%	2,12%	16,98%	16,98%	18,80%	0,99%
uDP4+ (C data)	9,02%	20,24%	12,11%	0,67%	11,73%	5,12%	12,12%	12,12%	5,12%	11,73%
uDP4+ (all data)	19,35%	1,83%	24,19%	0,07%	1,17%	1,09%	20,72%	20,72%	9,70%	1,17%
DP4+ (H data)	42,16%	0,05%	11,07%	0,05%	0,01%	0,12%	14,98%	14,98%	16,58%	0,01%
DP4+ (C data)	91,25%	0,01%	0,00%	0,00%	1,02%	0,02%	3,32%	3,32%	0,02%	1,02%
DP4+ (all data)	97,47%	0,00%	0,00%	0,00%	0,00%	0,00%	1,26%	1,26%	0,01%	0,00%

Figure S55. DP4+ probability analysis for the determination of the absolute configuration of **8**.

Table S23. ^{1}H and ^{13}C NMR (700 MHz) data of xylacremolide B (**9**) in $CDCl_3$.[a]

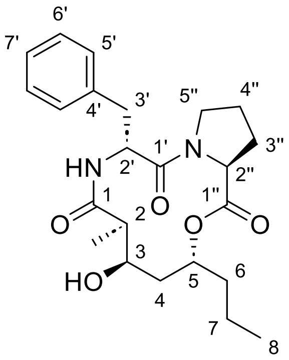

N°	δ_C	δ_H
1	174.3	
2	48.1	2.64 qd (6.7, 3.0)
3	68.5	4.21 dd (6.0, 3.0)
4	34.2	1.92 m,<br>1.57 dd (6.7, 3.0)
5	74.9	4.80 ddt (8.6, 6.0, 3.0)
6	33.1	1.81 m, 1.65 m
7	20.0	1.39 m, 1.30 m
8	14.8	0.91 m
2-Me	7.3	1.10 d (6.7)
1’	171.3	
2’	52.8	5.0 td (10.2, 5.8)
3’	36.5	3.14 m,<br>2.94 dd (13.1, 5.8)
4’	136.5	
5’, 9’	129.0	7.30 m
6’, 8’	129.0	7.22 m
7’	127.0	7.17 m
1’’	170.3	
2’’	58.1	4.75 d (7.1)
3’’	27.6	2.31 m,<br>1.71 m
4’’	23.5	1.71 m
5’’	46.3	3.96 m,<br>2.80 m

[a] Coupling constants (in parentheses) are in Hz.

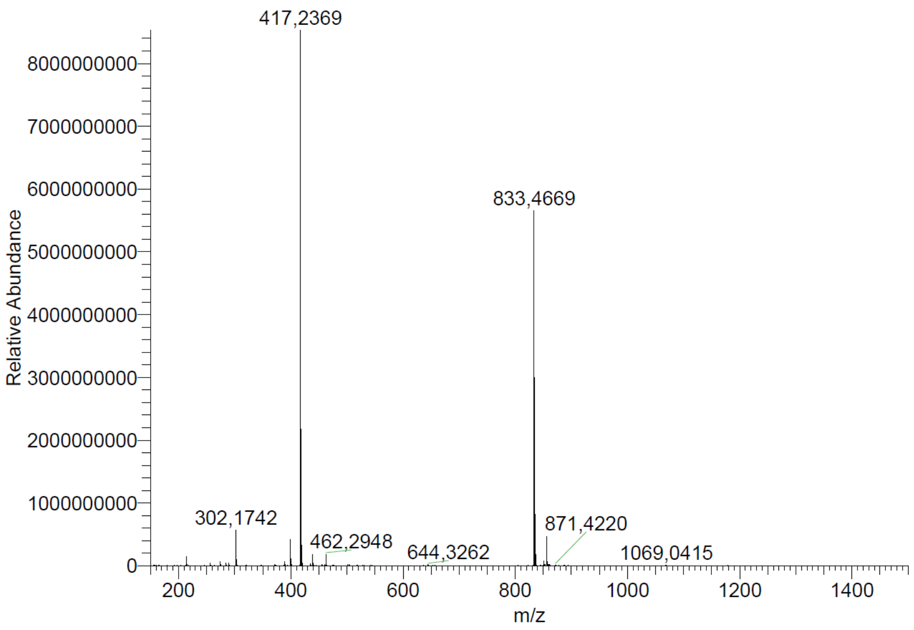

Figure S56. HR-ESIMS data of compound **9**.

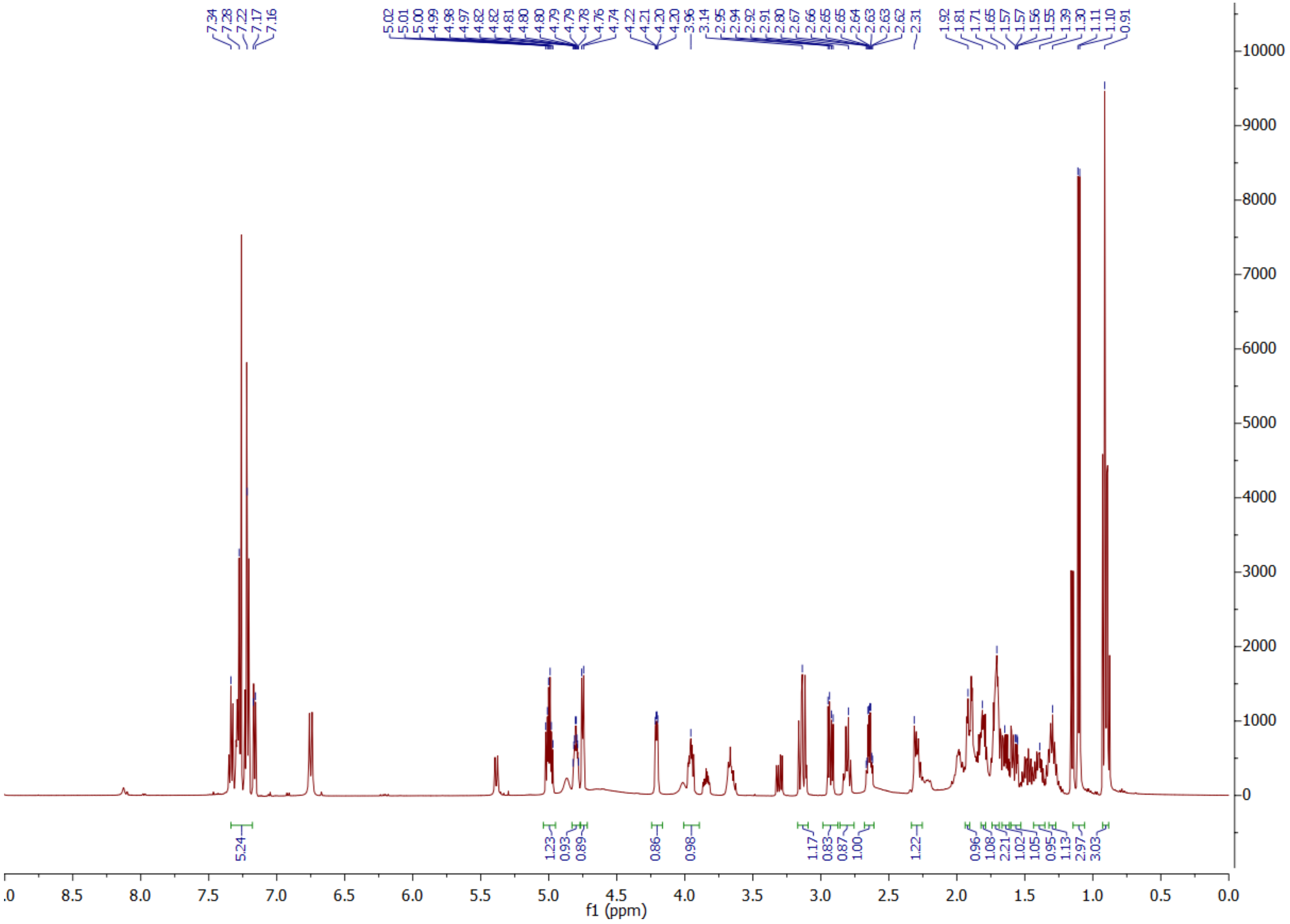

Figure S57. ^{1}H NMR spectrum of compound **9**.

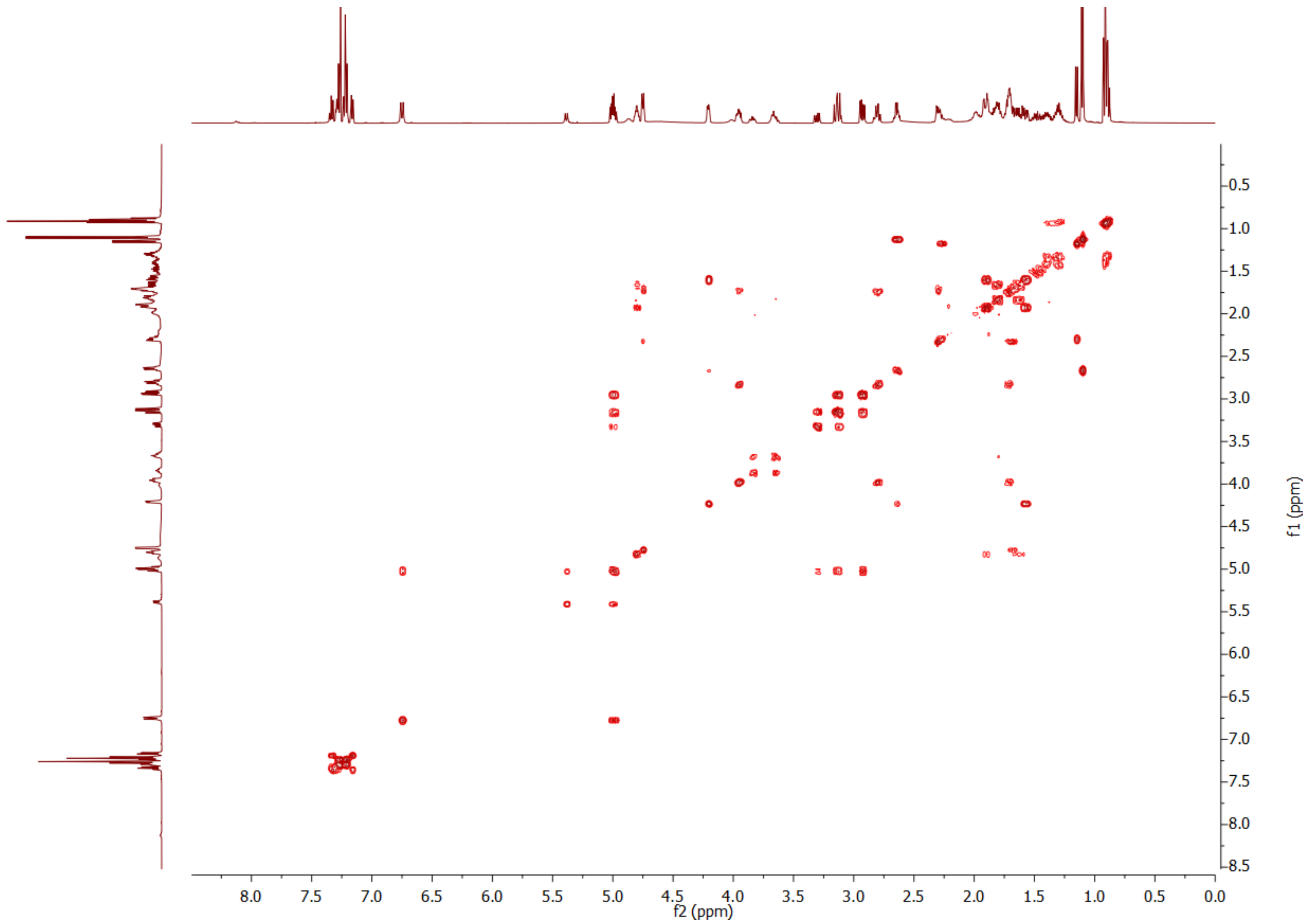

Figure S58. ^1H-^1H COSY spectrum of compound **9**.

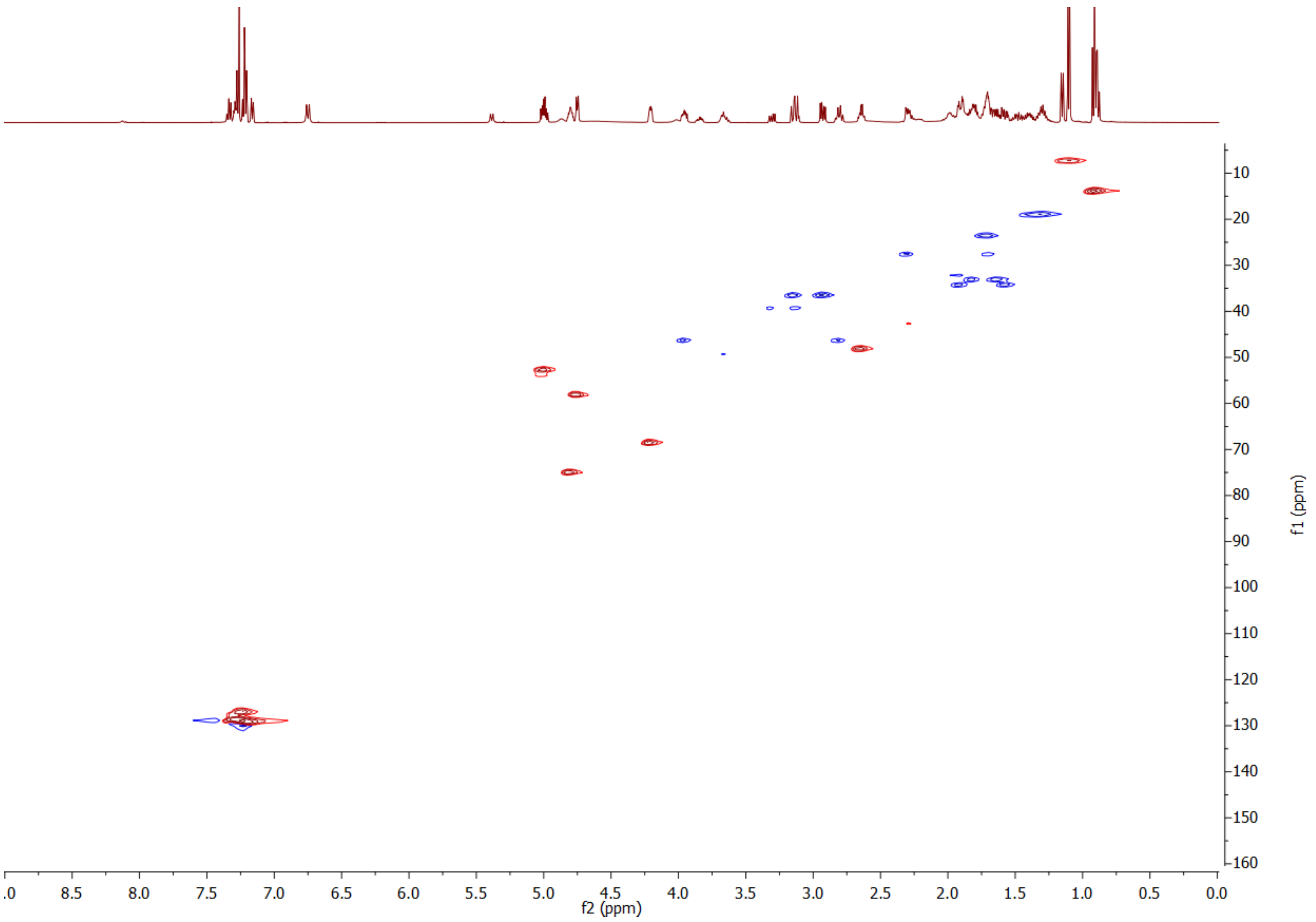

Figure S59. ^{1}H-^{13}C HSQC spectrum of compound **9**.

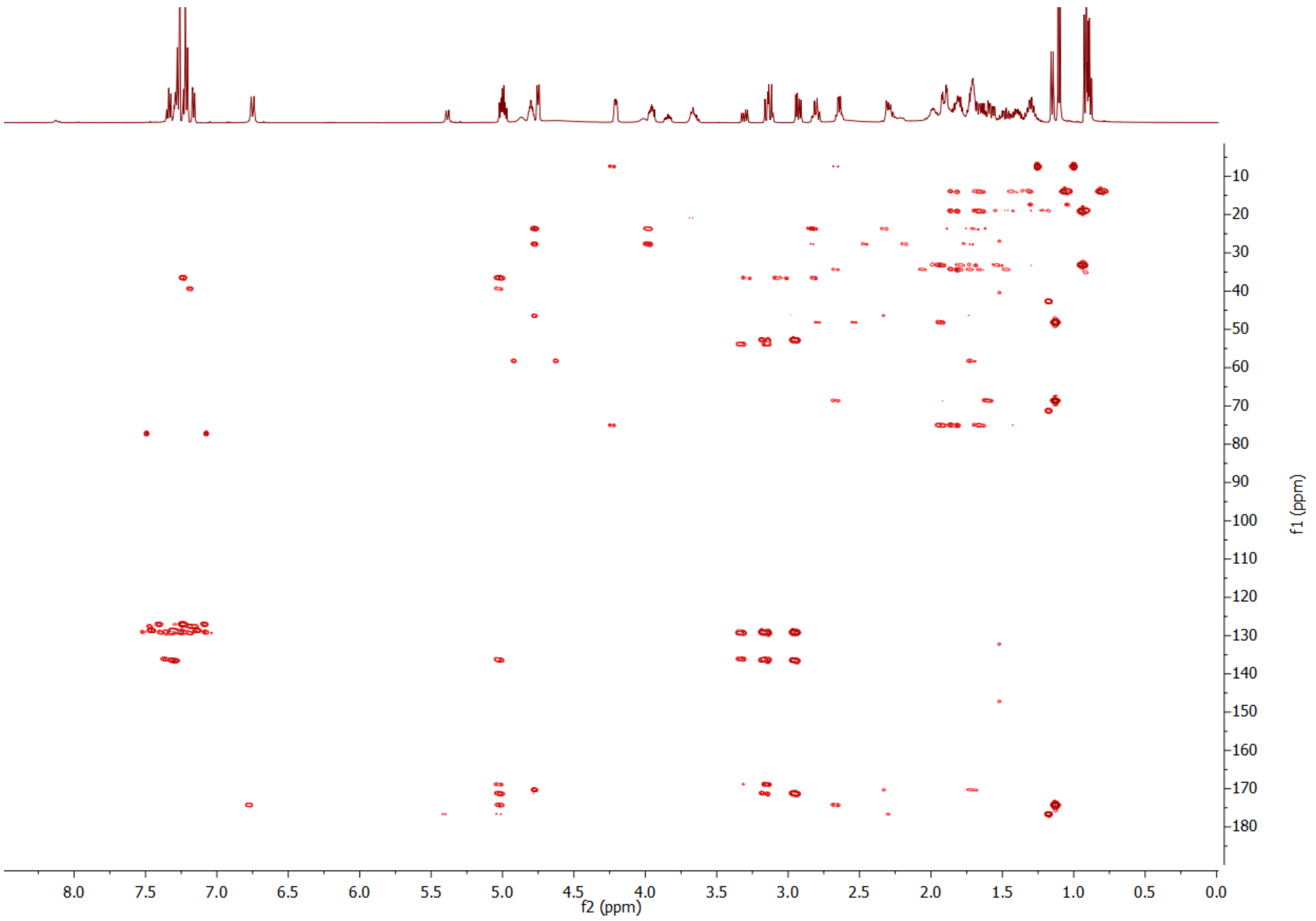

Figure S60. ^{1}H-^{13}C HMBC spectrum of compound **9**.

Table S24. Crystallography data. Crystal data and structure refinement for compound **9**.

Identification code	sh5538_a	
Empirical formula	C23 H32 N2 O5	
Formula weight	416.50	
Temperature	150(2) K	
Wavelength	0.71073 Å	
Crystal system	Orthorhombic	
Space group	$P2_12_12_1$	
Unit cell dimensions	a = 9.2915(3) Å	a= 90°.
	b = 10.7976(3) Å	b= 90°.
	c = 22.0427(6) Å	g = 90°.
Volume	2211.45(11) $Å^3$	
Z	4	
Density (calculated)	1.251 Mg/m^3	
Absorption coefficient	0.088 mm^{-1}	
F(000)	896	
Crystal size	0.200 x 0.060 x 0.060 mm^3	
Theta range for data collection	2.100 to 27.114°.	
Index ranges	-11<=h<=11, -13<=k<=13, -28<=l<=28	
Reflections collected	72893	
Independent reflections	4888 [R(int) = 0.0521]	
Completeness to theta = 25.242°	100.0 %	
Absorption correction	Semi-empirical from equivalents	
Max. and min. transmission	0.7455 and 0.7063	
Refinement method	Full-matrix least-squares on F^2	
Data / restraints / parameters	4888 / 2 / 279	
Goodness-of-fit on F^2	1.055	
Final R indices [I>2sigma(I)]	R1 = 0.0291, wR2 = 0.0695	
R indices (all data)	R1 = 0.0318, wR2 = 0.0718	
Absolute structure parameter	0.0(2)	
Extinction coefficient	n/a	
Largest diff. peak and hole	0.165 and -0.170 e.$Å^{-3}$	

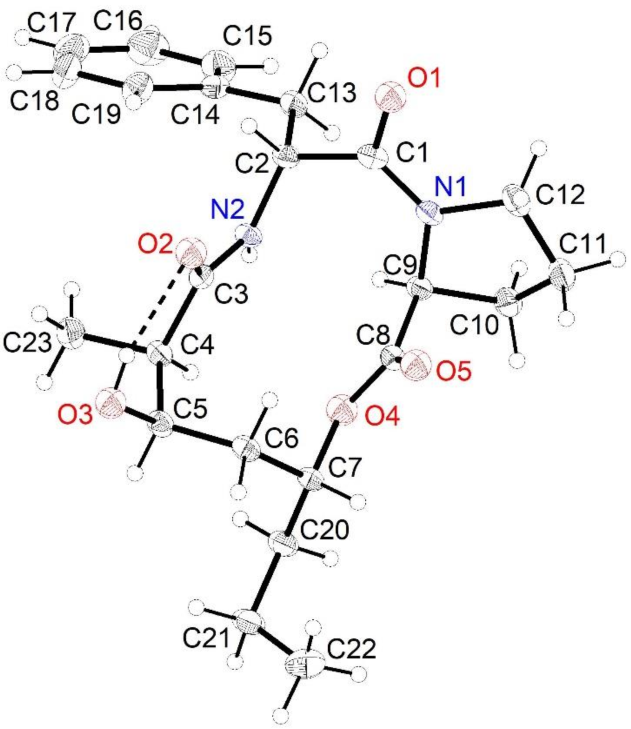

Figure S61. Refinement: All non H-atoms were located in the electron density maps and refined anisotropically. C-bound H atoms were placed in positions of optimized geometry and treated as riding atoms. Their isotropic displacement parameters were coupled to the corresponding carrier atoms by a factor of 1.2 (CH, CH2) or 1.5 (CH3). The O3 and N2 bonded H-atoms H3O and H2N were located in the electron density maps. Their positional parameters were refined using isotropic displacement parameters which were set at 1.2 (N2) and 1.5 (O3) times the Ueq value of the parent atoms. Restraints of 0.88(0.01) Å and 0.84(0.01) Å were used for the N-H and O-H bond lengths, respectively.

Table S25. *In silico* analysis of the *px* gene cluster within the genome of *Xylaria* sp. X802.Homologues biosynthetic gene clusters were identified from the genome of *Xylaria* sp. X802 (*Xylaria* sp. X802, NCBI Accession Nr: JAJFDH000000000) using the protein-protein BLAST (blastp) function of the NCBI. RNA-Seq data was mapped against the identified *px* gene cluster and expression levels were calculated using Geneious Prime 2021.0.3 (Biomatters, Ltd.).

Xylaria sp. X802			Predicted or verified function		Homologs (identities %/ similarities%)	
Protein	Size (aa)		*Aspergillus ruber* CBS 135680	*Trichoderma virens* Gv29-8	*Neurospora crassa* OR74A	Other *Xylariales*
PxA	2420	HR-PKS (KS_AT_DH_ER_KR_ACP)	FogA (62/75)	VirA (53/69)	SrdA (41/58)	ORY71075.1 (66/79)
PxB	273	Short-chain dehydrogenase / reductase	FogB (57/75)	VirB (46/61)	SrdC (49/69)	GAP88226.1 (67/82)
PxC	209	Cupin fold protein	FogC (60/76)	VirC (56/74)	SrdD (49/71)	RYC64331.1 (76/86)
PxD	291	Short-chain dehydrogenase / reductase	FogD (79/86)	VirD (68/80)	SrdE (66/76)	ORY71073.1 (82/90)
PxE	542	Cytochrome P450 monooxygenase	FogE (56/78)	VirE (55/73)	-	ORY71072.1 (69/86)
PxF	191	Cupin fold protein	-	-	SrdB (71/84)	ORY71067.1 (77/88)
PxG	729	FAD-dependent monooxygenase	-	-	SrdH (28/44)	GAP90730.1 (62/74)
PxH	430	Aromatic prenyltransferase	FogH (24/42)	-	-	RYC64352.1 (61/75)
PxI	519	FAD/FMN-dependent dehydrogenase	FogF (27/44)	VirI (48/66)	SrdI (27/47)	RYC64351.1 (65/80)
PxJ	274	Short-chain dehydrogenase / reductase	-	VirK (55/71)	-	TRX89882.1 (73/88)
PxK	474	Fungal transcription factor	-	-	-	TRX89881.1 (60/74)
PxL	574	MFS transporter	-	-	-	RYC56959.1 (72/85)
PxM	601	Fungal transcription factor	-	-	-	GAP88230.2 (54/68)
PxN	321	Phosphopantetheinyl transferase	-	-	-	TRX98604.1 (79/88
PxO	343	Thioesterase	-	-	-	GAP92521.2 (81/87)

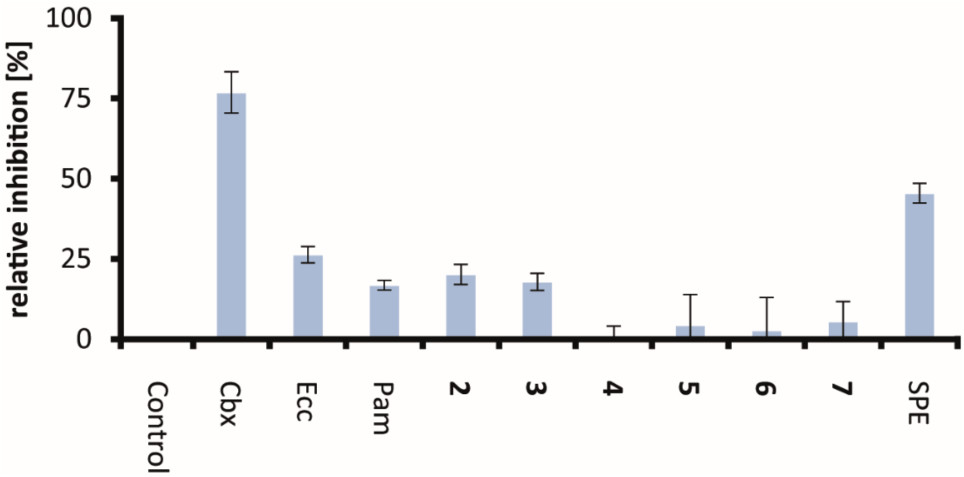

Figure S62. Antifungal activity assay of **2**-**7** against *Aspergillus nidulans* RMS011 (n = 3).

Supplementary references

1. Ciavatta, M. L. *et al.* Cytosporin-related compounds from the marine-derived fungus Eutypella scoparia. *Tetrahedron* **64**, 5365–5369 (2008).
2. Rivera-Chávez, J. *et al.* Cuautepestalorin, a 7,8-Dihydrochromene–Oxoisochromane Adduct Bearing a Hexacyclic Scaffold from *Pestalotiopsis* sp. IQ-011. *Org. Lett.* **21**, 3558–3562 (2019).
