# Supplementary material for: Molecular networking and computational NMR analyses uncover six polyketide-terpene hybrids from termite-associated Xylaria isolates
Source: Commun Chem. 2024 Jun 7;7:129. doi: 10.1038/s42004-024-01210-6 (PMC11161606; doi:10.1038/s42004-024-01210-6)
Supplement: Supplementary file 3 — Description of Additional Supplementary Files [file 42004_2024_1210_MOESM3_ESM.pdf]

Description of Additional Supplementary Files

File name- Supplementary Data 1

File description- Contains lists of calculated 1H and 13C chemical shifts for each determined conformer.

File name- Supplementary Data 2

File description- Crystallographic Information File (CIF) for structure of compound 9. The X-ray crystallographic coordinates for structure of compound 9 reported in this study have also been deposited at the Cambridge Crystallographic Data Centre (CCDC), under deposition numbers CCDC-2347670. These data can be obtained free of charge from The Cambridge Crystallographic Data Centre via www.ccdc.cam.ac.uk/data_request/cif.
